# Positively correlated miRNA-mRNA regulatory networks in mouse frontal cortex during early stages of alcohol dependence

**DOI:** 10.1186/1471-2164-14-725

**Published:** 2013-10-22

**Authors:** Yury O Nunez, Jay M Truitt, Giorgio Gorini, Olga N Ponomareva, Yuri A Blednov, R Adron Harris, R Dayne Mayfield

**Affiliations:** 1 The Waggoner Center for Alcohol and Addiction Research, The University of Texas at Austin, Austin, Texas, USA

**Keywords:** miRNA, mRNA, Expression profiling, WGCNA, Network analysis, Mouse, Human, Alcoholism, Alcohol dependence, Frontal cortex

## Abstract

**Background:**

Although the study of gene regulation via the action of specific microRNAs (miRNAs) has experienced a boom in recent years, the analysis of genome-wide interaction networks among miRNAs and respective targeted mRNAs has lagged behind. MicroRNAs simultaneously target many transcripts and fine-tune the expression of genes through cooperative/combinatorial targeting. Therefore, they have a large regulatory potential that could widely impact development and progression of diseases, as well as contribute unpredicted collateral effects due to their natural, pathophysiological, or treatment-induced modulation. We support the viewpoint that whole mirnome-transcriptome interaction analysis is required to better understand the mechanisms and potential consequences of miRNA regulation and/or deregulation in relevant biological models. In this study, we tested the hypotheses that ethanol consumption induces changes in miRNA-mRNA interaction networks in the mouse frontal cortex and that some of the changes observed in the mouse are equivalent to changes in similar brain regions from human alcoholics.

**Results:**

miRNA-mRNA interaction networks responding to ethanol insult were identified by differential expression analysis and weighted gene coexpression network analysis (WGCNA). Important pathways (coexpressed modular networks detected by WGCNA) and hub genes central to the neuronal response to ethanol are highlighted, as well as key miRNAs that regulate these processes and therefore represent potential therapeutic targets for treating alcohol addiction. Importantly, we discovered a conserved signature of changing miRNAs between ethanol-treated mice and human alcoholics, which provides a valuable tool for future biomarker/diagnostic studies in humans. We report positively correlated miRNA-mRNA expression networks that suggest an adaptive, targeted miRNA response due to binge ethanol drinking.

**Conclusions:**

This study provides new evidence for the role of miRNA regulation in brain homeostasis and sheds new light on current understanding of the development of alcohol dependence. To our knowledge this is the first report that activated expression of miRNAs correlates with activated expression of mRNAs rather than with mRNA downregulation in an *in vivo* model. We speculate that early activation of miRNAs designed to limit the effects of alcohol-induced genes may be an essential adaptive response during disease progression.

## Background

Alcohol dependence is a brain disorder of complex etiology characterized by brain-wide pathophysiological alterations under continuous crosstalk, with approximately 40-60% contribution from genetic factors [1]. As suggested by Farris and Miles, interacting neuronal and glial networks across distinct brain regions likely control the variety of alcohol endophenotypes, which are ultimately controlled by the regulation of multiple gene networks expressed within individual neurons or glial cells [2]. Support for these statements is found in the observation that chronic consumption of alcohol induces long-term changes in brain gene and protein expression, which allow brain cells to adapt through homeostatic alterations in distinct signaling pathways [3,4]. Although the complex regulatory mechanisms governing these changes are not fully understood, there is mounting evidence that transcriptional reprogramming is brain area-specific and may reflect both pre-existing differences in gene expression and alterations in response to alcohol consumption [5].

MicroRNAs (miRNAs), a type of short non-coding RNA with well characterized post-transcriptional regulatory functions, have been recently implicated in cellular responses to multiple drugs of abuse including alcohol [4,6-10]. Moreover, miRNAs have extensive regulatory capacity given that a single miRNA can simultaneously target multiple genes, and multiple miRNAs can cooperatively function while targeting a single gene, therefore allowing for fine-tuned regulation of targeted gene expression. We have previously shown that miRNAs, which localize to and display neurotransmitter signaling-related activities at neuronal synapses, are also capable of eliciting distinct and specific activities in other cell types and/or compartments of the cell, such as innate immunity- and epigenetic-related functions in neuronal and glial cells [4]. This underscores the impact that molecular efficiency, signaling crosstalk, and cellular economy play in the adaptation and evolution of cellular systems and highlights the significance of miRNAs as efficient molecular targets for complex diseases such as addiction.

We have previously reported that in brains of human alcoholics, miRNAs appear mostly upregulated, with an overrepresented number of targets among downregulated mRNAs. Approximately 80% of those targets appeared to be combinatorially regulated by multiple miRNAs [11]. Many of these upregulated miRNAs appear to be central regulators of epigenetic-, synaptic signaling-, and neuroimmune-related processes (Nunez and Mayfield [4]). Here we describe new findings in the frontal cerebral cortex (FCtx) of a mouse model for high voluntary ethanol consumption. Mouse models like ours, implementing drinking in the dark (DID) protocols that produce pharmacologically relevant levels of ethanol in blood, have been developed to examine binge-like ethanol consumption [12]. Binge ethanol consumption in humans is “a pattern of behaviour that may emerge prior to, and contribute to the development of, ethanol dependence” [12]. Our goal is to identify sets of miRNAs and relevant regulatory networks that contribute to our understanding of the mechanisms related to alcohol dependence in humans. We tested the hypotheses that alcohol induces changes in miRNA-mRNA interaction networks in mouse frontal cortex and that some of the changes observed in the mouse brain are equivalent to changes occurring in similar brain regions from human alcoholics. We report on the integrative analysis of genome-wide miRNA-mRNA expression profiles from mouse FCtx samples and on the implementation of weighted gene coexpression network analysis (WGCNA). Using WGCNA, we generated correlations among coexpressed gene modules and differentially expressed miRNAs and provided evidence for relevant regulatory networks responsive to alcohol actions. We unexpectedly uncovered striking positive correlation patterns between upregulated miRNAs and upregulated predicted, validated, and/or correlated mRNA targets.

## Results

### Differential expression analysis underscores a major role for upregulation of miRNAs in FCtx of ethanol-treated mice

**Figure 1:**
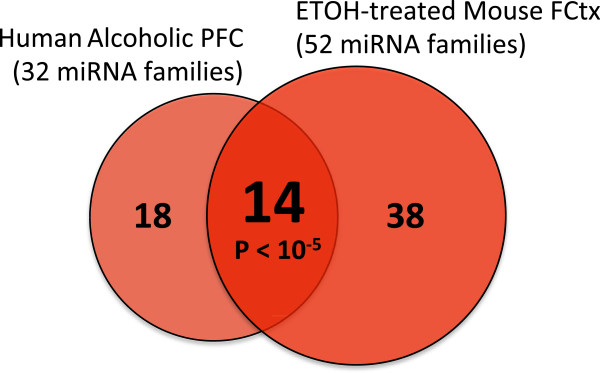
**Upregulation of frontal cortex miRNAs in response to alcohol is conserved from mice to humans.** Venn diagram highlights common set of 14 upregulated miRNA families in prefrontal cortex of human alcoholics [11] and ethanol-treated mice (current study). P value empirically assessed after 100,000 Monte Carlo simulations. PFC: prefrontal cortex, FCtx: frontal cortex.

In order to evaluate the impact of alcohol consumption on miRNA and gene expression in mouse brain, we conducted miRNA and mRNA profiling studies on 32 samples (20 ethanol-treated mice and 12 matched controls) as described in Materials and Methods. Ethanol-treated mice consumed an average of 4.92 g/kg/3 h (range: 4.08-6.31 g/kg/3 h; stdev: 0.52). The miRNA expression analysis indicated that, as in prefrontal cortex (PFC) of human alcoholics, miRNAs appear predominantly upregulated in FCtx of alcohol-drinking mice, with 52 miRNA families upregulated in mouse brain (Table 1). Importantly, we found that a highly significant number of miRNA families that are upregulated in PFC of human alcoholics [11] are also upregulated in the FCtx of ethanol-treated mice (Figure 1). Fourteen out of 32 alcohol-induced human miRNA families changed expression in the ethanol-treated mice (P < 1 × 10^-5^ as determined after 10,000 Monte Carlo simulations). The fact that upregulation is predominant at the significance level chosen (FDR < 10%) among differentially expressed miRNAs in the mouse frontal cortex is consistent with previous results in human alcoholic PFC [11]. The miRNA families that change expression in both mouse and human were: let-7, miR-7, miR-15, miR-101, miR-140, miR-152 (all validated by qPCR, P < 0.05), as well as miR-17, miR-34, miR-135, miR-144, miR-146, miR-301, miR-339, miR-368 (qPCR not performed). Other differentially expressed miRNAs specific to the mouse model were also validated by qPCR, including miR-195 (member of the miR-15 family) and miR-541 family members. Moreover, we found a significant match among differentially expressed mouse miRNA families and those reported in PFC of ethanol-treated rats [13]. Seventeen out of 33 rat miRNA families (representing 41 differentially expressed rat miRNAs) were matched to our mouse data, P < 1 × 10^-5^. The miRNA families that change expression in both mice and rats were: mir-7, mir-9, mir-10, mir-15, mir-17, mir-26, mir-29, mir-30, mir-101, mir-130, mir-181, mir-204, mir-339, mir-340, mir-368, mir-434, mir-467. Overall, these results underscore the relevance of gene regulation by miRNAs in response to alcohol consumption and suggest conservation of alcohol-responsive miRNA regulatory pathways from rodent to human.

**Table 1:** Differentially expressed miRNAs in mouse frontal cortex

**Rank**	**Name**	**Family ID**	**logFC**	**Ave.Exp.**	**P.Value**	**adj.P.Val**
1	mmu-let-7g-5p	let-7	0.30	12.20	2.12E-07	1.27E-04
2	mmu-let-7d-5p	let-7	0.27	11.53	5.98E-07	1.27E-04
4	mmu-let-7c-5p	let-7	0.29	11.78	1.09E-06	1.49E-04
5	mmu-let-7b-5p	let-7	0.25	12.48	1.17E-06	1.49E-04
10	mmu-let-7a-5p	let-7	0.27	12.59	9.73E-06	6.88E-04
7	mmu-let-7i-5p	let-7	0.24	11.17	1.08E-05	6.88E-04
22	mmu-let-7e-5p	let-7	0.21	11.25	1.67E-04	4.83E-03
42	mmu-miR-125a-5p	mir-10	0.22	11.79	1.49E-03	2.26E-02
12	mmu-miR-101b-3p	mir-101	0.29	7.79	1.72E-05	9.11E-04
37	mmu-miR-101a-3p	mir-101	0.24	10.12	1.17E-03	1.92E-02
50	mmu-miR-107-3p	mir-103	0.22	8.77	3.24E-03	4.12E-02
64	mmu-miR-124-5p	mir-124	0.15	6.32	7.13E-03	7.09E-02
33	mmu-miR-301a-3p	mir-130	0.22	8.39	6.90E-04	1.29E-02
59	mmu-miR-130a-3p	mir-130	0.16	8.30	5.94E-03	6.44E-02
52	mmu-miR-135b-5p	mir-135	0.22	7.92	4.08E-03	4.99E-02
74	mmu-miR-136-5p	mir-136	0.22	9.06	1.09E-02	9.39E-02
18	mmu-miR-138-5p	mir-138	0.26	12.84	9.05E-05	3.20E-03
40	mmu-miR-140-3p	mir-140	0.19	7.89	1.23E-03	1.94E-02
46	mmu-miR-144-3p	mir-144	0.25	6.88	2.38E-03	3.30E-02
68	mmu-miR-145-5p	mir-145	0.17	7.03	8.25E-03	7.73E-02
48	mmu-miR-146b-5p	mir-146	0.16	7.50	2.60E-03	3.38E-02
25	mmu-miR-152-3p	mir-148	0.19	6.47	2.22E-04	5.65E-03
31	mmu-miR-149-5p	mir-149	0.22	7.69	4.26E-04	8.75E-03
14	mmu-miR-16-5p	mir-15	0.29	10.94	2.74E-05	1.25E-03
16	mmu-miR-15a-5p	mir-15	0.20	9.18	5.63E-05	2.24E-03
20	mmu-miR-195-5p	mir-15	0.24	8.63	1.26E-04	4.01E-03
53	mmu-miR-15b-5p	mir-15	0.15	6.85	4.15E-03	4.99E-02
44	mmu-miR-93-5p	mir-17	0.16	6.98	1.73E-03	2.45E-02
21	mmu-miR-181d-5p	mir-181	0.24	8.47	1.51E-04	4.59E-03
57	mmu-miR-181a-5p	mir-181	0.20	9.81	5.96E-03	6.44E-02
69	mmu-miR-182-5p	mir-182	0.62	8.71	8.90E-03	8.21E-02
62	mmu-miR-183-5p	mir-183	0.66	8.38	6.90E-03	6.99E-02
49	mmu-miR-185-5p	mir-185	0.19	8.91	2.60E-03	3.38E-02
41	mmu-miR-191-5p	mir-191	0.19	9.71	1.25E-03	1.94E-02
28	mmu-miR-193-3p	mir-193	0.25	6.55	3.70E-04	8.12E-03
29	mmu-miR-1952	mir-1952	0.21	9.18	3.70E-04	8.12E-03
72	mmu-miR-1961	mir-1961	0.19	6.58	1.03E-02	9.11E-02
34	mmu-miR-204-5p	mir-204	0.31	7.74	6.87E-04	1.29E-02
26	mmu-miR-222-3p	mir-221	0.23	10.22	2.37E-04	5.81E-03
35	mmu-miR-221-3p	mir-221	0.25	9.09	8.47E-04	1.54E-02
63	mmu-miR-221-5p	mir-221	0.17	5.61	6.79E-03	6.99E-02
38	mmu-miR-23a-3p	mir-23	0.22	10.57	1.12E-03	1.92E-02
51	mmu-miR-23b-3p	mir-23	0.27	11.85	3.70E-03	4.63E-02
13	mmu-miR-24-2-5p	mir-24	0.24	7.13	1.89E-05	9.26E-04
24	mmu-miR-24-3p	mir-24	0.25	11.70	1.98E-04	5.27E-03
58	mmu-miR-24-1-5p	mir-24	0.17	7.72	5.92E-03	6.44E-02
3	mmu-miR-26a-5p	mir-26	0.30	12.79	4.71E-07	1.27E-04
47	mmu-miR-26b-5p	mir-26	0.29	9.79	2.47E-03	3.35E-02
15	mmu-miR-29a-5p	mir-29	0.29	7.56	3.17E-05	1.34E-03
6	mmu-miR-30d-5p	mir-30	0.26	9.68	3.57E-06	3.79E-04
39	mmu-miR-30e-5p	mir-30	0.26	10.87	1.18E-03	1.92E-02
43	mmu-miR-30e-3p	mir-30	0.29	8.17	1.61E-03	2.38E-02
76	mmu-miR-30b-5p	mir-30	0.25	11.79	1.12E-02	9.39E-02
9	mmu-miR-320-3p	mir-320	0.26	7.49	1.07E-05	6.88E-04
71	mmu-miR-330-5p	mir-330	0.15	7.70	9.69E-03	8.70E-02
30	mmu-miR-335-5p	mir-335	0.23	10.51	4.00E-04	8.50E-03
67	mmu-miR-339-5p	mir-339	0.20	6.80	7.92E-03	7.53E-02
8	mmu-miR-34c-5p	mir-34	0.24	6.68	9.54E-06	6.88E-04
77	mmu-miR-34a-5p	mir-34	0.17	9.54	1.17E-02	9.66E-02
45	mmu-miR-340-5p	mir-340	0.17	8.51	1.71E-03	2.45E-02
17	mmu-miR-361-5p	mir-361	0.24	7.88	7.74E-05	2.90E-03
19	mmu-miR-376b-3p	mir-368	0.26	8.45	1.05E-04	3.50E-03
56	mmu-miR-376a-3p	mir-368	0.19	10.21	5.63E-03	6.40E-02
23	mmu-miR-434-3p	mir-434	0.22	10.46	1.76E-04	4.88E-03
27	mmu-miR-451	mir-451	0.33	10.72	2.79E-04	6.57E-03
66	mmu-miR-669c-5p	mir-467	0.17	8.84	7.68E-03	7.41E-02
55	mmu-miR-485-3p	mir-485	0.19	6.75	5.52E-03	6.39E-02
65	mmu-miR-669n	mir-669n	0.14	7.88	7.46E-03	7.31E-02
60	mmu-miR-674-5p	mir-674	0.17	8.64	6.08E-03	6.45E-02
54	mmu-miR-676-3p	mir-676	0.15	6.63	4.48E-03	5.28E-02
11	mmu-miR-7a-5p	mir-7	0.24	9.17	1.37E-05	7.91E-04
36	mmu-miR-708-5p	mir-708	0.17	9.38	9.09E-04	1.61E-02
70	mmu-miR-429-3p	mir-8	0.61	9.27	9.53E-03	8.68E-02
32	mmu-miR-9-3p	mir-9	0.22	12.96	6.43E-04	1.28E-02
61	mmu-miR-9-5p	mir-9	0.24	12.11	6.91E-03	6.99E-02
73	mmu-miR-96-5p	mir-96	0.53	8.58	1.06E-02	9.22E-02

### Ethanol-responsive genes in brains of mice significantly match respective homologs differentially expressed in brains of human alcoholics

**Figure 2:**
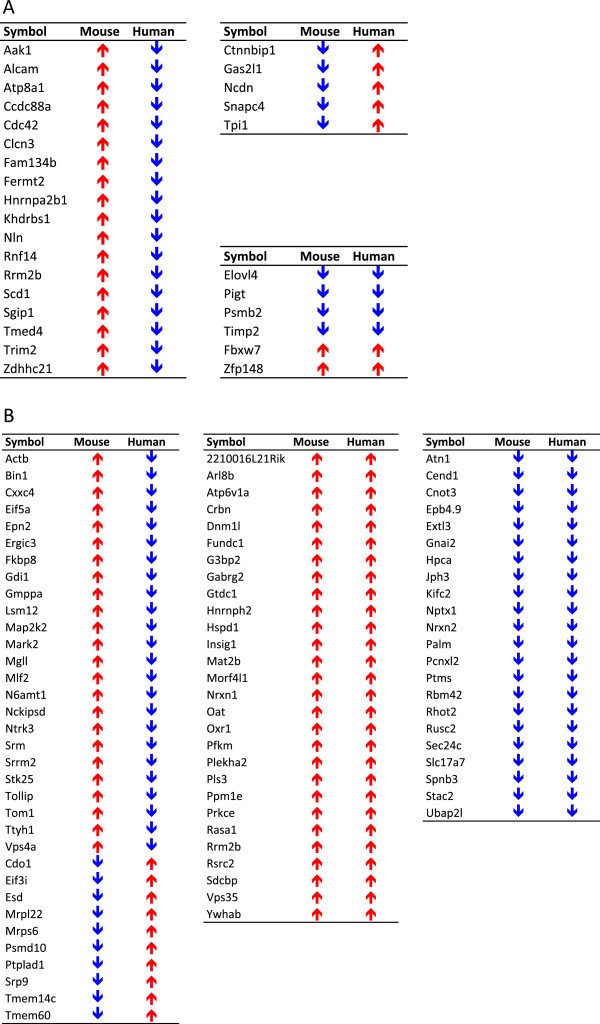
**Conserved differential gene expression in response to ethanol with implicit model differences in direction of change. A**: Venn diagram highlights common set of 29 differentially expressed genes in prefrontal cortex of human alcoholics (as reported by [14]) and ethanol-treated mice (current results); **B**: Directionality of expression changes in common set of 29 differentially expressed genes referred to in A; **C**: Venn diagram highlights common set of 84 differentially expressed genes in prefrontal cortex of human alcoholics (as reported by [15]) and ethanol-treated mice (current study); D: Directionality of expression change in common set of 84 differentially expressed genes referred to in C. P values empirically assessed after 10^5^ and 10^7^ Monte Carlo simulations, respectively.

Differential expression analysis of the Illumina microarray data identified 709 genes with expression changes in response to alcohol in the FCtx of ethanol-treated mice (Additional file 1: Table S1, FDR < 1%). A representative group of altered genes, including Syt11, Tom1, Atp2b1, and Fermt2, were validated by qPCR (P < 0.05) using TaqMan® assays as described in Materials and Methods. A significant number of the differentially expressed genes (Additional file 1: Figure S1A) also changed in PFC of human alcoholics, as reported by the Mayfield group [11,14]. Such highly statistically significant matches (P < 1 × 10^-5^ as empirically estimated by Monte Carlo simulations) underscore the relevance of the commonly affected genes. However, it should be noted that 79% of the common gene changes are occurring in opposite directions when the mouse and human models are compared (Figure 2A). When our mouse gene expression dataset was compared to an additional profiled cohort of human alcoholics [15], we consistently found a highly significant match among differentially expressed genes between the mouse and human models (84 common differentially expressed genes, P < 1 × 10^-7^, (Additional file 1: Figure S1B). Moreover, we similarly observed that a relatively large percentage (40%) changed in opposite directions (Figure 2B). Furthermore, comparison to the human datasets indicated that a majority of the common differentially expressed genes (65% on average) are upregulated in the mouse brain, while only 34% on average are upregulated in the brain of human alcoholics. These results suggest that brain genes upregulated in early stages of development of alcohol dependence (using mouse model) might undergo downregulation in late stages of the disease (using human alcoholic model), possibly due to homeostatic mechanisms driven by miRNA regulation.

### Integrated miRNA-mRNA networks reveal positively correlated action of brain miRNAs and persistent upregulation of overtargeted mRNAs in ethanol drinking mice

**Figure 3:**
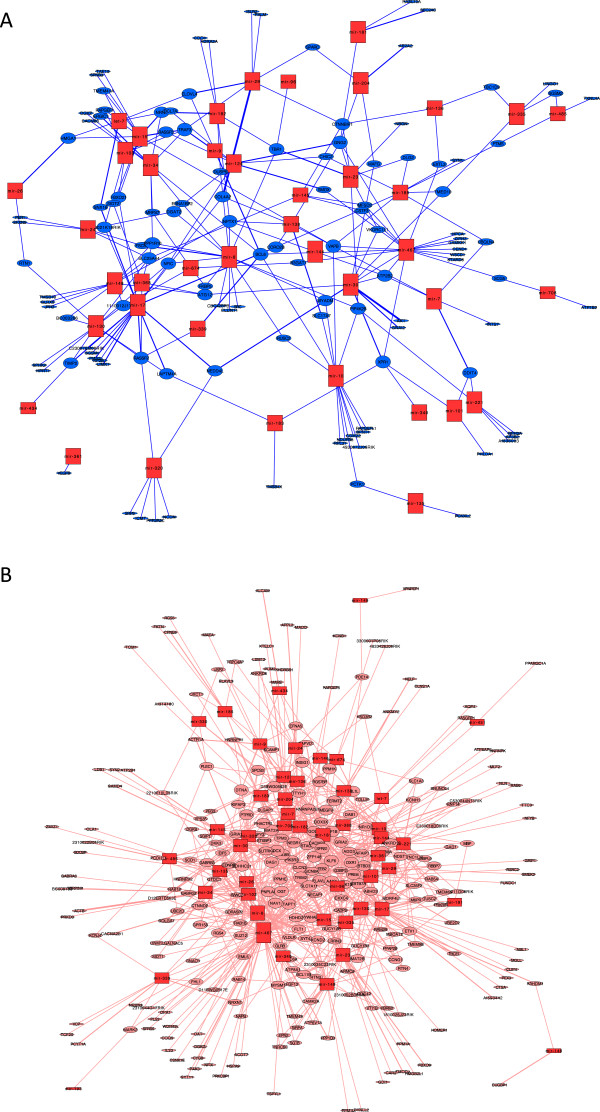
**Alcohol-induced miRNA-mRNA interaction networks. A**: Interaction network among upregulated miRNAs (red squares) and downregulated mRNAs (blue circles); average number of neighbors 2.95. **B**: Interaction network among upregulated miRNAs (red squares) and upregulated mRNAs (pink circles); average number of neighbors 4.97. The average number of neighbors represents the average number of links (edges) a node has to other nodes. The size of the nodes is proportional to the number of edges (interactions, represented as lines) for each node.

In order to better understand miRNA-mRNA regulatory relationships, we constructed respective interaction networks among negatively and positively correlated interaction groups, using the miRNA-mRNA interaction universe generated from miRecords predicted (consensus of at least 4 tools) and validated interactions (described in Materials and Methods). We found that the negatively correlated network of interactions among upregulated miRNAs and downregulated mRNAs (average number of neighbors: 2.95, Figure 3A) was no different from randomized networks generated as a control tool (average number of neighbors: 3.03, shown in Additional file 1: Figure S2A). On the other hand, the corresponding network containing the miRNA-mRNA interactions that were positively correlated (upregulated miRNA-upregulated mRNA network) provided unexpected evidence that this network was twice as interconnected (average number of neighbors: 4.97, Figure 3B) as expected by chance (average number of neighbors: 2.52, shown in Additional file 1: Figure S2B).

In addition when overrepresentation analyses were conducted on the interaction networks, we found that the majority of upregulated miRNAs (38/48 = 79%) have an overrepresented number of targets among upregulated genes (Additional file 1: Table S2, FDR < 0.10), while only about one quarter (10/41 = 24%) had an overrepresented number of targets among downregulated genes (Additional file 1: Table S3, FDR < 0.10). The expected number of miRNAs with overrepresented number of targets is 22/45 = 49%, as determined by averaging the proportions obtained for two random networks. Therefore, the proportion detected in the positively correlated (upregulated miRNA-upregulated mRNA) network is highly significantly enriched (79% vs. 49%, Chi-squared P < 1 × 10^-4^) in miRNAs that have a higher than expected number of targets (“overtargeting” miRNAs). On the other hand, the proportion detected in the negatively correlated (upregulated miRNA-downregulated mRNA) network is significantly smaller than expected by chance (24% vs. 49%, Chi-squared P = 0.0016), indicating that the negatively correlated network is depleted of overtargeting miRNAs. This is consistent with the observation that out of 413 upregulated genes, 265 (64%) represented putative upregulated miRNA targets, while only 119 out of 296 downregulated genes (40%) represented putative downregulated miRNA targets. These results suggest that the function of the alcohol-induced miRNAs is to target alcohol-induced genes.

### WGCNA identifies brain gene modules perturbed by ethanol

**Figure 4:**
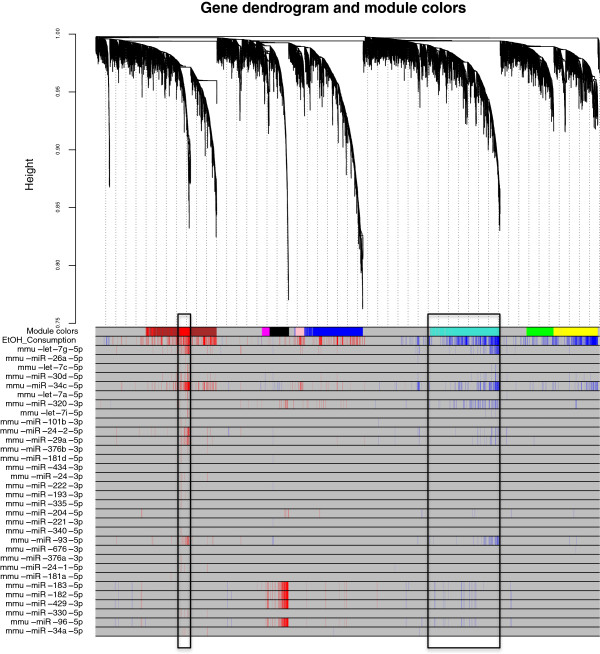
**Gene dendrogram, module assignment, and correlation to individual traits of interest.** The network was created from the weighted correlation matrix generated by WGCNA, by first calculating the adjacency matrix and then calculating topological overlap (TO) to hierarchically cluster genes into coexpression modules (see Materials and Methods). Final module assignments were made based on module membership. (Upper) Cluster dendrogram groups genes into distinct modules. The y-axis represents a dissimilarity distance (1 - TO). Dynamic tree cutting was used to determine modules, by dividing the dendrogram at significant branch points. (Lower) Correlation between individual genes and traits of interest (ethanol consumption and miRNA expression) with FDR < 0.10 are shown as color coded lines: red line indicates positive correlation and blue line indicates negative correlation). Red and turquoise modules (encased by rectangles) appear preferentially targeted by differentially expressed miRNAs.

To determine the relevant alcohol-responsive pathways regulated by miRNAs, we investigated the organization of the transcriptome in the frontal cortex of the mouse brain by applying weighted gene coexpression network analysis to the 32 samples as described in Materials and Methods. The weighted network identified groups (modules) of genes with similar expression patterns and high topological overlap. Topological overlap is a pairwise measurement that describes the similarity of two genes’ coexpression relationships with all other genes in the network [16,17] and has been shown to globally identify, for example, coexpression patterns that correlate with validated protein interactions in the human brain [18]. Our WGCNA identified 10 mRNA modules (Figure 4) with highly significant coexpression patterns, which we graphically corroborated by plotting the expression levels for the top 10 genes with the strongest membership on alcohol-related modules (Additional file 1: Figure S3). All modules were further correlated using the module eigengenes, with the phenotypic traits of interest, namely average ethanol consumption per voluntary drinking session and expression pattern of differentially expressed miRNAs. Table 2 summarizes the WGCNA module detection results. We included correlation values to the ethanol consumption trait and the associated P value of these correlations, as well as results from a variety of enrichment analyses. Additional details for enrichment analyses are provided in Additional file 1: Tables S4-S7). We want to point out that the number of modules produced by our WGCNA strategy seems rather low and the number of unassigned genes (grey module) rather large. One of the reasons for this behavior is that we constrained the minimum number of genes per module to a relatively high number (100 genes) in an effort to generate larger modules that improve detection of functional enrichment after gene enrichment analysis. Moreover, we purposely chose a low cutting height of 0.99 (default in WGCNA R package is 0.995) while implementing the dynamic tree cut method of the blockwiseModules function, to increase the stringency of the module detection step. Cutting height sets a threshold for dissimilarity between expression patterns to be considered for module detection: the lower the threshold, the more similar the patterns need to be for assignment into modules. Yet another potentially influential factor is the relatively small difference in gene expression between control and ethanol-treated animals, which may make it more difficult to detect significantly different patterns at stringent levels of statistical significance. The relatively small changes in gene and miRNA expression observed in rodent and human models of alcohol consumption are a common phenomenon described by researchers in the field [11,13,19].

**Table 2:** Modular enrichment analysis

**Gene module**	**Module correlation to EtOH consumption**	**P value of correlation to consumption**	**Differentially expressed (DE) genes in module**	**Module size**	**P value of enrichment in DE genes**	**Functional category enrichment**	**Cell type-specific enrichment**
**Red**	**0.63**	**1.08E-04**	**74**	**307**	**5.02E-16**	**Vesicle-mediated transport, Endocytosis**	**Oligodendrocytes**
**Brown**	**0.55**	**1.23E-03**	**88**	**846**	**6.72E-02**	**Transport, Synaptic transmission, Toll-like receptor & chemokine signaling**	**Neurons, Astrocytes**
**Pink**	**0.47**	**6.28E-03**	**30**	**150**	**1.89E-05**	**Cellular component organization or biogenesis, endosomal transport**	**-**
**Blue**	**0.35**	**4.77E-02**	**104**	**936**	**9.16E-03**	**Synaptic transmission, Neurotransmitter secretion**	**Neurons, Astrocytes, Oligodendrocytes**
Black	0.13	0.47	1	306	1	Regulation of developmental process, Neurogenesis	Astrocytes, Neurons, Oligodendrocytes
Grey	0.04	0.85	15	3339	1	-	Neurons, Oligodendrocytes
Magenta	-0.08	0.65	0	130	1	Blood vessel development, Angiogenesis, BMP signaling	Astrocytes, Microglia
Green	-0.40	2.44E-02	10	445	1	Oxidative phosphorylation, Ribosome biogenesis	Astrocytes
**Turquoise**	**-0.63**	**1.08E-04**	**224**	**1125**	**9.85E-36**	**Synaptic transmission, Chemokine signaling, Toll-like receptor signaling**	**Neurons, Astrocytes**
**Yellow**	**-0.74**	**1.35E-06**	**196**	**719**	**1.90E-52**	**Electron transport chain, Translation, Ribosome biogenesis**	**-**

Enrichment for differentially expressed genes, functional categories, and cell type-specific markers in coexpressed gene modules detected by WGCNA. Relevant modules are highlighted in bold fonts.

**Figure 5:**
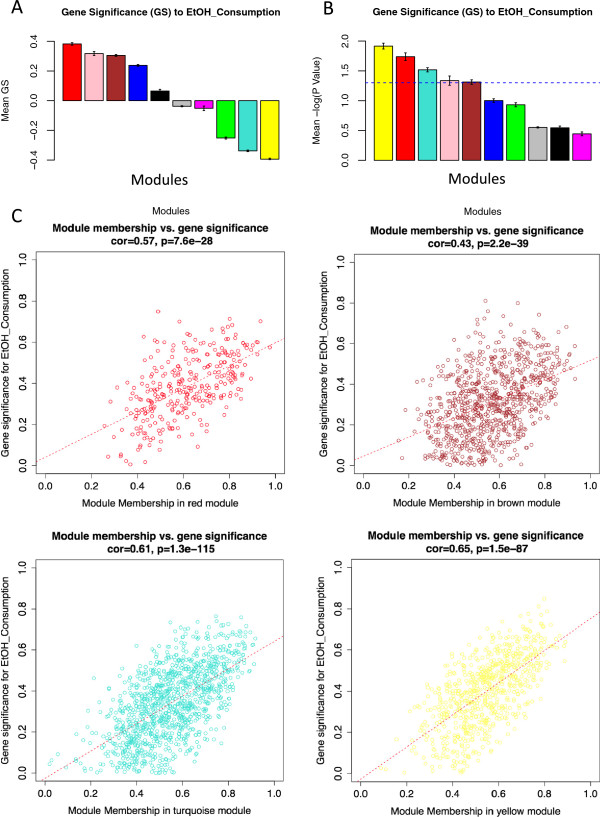
**Identifying alcohol-relevant modules by average gene significance and module membership. A**: Bar plot of the average gene significance for each detected module, equivalent to the average correlation among module genes and the ethanol consumption trait; **B**: Bar plot of the average -log P value of the gene significance; **C**: Plot of correlations between gene significance (GS) and module membership (MM) for representative alcohol-related modules. Color-coding is equivalent to module names. * Five modules (yellow, red, turquoise, pink, and brown) have an average P < 0.05 [-lg(P value) > 1.3].

**Figure 6:**
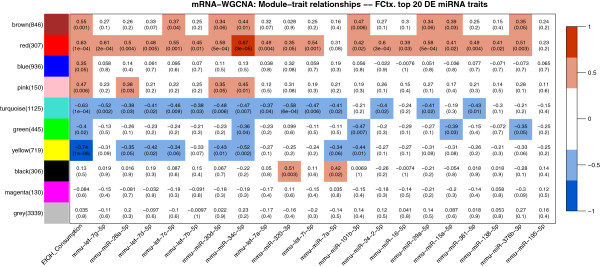
**Correlations of module eigengenes vs. consumption and top 20 differentially expressed miRNA traits.** Significant miRNA targeting is evident primarily against red and turquoise modules. Brown and yellow modules show significant correlations to a lower but relevant number of differentially expressed miRNAs.

Five modules (yellow, red, turquoise, pink, and brown) presented significant average correlations with the ethanol-related traits (Table 2, P < 0.01; Figure 5A and 5B; P < 0.05), thus warranting further investigation. The alcohol-responsive modules also presented highly significant correlations between module membership (MM) and gene significance (GS) to the ethanol-consumption trait (Figure 5C), which strengthen the inference of biological functions of the alcohol-significant genes by analyzing ontology category enrichment in the respective modules. Moreover, the five modules significantly correlated with ethanol drinking were significantly enriched with differentially expressed genes (Table 2). We also detected significant correlations (FDR < 0.1) between individual gene expression patterns and individual differentially expressed miRNA expression patterns (Figure 4, Additional file 1: Table S8), which provide indirect experimental validation for a group of predicted miRNA-mRNA interactions. By correlating the module eigengenes with the differentially expressed miRNA expression patterns (Figure 6) and considering the individual significant correlations shown in Figure 4, three modules (red and brown –positively correlated with ethanol drinking, and turquoise –negatively correlated with ethanol drinking), were found to be preferentially targeted by the differentially expressed miRNAs. To assess the validity of this approach, we corroborated the positively correlated expression levels of interacting miRNAs and mRNAs (i.e., mmu-let-7 vs. Syt11 and mmu-let-7 vs. Tom1) by qPCR. Additional confirmation for WGCNA-suggested interactions was obtained from TarBase, the database for validated interactions, where we found validated interactions between miRNAs and mRNAs that were positively correlated in our study but negatively correlated in previous publications: mmu-miR-34a-5p --| ACTB, mmu-miR-200b-3p --| ZEB2, mmu-miR-30a-5p --| TNRC6A, mmu-miR-152-3p --| CAMK2A, mmu-miR-200c-3p --| FLT1, mmu-miR-20a-5p --| ZBTB7A. Also, we found evidence in TarBase for validated interactions between miRNAs and mRNAs that were negatively correlated in our study as well as in previous publications: mmu-miR-29b-5p --| COL4A2 and mmu-miR-30a-5p5p --| AK1. Apparent spurious correlation was observed among some differentially expressed miRNAs and the black module. However, this module did not show significant correlation with the alcohol consumption phenotype. Hence the correlations detected among the black module and differentially expressed miRNAs are understood as pleiotropic interactions, akin to off-target effects (in this case referring to the miRNA targeting of non-alcohol-relevant transcripts).

A major theme emerging from these analyses is that the alcohol-responsive modules are enriched in genes involved in synaptic transmission and cytokine signaling pathways (Table 2, Additional file 1: Table S5-S7). This underscores the importance of these pathways in the transduction of alcohol actions. These modules showed enrichment for cell-type specific genes present in neurons, astrocytes, and oligodendrocytes, but not in microglia-specific genes, which highlight a major effect of alcohol on cells of the neuronal and macroglial lineages. These lineages share a common origin in precursor cells derived from the embryonic germ layer known as the neuroectoderm, which differ from the hematopoietic origin of microglia [20].

To assess potential network topology changes impinged by ethanol consumption, we conducted an alternative network analysis where we implemented WGCNA on each animal group independently. Calculation of the overlap between coexpressed gene modules from both networks identified significant differences in the modular organization of the independent networks (Additional file 1: Figure S4). Panel C in Additional file 1: Figure S4 reflects the reorganization of specific modules as the network “evolves” from a non-treated stage (network from control group) into an ethanol-treated stage (network from ethanol-treated group). For example, the control C_turquoise module diverted into two distinct modules in the alcohol network (A_brown and A_green). In addition, the alcohol (A_green) module included genes that had originally segregated into three distinct modules in the control network (C_turquoise, C greenyellow, and C_green). Our miRNA-mRNA integrative analysis suggests that such gene network reorganization events could be mediated, at least in part, by the activation of alcohol-responsive miRNAs.

### Core of interactions in ethanol-relevant, miRNA-targeted network modules highlights important regulators of alcohol action (hub genes)

**Figure 7:**
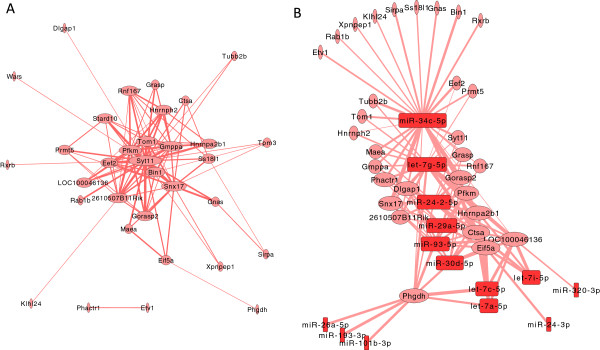
**Coexpression networks for red module. A**: coexpression network of gene-gene interactions among genes with high GS and high MM. Node width is proportional to node connectivity (number of edges/interacting partners), and edge size is proportional to the weight of the particular interaction; **B**: network of correlated miRNA-mRNA expression profiles (node width is proportional to the connectivity of the node, and edge size is proportional to the correlation between miRNA and mRNA expression). Pink ovals represent upregulated genes; red rectangles represents upregulated miRNAs.

To gain insight into the alcohol-responsive gene modules preferentially targeted by alcohol-induced miRNAs, we constructed gene interaction networks based on WGCNA correlation measurements for the red, brown, and turquoise modules. In the red module containing alcohol-upregulated genes, synaptotagmin-11 (Syt11), a member of the synaptotagmin gene family, which is involved in vesicle trafficking and synaptic transmission, is at the centre of the interaction network and the hub gene with higher connectivity (Figure 7A). Several other genes in the red module, such as sorting nexin-17 (Snx17), target of myb1 (Tom1), and bridging integrator 1 (Bin1), are also involved in membrane trafficking and sorting processes relevant to synaptic transmission as well as the recycling of adaptor components involved in signaling of Toll receptors and interleukin IL-1 receptor, among others [21-24]. Regarding miRNA targeting of these hub genes (Figure 7B), miR-34c-5p and let-7g-5p are main regulatory candidates based on significant expression pattern correlations (FDR < 0.10) with Syt11, Snx-7, Tom1 (both let-7g-5p and miR-34c-5p), and Bim1 (miR-34c-5p). This suggests that miR-34c-5p and let-7g-5p play an important role in the attempted regulation of this module. The central role played by miR-34c-5p and let-7g-5p while targeting additional genes in the red module is also evident in the expression correlation network shown in Additional file 1: Figure S4 and S5 (note the higher connectivity of these particular miRNAs).

In the brown module, which also contains alcohol-upregulated genes, protein phosphatase Mg(2+)/Mn(2+)-dependent 1A (Ppm1a) and heat shock 70kDa protein 8 (Hspa8) are the main hub genes. In addition, another heat shock protein (Hsp90ab1), GDP dissociation inhibitor 1 (Gdi1), and the glycine receptor beta (Glrb) (Additional file 1: Figure S5A) were among the highly interconnected genes in the module. The MAPK signaling pathway was the most significantly enriched pathway in this module (including genes Hspa8, Ppm1a, Ppp3ca, Ppp5c, Fgf13, Rasgrf1, Akt3, Mapk1, Mapk9; FDR = 1.2 × 10^-3^ Additional file 1: Table S6). MicroRNA miR-34c-5p appeared again as a main modulator of hub genes in this module, namely Ppm1a and Hsp90ab1, as detected by significant correlation among respective miRNA and mRNA expression profiles. The central role of miR-34c-5p while targeting additional genes in the brown module is again evident in the expression correlation network shown in Additional file 1: Figure S5B (note the higher connectivity of this particular miRNA).

In the turquoise module (Additional file 1: Figure S6A) containing alcohol-downregulated genes, we detected a larger core of hub genes known to be enriched in synaptic transmission, endocytosis, and cytokine signaling (Additional file 1: Table S7). The CXCR4-mediated signaling pathway appeared as one of the most enriched. Concordantly, the CXCR4 gene was previously reported as one of the genes more significantly overtargeted by miRNAs in PFC of human alcoholics [11]. Regarding miRNA regulation of this module, the central role of miR-34c-5p and let-7g-5p is yet again evident in the expression correlation network shown in Additional file 1: Figure S6B (note the higher connectivity of these particular miRNAs).

## Discussion

The goal of this study was to gain insights into the molecular mechanisms that are induced by alcohol consumption and governed by miRNA regulation in an early stage of development of dependence. This would allow for better understanding of how molecular progression of the disease occurs. Using a well-characterized mouse model developed in our lab [25,26], we evaluated whole transcriptome profiles of miRNA and mRNA expression from the same samples (“paired profiles”).

We found that a significant number of miRNAs that are upregulated in the FCtx of ethanol-treated mice are also upregulated in the PFC of human alcoholics [4,11]. Similarly, we found that many genes are differentially expressed in the FCtx of the treated animals, and a significant number of these are also differentially expressed in PFC of human alcoholics [14,15]. Interestingly, about 40-80% of the common differentially expressed genes change in opposite directions when the human and mouse models are compared, with a majority of differentially expressed genes being upregulated in the ethanol-treated mouse brains but downregulated in the brains of alcoholics. Our results demonstrate first, a high degree of conservation of alcohol-relevant genes and pathways from mouse to human and second, unique differences in the direction of gene expression changes between the two models. Such distinctions may be a consequence of the duration of ethanol exposure, which lasted for most of the adult life in the case of the human alcoholics (long term adaptations), but only 20 days during early adulthood in the case of our treated mice (early stages of adaptation). These differences suggest a dynamic gene regulatory network that changes as subjects advance towards alcohol dependence. We acknowledge that there may be undetermined effects that influence our comparison of results across the two species: 1) our expression analysis in mice represents a snapshot at 24 hours after the drinking protocol was completed; therefore, the animals could have been experiencing acute withdrawal; while 2) the post-mortem human brain samples originated from human alcoholics that were considered active heavy drinkers until the time of death, thus negating effects of acute withdrawal in this group. It would be beneficial to conduct additional expression studies after longer drinking protocols to determine a time sequence of expression changes in the alcohol-treated mouse as it progresses towards long-term ethanol consumption. It is also important to conduct expression profiling after longer time points of ethanol withdrawal, to determine whether withdrawal may influence the direction of mRNA and miRNA expression changes in the mouse.

The mouse miRNA-mRNA interaction networks presented here provide evidence for some striking features in the alcohol-specific network topologies. Contrary to expectations and to what happens in the brain of chronic human alcoholics, we did not find differences between the negatively correlated interaction network (upregulated miRNAs targeting downregulated mRNAs) and a random network, which indicates no selective pressure under the alcohol treatment for this type of interaction. Interestingly, we discovered that the positively correlated network (upregulated miRNAs targeting upregulated mRNAs) displayed an unexpected behavior, being twice as interconnected than expected by chance, and with a highly dense core of interactions. When overrepresentation of miRNA targets was evaluated, we found that a majority of upregulated miRNAs (over 80%) have a significantly higher number of upregulated targets than is expected by chance (enrichment for over-targeting), while only 24% of the miRNAs displayed the behavior towards downregulated genes (depletion for over-targeting). We speculate that miRNA targeting of upregulated genes might be in an “uncompensated" state in our mouse model, where miRNAs have not yet been able to downregulate the expression of their relevant targets. The extended and highly statistically significant over-targeting of upregulated genes in general, might indicate a signal for transcript downregulation. We speculate that a selective pressure to target ethanol-induced (upregulated) genes is impinged by chronic, binge alcohol consumption in an apparent adaptive response from the cellular environment to recover homeostasis. To our knowledge, this is the first report identifying positively correlated miRNA-mRNA networks in an animal model, which we propose as an adaptive mechanism to reinstate cellular homeostasis. Our current working model suggests that alcohol drinking activates a variety of brain genes and pathways that are consequently targeted for down-regulation by mature miRNAs that are activated in response to the aforementioned gene activation. Over-targeting of red and brown module genes by several miRNAs described in this work are examples of the behavior. Our assumption is that activated miRNAs in the mouse frontal cortex had not yet been able to overcome the induced upregulation of alcohol-responsive genes, at the experimental time point chosen to collect the mouse brains and conduct the expression profiling studies. We also suggest, based on expression patterns in similar brain regions from human alcoholics, that miRNAs eventually dominate the regulatory interactions with targeted genes and consequently induce a downregulated state in those alcohol-responsive gene.

These results underscore the biological importance of targeting alcohol-induced genes by alcohol-induced miRNAs and suggest that induction of miRNA targeting in the frontal cortex is a conserved (from rodent to human), adaptive response to counter an initial activation of transcription induced by high ethanol consumption. One possible explanation for the unexpected correlation (between upregulated miRNAs and upregulated target mRNAs) in the early stages of adaptation (binge mouse model) is the temporal inability of miRNA expression levels to cope with the levels of expression of target mRNAs. It is unlikely that indirect activities (e.g., activation of certain ethanol-responsive transcription factors that concomitantly activate certain miRNAs and respective target genes) are responsible for such over-targeting of upregulated mRNAs by a majority of the upregulated miRNAs.

The coexpression network analysis underscores the main biological processes affected by alcohol and miRNA regulation in the mouse model, such as synaptic vesicle-mediated transport and endocytic recycling, as well as neuroimmune signaling mediated by chemokine and Toll-like receptor signaling. The fact that signaling pathways that are upregulated by alcohol and over-targeted by upregulated miRNAs also show up enriched in downregulated modules, confirms the importance of these pathways in transducing alcohol’s actions during early stages of chronic alcohol consumption and their role as triggers of a miRNA adaptive response aimed at counteracting such activation. MicroRNAs miR-34c-5p and let-7g-5p, in particular, appear to be central regulators of hub genes in the most significant ethanol-responsive gene modules. Similarly, these two miRNAs were also upregulated in PFC of human alcoholics [4,11], and a let-7 family member was demonstrated to alter cocaine-induced conditioned place preference behavior when manipulated in the nucleus accumbens of rats [27]. We are enthusiastic about future prospects of using let-7 modulation in mouse brain to induce behavioural changes related to alcohol consumption and development of dependence.

One of the miRNA-targeted hub genes highlighted by the coexpression network analysis was synaptotagmin 11 (red module). The synaptotagmin family members are known to function by cooperating with SNARE proteins and accelerating membrane fusion [28,29]. Several other genes in the red module, such as sorting nexin-17 (Snx17), target of myb1 (Tom1), and bridging integrator 1 (Bin1), are also involved in membrane trafficking and sorting processes relevant to synaptic transmission as well as the recycling of adaptor components involved in signaling of Toll receptors and the interleukin IL-1 receptor [21-24,30]. These results underscore the relevance of synaptic vesicle transport and endocytic recycling in the transduction of alcohol’s actions. Our group has previously gathered proteomics/genomics evidence that support the role of these pathways [31-33].

Among the hub genes in the brown module, Ppm1a is strongly expressed in the brain and involved in the regulation of the stress response by acting on mitogen-activated protein (MAP) kinase signaling and by regulating the immune response [34,35]. Ethanol is well known to differentially modulate MAP kinase signaling cascades depending on the cell type, brain region, and ethanol treatment paradigm [3,36-42]. PPM1A phosphatase has been reported to play an important role in the termination of TNFα-mediated NFκB activation through dephosphorylation and inactivation of IKKβ [35]. Accordingly, the NF-κB pathway has been widely implicated in the transduction of alcohol effects, and activation of the pathway is associated with increased ethanol consumption [43-49]. Stress-induced chaperones Hspa8 (an Hsp70 family member) and Hsp90ab were also among brown module’s highly connected genes. These chaperons exert a variety of cellular functions including proteostasis maintenance [50,51] and endotoxin-like effects through Toll-like receptor 4 (TLR4) [52,53]. Upregulation of genes encoding several classes of chaperons has been previously reported after ethanol treatment [54-56]. Hsp70 and Hsp90 have also been reported as co-clustering factors of the LPS-sensing complex, which also contains CD14, CXCR4, GDF5, and TLR4 [57]. Importantly, LPS-triggered signaling through this complex has been recently reported to increase ethanol consumption and to alter certain aspects of alcohol reward and aversion in mice [46]. Glrb, another hub gene in the brown module, codes for the beta subunit of the glycine receptor (a neurotransmitter-gated pentameric ion channel) that is essential for targeting the receptor to the synapse. Glycine receptors contain an integral chloride channel and are known to have modulatory sites for anesthetics, neurosteroids, inhaled drugs of abuse, and ethanol [58-61]. Interestingly, α3 subunit-containing glycine receptors expressed in spinal cord dorsal horn synapses have been found to be specifically inhibited by inflammatory mediators [62,63], which raises the possibility that ethanol and ethanol-induced inflammatory signals may initiate converging activities through glycine receptor signaling in the forebrain. Overall, this module emphasizes the primary role that activation of stress and inflammatory responses, as well as intracellular vesicle transport and neurotransmitter-mediated signaling, play in the establishment of ethanol-induced behaviors.

In the turquoise module, the CXCR4-mediated signaling pathway appeared as one of the most enriched. Concordantly, the CXCR4 gene was previously reported as one of the genes more significantly overtargeted by miRNAs in PFC of human alcoholics [11]. This result underscores the relevance of this cytokine receptor pathway in the transduction of alcohol actions. CXCR4 has been reported to interact with the LPS-sensing complex that also includes CD14, Hsp70, Hsp90, GDF5, and TLR4 [57]. As mentioned earlier, LPS-triggered signaling through this complex has been recently reported to increase ethanol consumption and to alter certain aspects of alcohol reward and aversion in mice [46]. CXCR4 has also been implicated in opiate-induced hypernociception [64] and polymorphisms in SDF1 (aka CXCL12, the CXCR4 ligand) associated with several phenotypes, including alcohol consumption [65]. In addition, CXCR4 has been implicated in glutamate exocytosis from astrocytes [66], differentiation of oligodendrocytes progenitors and remyelination [67], and appears to be required for proper distribution of GABAergic interneurons and the establishment of functional cortical circuitry in certain cortical regions and layers [68]. Moreover, the CXCR4 ligand (CXCL12) induced presynaptic enhancement of GABA and glutamate release at serotonin dorsal raphe nucleus neurons [69].

The evidence shown here suggests that one of alcohol’s primary effects, in the early stages of development of dependence (represented here by the binge-drinking mouse model), is the transcriptional activation of genes involved in synaptic signaling, endocytic transport, and inflammatory response. A limitation of our study is the temporal resolution of the gene expression signal, which is a snapshot at the end of the drinking protocol. We speculate that our results describe the “intermediate state” of a dynamic process of mRNA regulation by miRNAs. Future studies need to explore gene expression profiles at additional time points during the drinking protocol and for an extended period of time and validate the regulatory relationship between miRNA/mRNA pairs. Based on our current model, we speculate that with time, the brain under ethanol insult mounts an adaptive miRNA response that counteracts the effect of the initial transcriptional activation. The well-adapted human alcoholic brain might represent this latter state. This hypothesis is consistent with processes of adaptive homeostasis generally triggered by addiction disorders [44,70,71]. The level of mechanistic conservation we observe between the mouse and human models is impressive, thereby warranting further investigation of the regulatory networks identified in our mouse model.

## Conclusions

By simultaneously studying miRNA and mRNA expression profiles in the frontal cortex of ethanol-treated mice and comparing these with similar studies conducted in brains of human alcoholics, we provide evidence that alcohol drinking induces extensive activation of miRNA expression. We suggest that this is an adaptive response to modulate an initial broad activation of genes primarily involved in synaptic signaling, endocytic transport, and inflammatory response. Importantly, conserved subsets of miRNAs and mRNAs demonstrate changing expression levels in response to alcohol in both mice and humans. Our results provide the first evidence for positively correlated miRNA-mRNA interaction networks based on expression correlation analyses that suggest an adaptive miRNA response secondary to the activation of gene expression in brains under ethanol insult. This study underscores the value of integrative miRNA-mRNA analyses that could be critical for the future of addiction research. Further analysis should be conducted with independent expression profiling datasets to validate the use of these conserved miRNA and mRNA expression signatures as biomarkers for ethanol consumption. *In vivo* studies (i.e., manipulation of specific miRNA levels in mouse brain using direct stereotaxic injection in mutant mice) are currently in progress in our lab to elaborate on our findings and to discover specific miRNAs or relevant miRNA combinations that could alter drinking behaviors. Our results identify miRNAs as novel and promising therapeutic candidates for the treatment of alcoholism and other complex psychiatric disorders.

## Methods

### Mice and drinking protocol

Female hybrid F1 mice from reciprocal intercrosses of C57BL/6J x FVB/NJ F1 and FVB/NJ x C57BL/6J F1 (maternal strain x paternal strain) were used. These mice have been shown to drink the most ethanol at high concentrations and to achieve behaviorally significant blood ethanol concentrations [26]. The B6 and FVB breeders were purchased from The Jackson Laboratory (Bar Harbor, ME) and mated at age 8 weeks in the Texas Genetic Animal Core of the INIA (Integrated Neuroscience Initiative on Alcohol) at the University of Texas at Austin. General methods implemented were as previously described [26]. Alcohol drinking protocol followed a Two-Bottle Choice (2BC)-Drinking in the Dark (DID) paradigm. In this paradigm, mice were allowed to voluntarily drink 20% ethanol or water from two separate bottles (bottle positions were alternated daily) during a 3-hour time span starting three hours into the dark cycle. This modification of the DID test was published by Blednov and Harris [72]. The drinking protocol was maintained for a total of 20 days and mice sacrificed by cervical dislocation 20 hours after the last drinking session. Brains were quickly removed and snap frozen in liquid nitrogen. Thirty-two mice were used in this study: 20 ethanol-treated and 12 matched controls. This study is in compliance with animal research guidelines and was approved by the Institutional Review Board of the University of Texas at Austin.

### Total RNA extraction from brain tissue and microarray expression profiling

Frontal cortex (FCtx) tissue was dissected using a brain slicer (Zivic Instruments, Pittsburg, PA) to produce a 2-mm coronal section from the most rostral portion of the mouse brain devoid of olfactory bulbs (coordinates Bregma +1.56 to +3.56). The dorsal part of this coronal section, cut immediately above the forceps minor of the corpus callosum as the anatomical landmark, was used for RNA extraction. This section of the cortex is mostly composed of frontal associated cortex (FrA), cingulate cortex area 1 (Cg1), prelimbic cortex (PrL), and primary (M1) and secondary (M2) motor cortices, as depicted in the mouse brain atlas [73]. Samples from both alcohol-treated and control groups were always included in each batch of extracted RNA. Total RNA was extracted using the mirVana® miRNA Isolation kit (Ambion, Austin, TX) according to the manufacturer’s instructions. Yield and quality of the total RNA preparation was determined using the Agilent 2100 Bioanalyzer (Agilent, Palo Alto, CA). For mRNA expression profiling, biotin-labeled cRNA was prepared using Illumina TotalPrep RNA Amplification kit (Ambion, Austin, TX) and then hybridized to Illumina MouseRef-8 v2.0 Expression BeadChips (Illumina, San Diego, CA). The quality of the Illumina bead summary data was assessed using the Bioconductor packages Lumi and arrayQualityMetrics. Data preprocessing included variance stabilization and quantile normalization using the Lumi package. Statistical analysis comparing ethanol-treated and control groups was performed using the Bioconductor package limma, which implements an empirical Bayes approach in R [74]. For miRNA expression profiling, Exiqon miRCURY LNA microRNA Arrays 5th generation (Exiqon, Vedbaek, Denmark) were used for hybridization and scanning at the Moffitt Cancer Center Microarray Facility (Tampa, FL). Data analysis was performed using the limma package. Data preprocessing included minimum background correction and scale normalization between arrays. As each probe was spotted as four replicates on the arrays, within-array replication was assessed using the limma duplicate correlation function. False discovery rate (FDR) was assessed using the Benjamini-Hochberg method [75]. Our list of differentially expressed genes was compared to the list of differentially expressed genes from human alcoholics reported previously [14], and also compared to the list of differentially expressed genes from a mouse model of alcohol dependence reported previously [15]. The statistical significance of the matches was empirically evaluated by implementing Monte Carlo simulations in the R environment (R Core Team 2012. R: A language and environment for statistical computing. R Foundation for Statistical Computing, Vienna, Austria. ISBN 3-900051-07-0, URL http://www.R-project.org/).

### Validation by real-time PCR analysis

For miRNA validation, single-stranded cDNA was synthesized from total RNA using the TaqMan® MiRNA Reverse Transcription kit (Applied Biosystems, Foster City, CA). Following reverse transcription, quantitative real time PCR (qPCR) was performed in triplicate, using TaqMan® MiRNA Assays together with the TaqMan® Universal PCR Master Mix (Applied Biosystems), as per manufacturer’s instructions. TaqMan® miRNA assays used were: mmu-miR-7a-5p (ID: 000268), mmu-miR-15b-5p (ID: 000390), mmu-miR-101a-3p (ID: 002253), mmu-miR-101b-3p (ID: 002531), mmu-miR-140-5p (ID: 001187), mmu-miR-152-3p (ID: 000475), mmu-miR-195a-5p (ID: 000494), mmu-miR-541-5p (ID: 002562), mmu-let-7c-5p (ID: 000379), mmu-let-7f-5p (ID: 000382), and mmu-let-7g-5p (ID: 002282). Assays used for endogenous control were: snoRNA142 (ID: 001231) and snoRNA234 (ID: 001234), which were chosen out of five endogenous control assays tested in our samples. Reactions were carried out in a 7900HT Fast Real-Time PCR System and data collected using SDS software (Applied Biosystems). For mRNA validation, single-stranded cDNA was synthesized from total RNA using the TaqMan® High Capacity cDNA Reverse Transcription Kit (Applied Biosystems). Following reverse transcription, qPCR was performed in triplicate, using TaqMan® Gene Expression Assays together with the TaqMan® Universal PCR Master Mix (Applied Biosystems), as per manufacturer’s instructions. TaqMan® Gene expression assays used were: Syt11 (ID: Mm00444517_m1), Atp2b1(ID: Mm01245805_m1), and Fermt2 (ID: Mm00600590_m1). Assays Hprt (ID: Mm00446968_m1) and Gusb (ID: Mm00446953_m1) were used as endogenous controls after being selected as the most invariable control assays tested in our samples. GenEx software (MultiD Analyses AB, Gothenburg, Sweden) was used for analysis of real-time PCR data, including selection of endogenous controls using the GeNorm [76] and NormFinder [77] algorithms.

### Weighted Gene Coexpression Network Analysis (WGCNA)

WGCNA was conducted as described previously [15] with modifications of a few parameters mentioned below. The general framework of WGCNA has been described in detail elsewhere [17]. In short, we constructed a signed network by calculating the Pearson’s correlations for all pairs of genes and the signed similarity (Sij) matrix derived from S_ij_ = (1 + cor(x_i_,x_j_))/2, where gene expression profiles x_i_ and x_j_ consist of the expression of genes i and j across multiple microarray samples. In the signed network, the direction of the changes in expression profiles can be inferred. The signed similarity (S_ij_) was then raised to the power β to represent the connection strength (a_ij_): a_ij_ = S_ij_^β^. This step aims to emphasize strong correlations and reduce the emphasis of weak correlations on an exponential scale [15]. We chose a power of β = 12 so that the resulting networks exhibited approximate scale-free topology (Soft.R.sq = 0.88). All genes were hierarchically clustered based on a dissimilarity measure of topological overlap which measures interconnectedness for a pair of genes [17]. The resulting gene dendrogram was used for module detection using the dynamic tree cut method and blockwiseModules function parameters: block size = 15000, minimum module size = 100, cutting height = 0.99, and deepSplit = 4. Gene modules corresponding to the branches cut off of the gene tree were labeled in unique colors. Unassigned genes were assigned to the grey module. Interaction networks were constructed for select modules. Only genes with high module membership (MM > 0.5) and high gene significance (GS > 0.5) were included in these networks. The trait-based gene significance measure is defined in WGCNA as the absolute value of the correlation between a specific node (gene) profile and the sample trait (ethanol consumption). The higher the value of GS, the more biologically significant the specific gene. On the other hand, module membership, also known as eigengene-based connectivity, correlates the gene expression profile of the specific gene with the module eigengene of a given module. Highly connected intramodular hub genes tend to have high module membership values to the respective module [78]. The WGCNA analysis was first conducted on all mice (controls and alcohol-treated) and included all genes on the chips that were present (expressed over noise levels) in 75% or more of the samples. This first network is not unique to alcohol but represent an admixture of alcohol and control expression levels, which are later correlated with ethanol consumption to make inferences about the changes in expression being driven by ethanol consumption. A second analysis was conducted on each group independently in order to assess whether changes in network topology occurred between the coexpression network in control animals and the coexpression network in alcohol-treated animals.

### Construction of miRNA-mRNA interaction database

A universe of miRNA-mRNA interactions for all reported miRNAs was constructed as a composite of all validated interactions downloaded from TarBase (http://diana.cslab.ece.ntua.gr/tarbase) [79] and predicted targets reported by miRecords (http://miRecords.biolead.org) [80]. The predicted component integrates the predictions produced by 11 established miRNA target prediction programs (DIANA-microT, MicroInspector, miRanda, MirTarget2, miTarget, NBmiRTar, PicTar, PITA, RNA22, RNAhybrid, and TargetScan⁄TargertScanS). Predictions were filtered to only consider those targets predicted by at least 4 of 11 prediction algorithms. The TarBase validated interactions were then added to the filtered list and duplicated interactions eliminated (validated interactions were given preference) to avoid overcounting during overrepresentation analyses. miRecords’ predicted target information for every mouse miRNA accessible through the website was obtained using the RCurl package. MicroRNA family information was downloaded from miRBase (http://miRBase.org) [81] and included into the miRNA-mRNA interaction universe.

### Integrative analysis of miRNA and mRNA microarray data

Statistical tests for comparison of multiple experimental proportions extracted from the integrated data set containing information about differentially expressed miRNAs, differentially expressed mRNAs, and putative/validated miRNA targets were implemented in the R environment. Specifically, hypergeometric tests for each differentially expressed miRNA were conducted to determine whether corresponding targets were overrepresented among inversely and directly correlated differentially expressed mRNAs from the same samples. Hypergeometric tests for each differentially expressed mRNA that is predicted to be targeted by more than one miRNA were conducted to determine whether any of such mRNAs could be subjected to “over-targeting” by miRNAs (an indication of redundant regulation or potential combinatorial action of miRNAs). When comparisons were made between differentially expressed sets of miRNAs from distinct species (i.e., mouse vs. human and mouse vs. rat) the species-specific miRNA IDs were converted to family IDs, which are consistent across species. MicroRNA families were downloaded from miRBase and ID conversion and matching code automated in R. When comparisons were implemented between differentially expressed sets of genes from distinct species (i.e., mouse vs. human), the human gene symbols were first converted into mouse symbols based on homology matching. The homology conversion table was downloaded from the Homologene database (http://ftp.ncbi.nih.gov/pub/HomoloGene/current/homologene.data) and ID conversion and matching code automated in R.

### Functional enrichment analyses

Enrichment analysis of gene ontology annotations were conducted using lists of differentially expressed mRNAs and module-specific mRNAs using ToppGene functional analysis tool and the R packages GO.db and GOstats. To determine which cell types could have contributed to the detected gene expression changes and coexpression patterns in each module, cell-type enrichment analysis was conducted as described by Ponomarev and collaborators [15]. Gene sets known to be preferentially expressed in mouse neurons, oligodendrocytes, astrocytes, and microglia were used. Statistical analysis was performed using the hypergeometric test and corrections for multiple comparisons using the Benjamini-Hochberg method.

## Availability of supporting data

The data discussed in this publication have been deposited in NCBI's Gene Expression Omnibus [82] and are accessible through GEO Series accession number GSE50427 (http://www.ncbi.nlm.nih.gov/geo/query/acc.cgi?acc=GSE50427).

## Competing interest

The authors declare that they have no competing interests.

## Authors’ contribution

YON planned and carried out experiments, implemented bioinformatic algorithms for data analysis, and drafted the manuscript. JMT, GG, and ONP planned and carried out experiments and contributed to data analysis. YAB planned and supervised the mouse alcohol consumption studies. RAH supervised experiments and critically reviewed manuscript for important intellectual content. RDM planned and supervised all experiments, analysed data, critically reviewed and gave final approval of the manuscript. All authors read and approved the final manuscript.

## Supplementary Material

Additional file 1: Figure S1 Random interaction networks generated as control networks. **Table S1.** Differentially expressed genes in FCtx of ethanol-treated mice. **Table S2.** Overrepresentation analysis of miRNA targets among upregulated genes. **Table S3.** Overrepresentation analysis of miRNA targets among downregulated genes. **Table S4.** Cell type-specific modular enrichment. **Table S5.** Functional enrichment analysis for red module genes with high GS and high MM. **Table S6.** Functional enrichment analysis for brown module genes with high GS and high MM. **Table S7.** Functional enrichment analysis for turquoise module genes with high GS and high MM. **Table S8.** Prediction of miRNA-mRNA interactions based on expression correlation patterns between single mRNAs and single differentially expressed (alcohol-responsive) miRNAs. Click here for file
