# Supplementary material for: Positively correlated miRNA-mRNA regulatory networks in mouse frontal cortex during early stages of alcohol dependence
Source: BMC Genomics. 2013 Oct 22;14:725. doi: 10.1186/1471-2164-14-725 (PMC3924350; doi:10.1186/1471-2164-14-725)
Supplement: Additional file 1: Figure S1 — Random interaction networks generated as control networks. Table S1. Differentially expressed genes in FCtx of ethanol-treated mice. Table S2. Overrepresentation analysis of miRNA targets among upregulated genes. Table S3. Overrepresentation analysis of miRNA targets among downregulated genes. Table S4. Cell type-specific modular enrichment. Table S5. Functional enrichment analysis for red module genes with high GS and high MM. Table S6. Functional enrichment analysis for brown module genes with high GS and high MM. Table S7. Functional enrichment analysis for turquoise module genes with high GS and high MM. Table S8. Prediction of miRNA-mRNA interactions based on expression correlation patterns between single mRNAs and single differentially expressed (alcohol-responsive) miRNAs. [file 1471-2164-14-725-S1.docx]

**Supplemental figures and tables**

A. B.

**Figure S1**

**Conserved differential gene expression in response to ethanol.** A: Venn diagram highlights common set of 29 differentially expressed genes in prefrontal cortex of human alcoholics, as reported by Liu et al. 2006, and ethanol-treated mice (this study); B: Venn diagram highlights common set of 84 differentially expressed genes in prefrontal cortex of human alcoholics, as reported by Ponomarev et al. 2012, and ethanol-treated mice (this study). P value empirically assessed after 100,000 Monte Carlo simulations.

1. Random network equivalent to negatively correlated network between upregulated miRNAs and downregulated mRNAs (average number of neighbors: 3.03)

1. Random network equivalent to positively correlated network between upregulated miRNAs and upregulated mRNAs (average number of neighbors: 2.52)

**Figure S2. Random interaction networks generated as control networks.** The average number of neighbors represents the average number of links (edges) a node has to other nodes. The size of the nodes is proportional to the number of edges (interactions) for each node.

**Figure S3**

**Expression levels for top 10 genes with strongest module membership in six alcohol-relevant modules.** Four modules (red, pink, brown, and blue) are upregulated and two modules (yellow and turquoise) are downregulated in alcohol-treated mice. X axis represents the individual samples. Commonly, the jump or fall of the expression line can be observed at the boundary between the two sample groups (between data points 12 & 13, identified by the doted line).

A. Control Group B. Alcohol Group

C. Module Overlap Network

**Supplementary Figure S4**

**Network topology chances detected in independent control and ethanol-treated coexpressed gene networks.** A: Dendrogram and coexpression modules generated by WGCNA. B: Cytoscape network representation (edge-weighted force-directed biolayout) of the statistically significant (P<0.01) modular overlaps between control (grey rectangles) and ethanol-treated (green rectangles) networks.

A. B.

**Figure S5**

**Coexpression networks for brown module.** A: coexpression network of gene-gene interactions among genes with high GS and high MM. Node width is proportional to node connectivity (number of edges/interacting partners), and edge size is proportional to the weight of the particular interaction; B: network of correlated miRNA-mRNA expression profiles (node width is proportional to the connectivity of the node, and edge size is proportional to the correlation between miRNA and mRNA expression). Pink ovals represent upregulated genes; red rectangles represents upregulated miRNAs.

A. B.

**Figure S6**

**Coexpression networks for turquoise module.** A: coexpression network of gene-gene interactions among genes with high GS and high MM. Node width is proportional to node connectivity (number of edges/interacting partners), and edge size is proportional to the weight of the particular interaction; B: network of correlated miRNA-mRNA expression profiles (node width is proportional to the connectivity of the node, and edge size is proportional to the correlation between miRNA and mRNA expression). Blue ovals represent downregulated genes; red rectangles represents upregulated miRNAs.

**Table S1. Differentially expressed genes in FCtx of ethanol-treated mice.**

| **Entrez ID** | **Symbol** | **logFC** | **Ave Expr** | **P.Value** | **adj.P.Val** |
| --- | --- | --- | --- | --- | --- |
| 54354 | Rassf5 | -0.29 | 9.18 | 3.50E-10 | 1.86E-06 |
| 67972 | Atp2b1 | 0.39 | 9.52 | 4.06E-10 | 1.86E-06 |
| 55978 | Ift20 | -0.25 | 10.22 | 4.88E-10 | 1.86E-06 |
| 78926 | Gas2l1 | -0.26 | 8.62 | 8.04E-10 | 2.30E-06 |
| 218952 | Fermt2 | 0.23 | 10.27 | 4.08E-09 | 9.32E-06 |
| 114641 | Rpl31 | -0.18 | 9.37 | 4.97E-09 | 9.47E-06 |
| 73710 | Tubb2b | 0.39 | 8.90 | 7.41E-09 | 1.06E-05 |
| 216767 | Mrpl22 | -0.27 | 9.62 | 1.82E-08 | 2.18E-05 |
| 68262 | Agpat4 | -0.28 | 9.43 | 1.90E-08 | 2.18E-05 |
| 56632 | Sphk2 | -0.24 | 8.82 | 2.48E-08 | 2.58E-05 |
| 110891 | Slc8a2 | -0.26 | 8.54 | 3.15E-08 | 2.86E-05 |
| 104001 | Rtn1 | -0.22 | 13.88 | 3.26E-08 | 2.86E-05 |
| 320563 | Islr2 | -0.60 | 8.96 | 5.73E-08 | 4.67E-05 |
| 14402 | Gabrb3 | 0.21 | 11.23 | 7.44E-08 | 5.67E-05 |
| 12349 | Car2 | 0.28 | 9.75 | 9.15E-08 | 6.54E-05 |
| 228662 | Btbd3 | 0.34 | 9.24 | 1.01E-07 | 6.82E-05 |
| 19299 | Abcd3 | 0.19 | 10.02 | 1.26E-07 | 7.88E-05 |
| 78653 | Bola3 | -0.24 | 9.70 | 1.33E-07 | 7.88E-05 |
| 11490 | Adam15 | -0.29 | 9.54 | 1.38E-07 | 7.88E-05 |
| 16825 | Ldb1 | 0.34 | 11.36 | 1.69E-07 | 8.23E-05 |
| 381677 | Vgf | -0.52 | 10.49 | 1.73E-07 | 8.23E-05 |
| 15199 | Hebp1 | -0.22 | 9.42 | 1.84E-07 | 8.40E-05 |
| 57740 | Stk32c | -0.29 | 8.61 | 2.27E-07 | 9.98E-05 |
| 20018 | Polr1d | -0.21 | 10.12 | 2.64E-07 | 1.11E-04 |
| 266781 | Snx17 | 0.29 | 8.88 | 2.71E-07 | 1.11E-04 |
| 14009 | Etv1 | 0.16 | 8.37 | 2.87E-07 | 1.13E-04 |
| 66148 | Dnajc15 | -0.26 | 10.20 | 3.81E-07 | 1.45E-04 |
| 22129 | Ttc3 | 0.18 | 13.43 | 4.71E-07 | 1.73E-04 |
| 218194 | Phactr1 | 0.46 | 9.20 | 4.84E-07 | 1.73E-04 |
| 100044294 | LOC100044294 | -0.24 | 10.83 | 5.19E-07 | 1.80E-04 |
| 27360 | Add3 | 0.26 | 10.51 | 5.99E-07 | 2.01E-04 |
| 68349 | Ndufs3 | -0.21 | 11.41 | 6.41E-07 | 2.05E-04 |
| 108013 | Brunol4 | 0.34 | 9.64 | 6.47E-07 | 2.05E-04 |
| 216169 | Fam108a | -0.27 | 11.75 | 6.77E-07 | 2.09E-04 |
| 69833 | Polr2f | -0.17 | 10.21 | 7.03E-07 | 2.11E-04 |
| 23827 | Bpnt1 | -0.23 | 8.66 | 7.22E-07 | 2.11E-04 |
| 212090 | Tmem60 | -0.23 | 10.23 | 7.48E-07 | 2.14E-04 |
| 216190 | Appl2 | 0.23 | 9.94 | 8.41E-07 | 2.35E-04 |
| 228410 | Cstf3 | -0.21 | 10.04 | 9.03E-07 | 2.35E-04 |
| 21681 | Thoc4 | -0.18 | 9.35 | 9.03E-07 | 2.35E-04 |
| 74277 | Chic2 | -0.19 | 9.15 | 9.04E-07 | 2.35E-04 |
| 11461 | Actb | 0.60 | 10.44 | 9.44E-07 | 2.40E-04 |
| 17748 | Mt1 | -0.26 | 13.95 | 1.02E-06 | 2.53E-04 |
| 13629 | Eef2 | 0.42 | 9.20 | 1.04E-06 | 2.53E-04 |
| 50529 | Mrps7 | -0.17 | 9.87 | 1.06E-06 | 2.53E-04 |
| 54616 | Extl3 | -0.31 | 9.99 | 1.12E-06 | 2.57E-04 |
| 26396 | Map2k2 | 0.27 | 10.83 | 1.12E-06 | 2.57E-04 |
| 66664 | Tmem41a | -0.23 | 9.78 | 1.27E-06 | 2.78E-04 |
| 78928 | Pigt | -0.33 | 8.73 | 1.29E-06 | 2.79E-04 |
| 11782 | Ap4s1 | -0.17 | 11.14 | 1.36E-06 | 2.89E-04 |
| 13046 | Cugbp1 | 0.31 | 8.71 | 1.42E-06 | 2.94E-04 |
| 13498 | Atn1 | -0.30 | 9.13 | 1.47E-06 | 3.01E-04 |
| 56219 | Extl1 | -0.25 | 9.46 | 1.59E-06 | 3.14E-04 |
| 74383 | Ubap2l | -0.16 | 8.61 | 1.62E-06 | 3.14E-04 |
| 64337 | Gng13 | -0.17 | 12.14 | 1.80E-06 | 3.42E-04 |
| 28080 | Atp5o | -0.23 | 9.44 | 1.83E-06 | 3.43E-04 |
| 76308 | Rab1b | 0.25 | 9.52 | 1.96E-06 | 3.49E-04 |
| 109145 | Gins4 | -0.20 | 9.68 | 1.97E-06 | 3.49E-04 |
| 20525 | Slc2a1 | -0.23 | 10.71 | 2.00E-06 | 3.49E-04 |
| 235431 | Coro2b | -0.29 | 9.59 | 2.01E-06 | 3.49E-04 |
| 117109 | Pop5 | -0.28 | 11.96 | 2.01E-06 | 3.49E-04 |
| 67916 | Ppap2b | 0.24 | 8.73 | 2.09E-06 | 3.49E-04 |
| 319604 | Fam168a | 0.49 | 9.77 | 2.10E-06 | 3.49E-04 |
| 18190 | Nrxn2 | -0.54 | 11.16 | 2.12E-06 | 3.49E-04 |
| 14086 | Fscn1 | -0.30 | 10.11 | 2.15E-06 | 3.49E-04 |
| 19171 | Psmb10 | -0.24 | 10.12 | 2.19E-06 | 3.49E-04 |
| 100046003 | LOC100046003 | -0.23 | 9.60 | 2.23E-06 | 3.49E-04 |
| 268480 | Rapgefl1 | -0.29 | 9.87 | 2.29E-06 | 3.54E-04 |
| 102462 | Imp3 | -0.22 | 10.60 | 2.38E-06 | 3.63E-04 |
| 54371 | Chst2 | -0.32 | 9.50 | 2.42E-06 | 3.63E-04 |
| 675228 | LOC675228 | -0.21 | 12.04 | 2.47E-06 | 3.63E-04 |
| 52915 | Zmiz2 | -0.29 | 10.28 | 2.74E-06 | 3.92E-04 |
| 229521 | Syt11 | 0.56 | 9.57 | 2.86E-06 | 4.04E-04 |
| 27050 | Rps3 | -0.25 | 10.82 | 3.07E-06 | 4.28E-04 |
| 19737 | Rgs5 | 0.22 | 8.68 | 3.10E-06 | 4.28E-04 |
| 71770 | Ap2b1 | -0.51 | 9.74 | 3.18E-06 | 4.32E-04 |
| 80909 | Gats | 0.24 | 9.76 | 3.30E-06 | 4.44E-04 |
| 11964 | Atp6v1a | 0.39 | 10.94 | 3.36E-06 | 4.46E-04 |
| 18626 | Per1 | -0.21 | 9.07 | 3.75E-06 | 4.93E-04 |
| 20779 | Src | -0.23 | 9.12 | 3.88E-06 | 4.98E-04 |
| 230775 | Bai2 | -0.34 | 10.95 | 4.00E-06 | 5.08E-04 |
| 223626 | 4930572J05Rik | -0.17 | 8.58 | 4.08E-06 | 5.12E-04 |
| 319517 | 6430510M02Rik | 0.36 | 10.83 | 4.21E-06 | 5.13E-04 |
| 54194 | Akap8l | 0.26 | 10.65 | 4.21E-06 | 5.13E-04 |
| 52822 | Rufy3 | 0.25 | 11.20 | 4.22E-06 | 5.13E-04 |
| 68796 | 1110039B18Rik | -0.17 | 8.85 | 4.31E-06 | 5.16E-04 |
| 69080 | Gmppa | 0.19 | 8.52 | 4.35E-06 | 5.16E-04 |
| 30948 | Bin1 | 0.17 | 8.50 | 4.40E-06 | 5.16E-04 |
| 229709 | Ahcyl1 | 0.33 | 11.37 | 4.54E-06 | 5.22E-04 |
| 242667 | Dlgap3 | -0.23 | 9.02 | 4.69E-06 | 5.25E-04 |
| 83603 | Elovl4 | -0.16 | 10.08 | 4.81E-06 | 5.31E-04 |
| 77065 | Ints7 | -0.16 | 8.89 | 4.83E-06 | 5.31E-04 |
| 67941 | Rps27l | -0.18 | 10.42 | 5.08E-06 | 5.44E-04 |
| 68581 | Tmed10 | -0.18 | 11.09 | 5.21E-06 | 5.49E-04 |
| 13527 | Dtna | 0.19 | 8.77 | 5.23E-06 | 5.49E-04 |
| 56282 | Mrpl12 | -0.27 | 10.64 | 5.30E-06 | 5.51E-04 |
| 27062 | Cadps | 0.18 | 11.45 | 5.41E-06 | 5.57E-04 |
| 232791 | Cnot3 | -0.16 | 8.72 | 5.59E-06 | 5.70E-04 |
| 13885 | Esd | -0.19 | 9.21 | 5.73E-06 | 5.80E-04 |
| 21968 | Tom1 | 0.22 | 8.60 | 5.98E-06 | 6.00E-04 |
| 277463 | Gpr107 | -0.20 | 8.85 | 6.06E-06 | 6.02E-04 |
| 94065 | Mrpl34 | -0.18 | 9.52 | 6.22E-06 | 6.13E-04 |
| 15572 | Elavl4 | 0.19 | 8.68 | 6.33E-06 | 6.16E-04 |
| 217869 | Eif5 | 0.27 | 12.00 | 6.37E-06 | 6.16E-04 |
| 100047659 | LOC100047659 | -0.29 | 10.07 | 6.41E-06 | 6.16E-04 |
| 57776 | Ttyh1 | 0.17 | 8.71 | 6.52E-06 | 6.17E-04 |
| 216881 | Wscd1 | -0.23 | 8.99 | 6.59E-06 | 6.17E-04 |
| 73046 | Glrx5 | -0.22 | 10.34 | 6.70E-06 | 6.17E-04 |
| 53951 | 2310002B06Rik | 0.15 | 8.69 | 6.74E-06 | 6.17E-04 |
| 22340 | Vegfb | -0.17 | 10.62 | 6.75E-06 | 6.17E-04 |
| 214952 | Rhot2 | -0.19 | 9.09 | 7.42E-06 | 6.63E-04 |
| 75292 | Prkd3 | 0.14 | 8.83 | 7.56E-06 | 6.67E-04 |
| 17751 | Mt3 | -0.24 | 12.13 | 7.59E-06 | 6.67E-04 |
| 27374 | Prmt5 | 0.16 | 8.42 | 7.96E-06 | 6.94E-04 |
| 74747 | Ddit4 | -0.25 | 9.22 | 8.06E-06 | 6.98E-04 |
| 18242 | Oat | 0.17 | 10.99 | 8.17E-06 | 7.02E-04 |
| 50918 | Myadm | -0.25 | 9.40 | 8.35E-06 | 7.12E-04 |
| 27973 | Vkorc1 | -0.18 | 9.40 | 8.54E-06 | 7.12E-04 |
| 59069 | Tpm3 | 0.16 | 8.81 | 8.55E-06 | 7.12E-04 |
| 20713 | Serpini1 | 0.28 | 10.57 | 8.59E-06 | 7.12E-04 |
| 26445 | Psmb2 | -0.21 | 10.92 | 8.89E-06 | 7.27E-04 |
| 108686 | Ccdc88a | 0.19 | 9.32 | 8.93E-06 | 7.27E-04 |
| 239650 | AI836003 | -0.20 | 8.95 | 9.31E-06 | 7.44E-04 |
| 15270 | H2afx | -0.15 | 9.06 | 9.45E-06 | 7.50E-04 |
| 21375 | Tbr1 | -0.29 | 9.77 | 9.52E-06 | 7.50E-04 |
| 13855 | Epn2 | 0.16 | 10.10 | 9.80E-06 | 7.62E-04 |
| 80913 | Pum2 | 0.22 | 9.08 | 1.02E-05 | 7.87E-04 |
| 270109 | Pcnxl2 | -0.27 | 9.95 | 1.04E-05 | 7.97E-04 |
| 101966 | D8Ertd738e | -0.17 | 10.45 | 1.05E-05 | 8.03E-04 |
| 76987 | Hdhd2 | 0.14 | 8.73 | 1.12E-05 | 8.35E-04 |
| 54130 | Actr1a | 0.17 | 11.16 | 1.14E-05 | 8.35E-04 |
| 74143 | Opa1 | 0.22 | 8.69 | 1.15E-05 | 8.35E-04 |
| 12827 | Col4a2 | -0.26 | 9.06 | 1.16E-05 | 8.35E-04 |
| 331063 | AI987692 | 0.15 | 8.33 | 1.17E-05 | 8.35E-04 |
| 75751 | Ipo4 | -0.21 | 9.35 | 1.18E-05 | 8.35E-04 |
| 19240 | Tmsb10 | -0.22 | 12.44 | 1.18E-05 | 8.35E-04 |
| 72018 | Fundc1 | 0.16 | 10.18 | 1.20E-05 | 8.35E-04 |
| 170750 | Xpnpep1 | 0.15 | 8.37 | 1.21E-05 | 8.35E-04 |
| 14790 | Grcc10 | -0.19 | 10.74 | 1.21E-05 | 8.35E-04 |
| 226162 | 5330431N19Rik | -0.23 | 10.76 | 1.23E-05 | 8.35E-04 |
| 59287 | Ncstn | -0.18 | 8.77 | 1.25E-05 | 8.35E-04 |
| 54399 | Bet1l | -0.18 | 9.74 | 1.26E-05 | 8.35E-04 |
| 66505 | Zmynd11 | 0.25 | 9.41 | 1.27E-05 | 8.35E-04 |
| 108083 | Pip4k2b | -0.24 | 10.80 | 1.27E-05 | 8.35E-04 |
| 246703 | Apoa1bp | -0.18 | 10.25 | 1.27E-05 | 8.35E-04 |
| 11775 | Ap3b2 | -0.20 | 10.44 | 1.27E-05 | 8.35E-04 |
| 11932 | Atp1b2 | -0.23 | 10.40 | 1.29E-05 | 8.35E-04 |
| 18751 | Prkcb | 0.24 | 13.16 | 1.30E-05 | 8.35E-04 |
| 22031 | Traf3 | -0.28 | 9.73 | 1.31E-05 | 8.35E-04 |
| 69568 | Vkorc1l1 | -0.16 | 8.94 | 1.32E-05 | 8.35E-04 |
| 67452 | Pnpla8 | 0.17 | 10.24 | 1.34E-05 | 8.45E-04 |
| 11816 | Apoe | 0.26 | 12.20 | 1.35E-05 | 8.47E-04 |
| 11927 | Atox1 | -0.21 | 10.14 | 1.47E-05 | 9.14E-04 |
| 27370 | Rps26 | -0.24 | 11.85 | 1.49E-05 | 9.18E-04 |
| 57783 | Tnip1 | -0.16 | 9.07 | 1.52E-05 | 9.34E-04 |
| 333331 | LOC333331 | 0.15 | 9.10 | 1.53E-05 | 9.36E-04 |
| 98682 | 2210010L05Rik | 0.15 | 8.52 | 1.60E-05 | 9.73E-04 |
| 20182 | Rxrb | 0.21 | 10.43 | 1.61E-05 | 9.73E-04 |
| 66119 | Tomm6 | -0.15 | 8.99 | 1.71E-05 | 1.02E-03 |
| 77980 | Sbf1 | 0.19 | 10.45 | 1.71E-05 | 1.02E-03 |
| 68617 | 1110012J17Rik | -0.18 | 9.94 | 1.73E-05 | 1.03E-03 |
| 75805 | Nln | 0.15 | 9.32 | 1.75E-05 | 1.03E-03 |
| 19346 | Rab6 | 0.35 | 11.01 | 1.78E-05 | 1.03E-03 |
| 67166 | Arl8b | 0.14 | 10.83 | 1.78E-05 | 1.03E-03 |
| 100637 | B230342M21Rik | 0.16 | 9.64 | 1.78E-05 | 1.03E-03 |
| 20338 | Sel1l | 0.26 | 9.65 | 1.79E-05 | 1.03E-03 |
| 21927 | Tnfaip1 | 0.17 | 10.72 | 1.84E-05 | 1.06E-03 |
| 56535 | Pex3 | 0.18 | 9.16 | 1.98E-05 | 1.13E-03 |
| 99045 | Mrps26 | -0.18 | 9.43 | 2.03E-05 | 1.15E-03 |
| 407785 | Ndufs6 | -0.18 | 12.47 | 2.09E-05 | 1.18E-03 |
| 26562 | Ncdn | -0.18 | 11.23 | 2.11E-05 | 1.18E-03 |
| 58799 | Crbn | 0.14 | 9.25 | 2.13E-05 | 1.18E-03 |
| 20196 | S100a13 | -0.17 | 8.94 | 2.13E-05 | 1.18E-03 |
| 57874 | Ptplad1 | -0.17 | 12.29 | 2.17E-05 | 1.19E-03 |
| 69202 | Ptms | -0.18 | 8.90 | 2.18E-05 | 1.19E-03 |
| 56350 | Arl3 | -0.15 | 10.57 | 2.19E-05 | 1.19E-03 |
| 69051 | Pycr2 | -0.21 | 9.40 | 2.22E-05 | 1.19E-03 |
| 74182 | Prei4 | 0.25 | 10.14 | 2.23E-05 | 1.19E-03 |
| 68936 | 1190017O12Rik | -0.19 | 9.71 | 2.25E-05 | 1.19E-03 |
| 16581 | Kifc2 | -0.22 | 11.65 | 2.26E-05 | 1.19E-03 |
| 72503 | 2610507B11Rik | 0.17 | 8.34 | 2.31E-05 | 1.21E-03 |
| 68634 | Tm2d3 | -0.16 | 9.21 | 2.34E-05 | 1.22E-03 |
| 17283 | Men1 | -0.22 | 9.28 | 2.51E-05 | 1.29E-03 |
| 18616 | Peg3 | 0.15 | 8.44 | 2.52E-05 | 1.29E-03 |
| 16661 | Krt10 | -0.22 | 9.36 | 2.55E-05 | 1.30E-03 |
| 60596 | Gucy1a3 | 0.22 | 8.95 | 2.67E-05 | 1.33E-03 |
| 18029 | Nfic | -0.17 | 8.95 | 2.73E-05 | 1.34E-03 |
| 57340 | Jph3 | -0.19 | 10.12 | 2.73E-05 | 1.34E-03 |
| 27366 | Txnl4a | -0.16 | 9.81 | 2.74E-05 | 1.34E-03 |
| 170658 | BC002163 | -0.18 | 11.19 | 2.81E-05 | 1.37E-03 |
| 69072 | Ebna1bp2 | -0.16 | 8.74 | 2.87E-05 | 1.40E-03 |
| 21761 | Morf4l1 | 0.21 | 12.44 | 2.97E-05 | 1.44E-03 |
| 19025 | Ctsa | 0.24 | 8.58 | 2.98E-05 | 1.44E-03 |
| 215690 | Nav1 | 0.26 | 10.50 | 3.01E-05 | 1.45E-03 |
| 100213 | Rusc2 | -0.18 | 10.52 | 3.10E-05 | 1.47E-03 |
| 53379 | Hnrnpa2b1 | 0.36 | 9.41 | 3.16E-05 | 1.48E-03 |
| 105638 | Dph3 | -0.18 | 9.38 | 3.26E-05 | 1.52E-03 |
| 328365 | Zmiz1 | 0.23 | 8.92 | 3.29E-05 | 1.53E-03 |
| 330814 | Lphn1 | 0.36 | 10.36 | 3.30E-05 | 1.53E-03 |
| 104010 | Cdh22 | -0.15 | 8.54 | 3.36E-05 | 1.55E-03 |
| 432763 | Prr7 | -0.18 | 8.91 | 3.44E-05 | 1.58E-03 |
| 224938 | Pja2 | 0.38 | 12.48 | 3.47E-05 | 1.58E-03 |
| 27176 | Rpl7a | -0.27 | 9.66 | 3.48E-05 | 1.58E-03 |
| 56491 | Vapb | -0.26 | 9.22 | 3.50E-05 | 1.59E-03 |
| 19177 | Psmb7 | -0.22 | 11.64 | 3.54E-05 | 1.59E-03 |
| 53598 | Dctn3 | -0.18 | 11.48 | 3.68E-05 | 1.63E-03 |
| 14683 | Gnas | 0.39 | 9.91 | 3.68E-05 | 1.63E-03 |
| 22278 | Usf1 | 0.14 | 8.31 | 3.69E-05 | 1.63E-03 |
| 69961 | 2810432D09Rik | -0.19 | 9.60 | 3.82E-05 | 1.67E-03 |
| 71310 | Tbc1d9 | -0.22 | 9.04 | 3.94E-05 | 1.71E-03 |
| 223978 | C530044N13Rik | 0.22 | 9.93 | 4.02E-05 | 1.73E-03 |
| 59003 | Maea | 0.25 | 8.92 | 4.08E-05 | 1.75E-03 |
| 217473 | Ankmy2 | 0.16 | 10.67 | 4.08E-05 | 1.75E-03 |
| 53380 | Psmd10 | -0.17 | 9.57 | 4.09E-05 | 1.75E-03 |
| 320713 | Mysm1 | 0.33 | 9.46 | 4.21E-05 | 1.78E-03 |
| 218544 | Sgtb | 0.33 | 9.57 | 4.23E-05 | 1.78E-03 |
| 66594 | Uqcr | -0.18 | 12.70 | 4.23E-05 | 1.78E-03 |
| 224997 | Dlgap1 | 0.16 | 8.40 | 4.25E-05 | 1.78E-03 |
| 21664 | Phlda1 | -0.30 | 9.41 | 4.27E-05 | 1.78E-03 |
| 226646 | Ndufs2 | 0.25 | 10.13 | 4.33E-05 | 1.79E-03 |
| 15460 | Hr | -0.18 | 9.45 | 4.35E-05 | 1.79E-03 |
| 246696 | Slc25a28 | 0.17 | 10.29 | 4.35E-05 | 1.79E-03 |
| 12540 | Cdc42 | 0.23 | 9.35 | 4.40E-05 | 1.80E-03 |
| 54709 | Eif3i | -0.17 | 10.51 | 4.49E-05 | 1.83E-03 |
| 14567 | Gdi1 | 0.39 | 10.36 | 4.50E-05 | 1.83E-03 |
| 235044 | BC018242 | -0.16 | 9.11 | 4.58E-05 | 1.85E-03 |
| 319670 | Eml5 | 0.17 | 8.81 | 4.61E-05 | 1.85E-03 |
| 76574 | Mfsd2 | -0.28 | 9.74 | 4.62E-05 | 1.85E-03 |
| 14702 | Gng2 | -0.21 | 9.09 | 4.65E-05 | 1.85E-03 |
| 276770 | Eif5a | 0.24 | 10.92 | 4.65E-05 | 1.85E-03 |
| 21916 | Tmod1 | 0.17 | 9.28 | 4.73E-05 | 1.86E-03 |
| 19417 | Rasgrf1 | 0.16 | 13.37 | 4.77E-05 | 1.88E-03 |
| 19894 | Rph3a | -0.26 | 10.03 | 4.80E-05 | 1.88E-03 |
| 24136 | Zeb2 | 0.26 | 9.24 | 5.10E-05 | 1.98E-03 |
| 94232 | Ubqln4 | -0.25 | 9.58 | 5.24E-05 | 2.02E-03 |
| 68033 | Cox19 | -0.18 | 9.79 | 5.26E-05 | 2.02E-03 |
| 67087 | Ctnnbip1 | -0.15 | 9.78 | 5.27E-05 | 2.02E-03 |
| 319749 | C230078M08Rik | -0.25 | 8.75 | 5.29E-05 | 2.02E-03 |
| 68404 | Nrn1 | -0.22 | 12.52 | 5.34E-05 | 2.04E-03 |
| 13131 | Dab1 | 0.16 | 8.79 | 5.58E-05 | 2.09E-03 |
| 224143 | Ktelc1 | 0.14 | 9.78 | 5.65E-05 | 2.11E-03 |
| 69773 | 1810026J23Rik | 0.18 | 9.60 | 5.84E-05 | 2.17E-03 |
| 76898 | B3gat1 | -0.20 | 11.71 | 5.92E-05 | 2.18E-03 |
| 217866 | Cdc42bpb | -0.21 | 10.29 | 5.98E-05 | 2.18E-03 |
| 83766 | Actl6b | -0.17 | 9.64 | 5.99E-05 | 2.18E-03 |
| 15516 | Hsp90ab1 | 0.41 | 10.31 | 6.01E-05 | 2.18E-03 |
| 105689 | Mycbp2 | 0.15 | 8.57 | 6.01E-05 | 2.18E-03 |
| 77862 | Thyn1 | -0.18 | 10.81 | 6.02E-05 | 2.18E-03 |
| 12729 | Clns1a | 0.17 | 8.77 | 6.10E-05 | 2.20E-03 |
| 100041294 | 100041294 | -0.18 | 11.60 | 6.11E-05 | 2.20E-03 |
| 232087 | Mat2a | 0.15 | 8.48 | 6.16E-05 | 2.21E-03 |
| 407819 | BC031181 | -0.15 | 10.81 | 6.22E-05 | 2.22E-03 |
| 11829 | Aqp4 | 0.42 | 9.40 | 6.23E-05 | 2.22E-03 |
| 55984 | Camkk1 | -0.25 | 9.69 | 6.26E-05 | 2.22E-03 |
| 76820 | D12Ertd553e | 0.17 | 11.53 | 6.37E-05 | 2.23E-03 |
| 13595 | Ebp | -0.17 | 9.05 | 6.39E-05 | 2.23E-03 |
| 56527 | Mast1 | -0.18 | 8.93 | 6.45E-05 | 2.25E-03 |
| 231070 | Insig1 | 0.18 | 8.52 | 6.54E-05 | 2.26E-03 |
| 106861 | Abhd3 | 0.17 | 9.11 | 6.56E-05 | 2.26E-03 |
| 56258 | Hnrnph2 | 0.15 | 8.36 | 6.82E-05 | 2.33E-03 |
| 20692 | Sparc | -0.28 | 9.77 | 6.82E-05 | 2.33E-03 |
| 13829 | Epb4.9 | -0.22 | 11.26 | 6.86E-05 | 2.33E-03 |
| 18810 | Plec1 | 0.19 | 11.22 | 7.03E-05 | 2.36E-03 |
| 83814 | Nedd4l | -0.17 | 9.75 | 7.05E-05 | 2.36E-03 |
| 15481 | Hspa8 | 0.58 | 12.95 | 7.21E-05 | 2.41E-03 |
| 214597 | Sidt2 | -0.20 | 8.80 | 7.25E-05 | 2.41E-03 |
| 18642 | Pfkm | 0.44 | 8.99 | 7.25E-05 | 2.41E-03 |
| 21854 | Timm17a | -0.19 | 11.43 | 7.29E-05 | 2.41E-03 |
| 56550 | Ube2d2 | 0.17 | 10.61 | 7.57E-05 | 2.48E-03 |
| 27223 | Trp53bp1 | -0.18 | 10.27 | 7.92E-05 | 2.57E-03 |
| 66691 | Gapvd1 | 0.23 | 9.77 | 8.37E-05 | 2.67E-03 |
| 110323 | Cox6b1 | -0.16 | 12.55 | 8.39E-05 | 2.67E-03 |
| 208606 | Rsrc2 | 0.19 | 9.38 | 8.40E-05 | 2.67E-03 |
| 66588 | Cmpk | -0.22 | 9.87 | 8.49E-05 | 2.68E-03 |
| 12554 | Cdh13 | 0.14 | 8.41 | 8.52E-05 | 2.68E-03 |
| 241943 | BC050811 | -0.23 | 8.71 | 8.58E-05 | 2.69E-03 |
| 14800 | Gria2 | 0.32 | 11.06 | 8.84E-05 | 2.74E-03 |
| 16561 | Kif1b | 0.23 | 9.11 | 9.23E-05 | 2.83E-03 |
| 30963 | Ptpla | -0.16 | 9.24 | 9.48E-05 | 2.87E-03 |
| 76687 | Spcs3 | 0.18 | 9.75 | 9.94E-05 | 2.99E-03 |
| 69029 | 1500032L24Rik | -0.19 | 11.30 | 1.01E-04 | 3.02E-03 |
| 24067 | Srp54 | 0.28 | 9.02 | 1.02E-04 | 3.03E-03 |
| 223649 | Nrbp2 | 0.15 | 10.90 | 1.03E-04 | 3.05E-03 |
| 67236 | 2810452K22Rik | -0.15 | 9.85 | 1.03E-04 | 3.05E-03 |
| 320319 | E330018D03Rik | 0.15 | 8.68 | 1.04E-04 | 3.06E-03 |
| 11772 | Ap2a2 | -0.24 | 10.30 | 1.06E-04 | 3.09E-03 |
| 20743 | Spnb3 | -0.27 | 12.13 | 1.06E-04 | 3.10E-03 |
| 245688 | Rbbp7 | 0.22 | 9.73 | 1.07E-04 | 3.11E-03 |
| 100046136 | LOC100046136 | 0.14 | 8.64 | 1.07E-04 | 3.11E-03 |
| 272589 | Tbcel | 0.15 | 10.22 | 1.08E-04 | 3.12E-03 |
| 66840 | Wdr45l | 0.20 | 8.83 | 1.12E-04 | 3.19E-03 |
| 19261 | Sirpa | 0.22 | 8.59 | 1.19E-04 | 3.35E-03 |
| 72961 | Slc17a7 | -0.28 | 12.05 | 1.21E-04 | 3.36E-03 |
| 68035 | Rbm42 | -0.16 | 9.64 | 1.22E-04 | 3.37E-03 |
| 236539 | Phgdh | 0.15 | 8.71 | 1.22E-04 | 3.37E-03 |
| 11838 | Arc | -0.46 | 10.52 | 1.23E-04 | 3.37E-03 |
| 269643 | Ppp2r2c | -0.20 | 10.40 | 1.23E-04 | 3.37E-03 |
| 12153 | Bmp1 | 0.16 | 9.98 | 1.23E-04 | 3.37E-03 |
| 22134 | Tgoln1 | 0.15 | 9.18 | 1.23E-04 | 3.37E-03 |
| 74480 | Samd4 | 0.16 | 8.38 | 1.25E-04 | 3.39E-03 |
| 11551 | Adra2a | -0.21 | 9.47 | 1.28E-04 | 3.45E-03 |
| 18191 | Nrxn3 | 0.29 | 9.46 | 1.29E-04 | 3.46E-03 |
| 70225 | Ppil3 | -0.17 | 9.47 | 1.29E-04 | 3.46E-03 |
| 19341 | Rab4a | -0.17 | 9.47 | 1.30E-04 | 3.47E-03 |
| 75454 | Phpt1 | -0.17 | 10.43 | 1.34E-04 | 3.55E-03 |
| 74322 | Cxxc1 | -0.16 | 9.87 | 1.34E-04 | 3.55E-03 |
| 20312 | Cx3cl1 | 0.17 | 12.59 | 1.36E-04 | 3.58E-03 |
| 76846 | Rps9 | -0.15 | 12.88 | 1.37E-04 | 3.60E-03 |
| 380711 | Garnl4 | -0.22 | 11.19 | 1.38E-04 | 3.61E-03 |
| 23945 | Mgll | 0.15 | 10.98 | 1.40E-04 | 3.64E-03 |
| 230101 | Gba2 | -0.15 | 8.78 | 1.44E-04 | 3.70E-03 |
| 106529 | Gpsn2 | -0.19 | 12.07 | 1.47E-04 | 3.74E-03 |
| 97761 | Sgsm2 | -0.21 | 8.92 | 1.48E-04 | 3.75E-03 |
| 96935 | Susd4 | 0.28 | 10.46 | 1.49E-04 | 3.77E-03 |
| 17965 | Nbl1 | 0.24 | 10.29 | 1.51E-04 | 3.81E-03 |
| 231225 | Tapt1 | 0.14 | 10.55 | 1.52E-04 | 3.82E-03 |
| 12298 | Cacnb4 | 0.17 | 8.42 | 1.57E-04 | 3.92E-03 |
| 104582 | Rprml | -0.18 | 11.15 | 1.58E-04 | 3.92E-03 |
| 15444 | Hpca | -0.23 | 13.07 | 1.59E-04 | 3.95E-03 |
| 67684 | 3300001P08Rik | 0.18 | 11.15 | 1.60E-04 | 3.96E-03 |
| 67938 | Mylc2b | -0.28 | 11.63 | 1.64E-04 | 4.02E-03 |
| 70231 | Gorasp2 | 0.15 | 8.47 | 1.65E-04 | 4.02E-03 |
| 56876 | Nelf | 0.23 | 11.85 | 1.67E-04 | 4.06E-03 |
| 218811 | Sec24c | -0.16 | 9.35 | 1.67E-04 | 4.06E-03 |
| 238023 | Hexdc | 0.14 | 9.36 | 1.68E-04 | 4.06E-03 |
| 73674 | Wdr75 | 0.15 | 9.51 | 1.70E-04 | 4.10E-03 |
| 667977 | EG667977 | 0.14 | 8.54 | 1.73E-04 | 4.14E-03 |
| 50754 | Fbxw7 | 0.20 | 8.81 | 1.74E-04 | 4.15E-03 |
| 94067 | Mrpl43 | -0.17 | 10.08 | 1.77E-04 | 4.17E-03 |
| 269774 | Aak1 | 0.15 | 11.58 | 1.83E-04 | 4.26E-03 |
| 66154 | Tmem14c | -0.16 | 9.90 | 1.85E-04 | 4.29E-03 |
| 12297 | Cacnb3 | -0.28 | 10.61 | 1.93E-04 | 4.44E-03 |
| 20218 | Khdrbs1 | 0.17 | 8.84 | 1.93E-04 | 4.44E-03 |
| 74255 | Smu1 | 0.20 | 9.31 | 1.95E-04 | 4.46E-03 |
| 246179 | Fktn | 0.15 | 8.79 | 1.98E-04 | 4.50E-03 |
| 28075 | Fam152b | 0.16 | 9.84 | 2.05E-04 | 4.59E-03 |
| 19241 | Tmsb4x | -0.28 | 13.25 | 2.09E-04 | 4.63E-03 |
| 15361 | Hmga1 | -0.20 | 9.31 | 2.12E-04 | 4.66E-03 |
| 66043 | Atp5d | -0.21 | 11.37 | 2.12E-04 | 4.66E-03 |
| 18189 | Nrxn1 | 0.31 | 9.73 | 2.12E-04 | 4.66E-03 |
| 74125 | Armc8 | 0.14 | 9.01 | 2.14E-04 | 4.66E-03 |
| 229615 | Pias3 | -0.15 | 8.80 | 2.20E-04 | 4.76E-03 |
| 77629 | 4930544G21Rik | 0.15 | 11.53 | 2.23E-04 | 4.78E-03 |
| 18164 | Nptx1 | -0.16 | 9.04 | 2.26E-04 | 4.84E-03 |
| 217351 | Tnrc6c | 0.17 | 9.65 | 2.27E-04 | 4.84E-03 |
| 29858 | Pmm1 | -0.17 | 10.13 | 2.31E-04 | 4.91E-03 |
| 21858 | Timp2 | -0.25 | 11.05 | 2.34E-04 | 4.96E-03 |
| 13618 | Ednrb | 0.21 | 10.26 | 2.38E-04 | 5.02E-03 |
| 30046 | Zfp292 | 0.18 | 8.66 | 2.39E-04 | 5.04E-03 |
| 27058 | Srp9 | -0.16 | 10.40 | 2.46E-04 | 5.12E-03 |
| 100041835 | LOC100041835 | -0.16 | 12.47 | 2.46E-04 | 5.12E-03 |
| 26570 | Slc7a11 | 0.16 | 9.02 | 2.49E-04 | 5.15E-03 |
| 18483 | Palm | -0.24 | 9.17 | 2.49E-04 | 5.15E-03 |
| 66359 | 2310005N03Rik | -0.17 | 9.24 | 2.53E-04 | 5.21E-03 |
| 108155 | Ogt | 0.18 | 8.86 | 2.54E-04 | 5.21E-03 |
| 20887 | Sult1a1 | -0.18 | 9.18 | 2.57E-04 | 5.24E-03 |
| 20641 | Snrpd1 | -0.18 | 10.55 | 2.58E-04 | 5.24E-03 |
| 13424 | Dync1h1 | -0.27 | 10.68 | 2.62E-04 | 5.31E-03 |
| 19042 | Ppm1a | 0.26 | 9.94 | 2.64E-04 | 5.32E-03 |
| 70238 | Rnf168 | -0.20 | 8.79 | 2.71E-04 | 5.39E-03 |
| 15493 | Hsd3b2 | 0.30 | 10.84 | 2.77E-04 | 5.48E-03 |
| 66411 | Tbcb | -0.18 | 11.06 | 2.78E-04 | 5.49E-03 |
| 21991 | Tpi1 | -0.15 | 13.41 | 2.82E-04 | 5.54E-03 |
| 228608 | Smox | -0.17 | 9.58 | 2.82E-04 | 5.54E-03 |
| 243382 | Ppm1k | 0.19 | 11.11 | 2.88E-04 | 5.61E-03 |
| 19419 | Rasgrp1 | 0.35 | 9.82 | 2.92E-04 | 5.65E-03 |
| 12583 | Cdo1 | -0.17 | 9.07 | 2.93E-04 | 5.65E-03 |
| 94180 | Acsbg1 | 0.17 | 9.28 | 2.95E-04 | 5.69E-03 |
| 18481 | Pak3 | 0.27 | 10.17 | 3.06E-04 | 5.86E-03 |
| 545007 | ENSMUSG00000068790 | 0.14 | 10.35 | 3.09E-04 | 5.88E-03 |
| 59013 | Hnrnph1 | 0.20 | 10.45 | 3.13E-04 | 5.93E-03 |
| 13628 | Eef1a2 | 0.36 | 10.09 | 3.17E-04 | 5.98E-03 |
| 14681 | Gnao1 | 0.21 | 11.26 | 3.21E-04 | 6.04E-03 |
| 16593 | Klc1 | 0.22 | 9.44 | 3.24E-04 | 6.09E-03 |
| 14678 | Gnai2 | -0.18 | 9.59 | 3.45E-04 | 6.38E-03 |
| 14658 | Glrb | 0.24 | 9.02 | 3.47E-04 | 6.40E-03 |
| 382985 | Rrm2b | 0.17 | 9.33 | 3.48E-04 | 6.40E-03 |
| 107767 | Scamp1 | 0.17 | 12.61 | 3.61E-04 | 6.59E-03 |
| 13728 | Mark2 | 0.21 | 11.86 | 3.64E-04 | 6.62E-03 |
| 71538 | Fbxo9 | 0.16 | 10.30 | 3.65E-04 | 6.63E-03 |
| 52615 | Suz12 | 0.24 | 10.21 | 3.70E-04 | 6.67E-03 |
| 68047 | Mpnd | -0.18 | 9.79 | 3.71E-04 | 6.68E-03 |
| 66836 | 0610006I08Rik | -0.17 | 10.88 | 3.72E-04 | 6.68E-03 |
| 12704 | Cit | 0.20 | 8.84 | 3.76E-04 | 6.72E-03 |
| 70495 | Atp6ap2 | 0.30 | 10.45 | 3.79E-04 | 6.73E-03 |
| 268490 | Lsm12 | 0.18 | 8.63 | 3.82E-04 | 6.77E-03 |
| 13000 | Csnk2a2 | 0.18 | 10.66 | 3.85E-04 | 6.79E-03 |
| 100046744 | LOC100046744 | -0.25 | 9.28 | 3.91E-04 | 6.89E-03 |
| 107746 | Rapgef1 | -0.28 | 9.85 | 3.93E-04 | 6.89E-03 |
| 15571 | Elavl3 | 0.21 | 9.76 | 3.94E-04 | 6.90E-03 |
| 546015 | LOC546015 | -0.15 | 12.71 | 3.95E-04 | 6.90E-03 |
| 67216 | Mboat2 | 0.15 | 8.71 | 4.08E-04 | 7.05E-03 |
| 16885 | Limk1 | -0.17 | 9.22 | 4.14E-04 | 7.11E-03 |
| 70439 | Taf15 | -0.16 | 8.45 | 4.14E-04 | 7.11E-03 |
| 20340 | Glg1 | 0.35 | 11.77 | 4.15E-04 | 7.11E-03 |
| 229791 | D3Bwg0562e | 0.19 | 9.84 | 4.19E-04 | 7.13E-03 |
| 66379 | 2310016M24Rik | -0.16 | 11.35 | 4.21E-04 | 7.14E-03 |
| 17318 | Mid1 | -0.18 | 8.28 | 4.28E-04 | 7.22E-03 |
| 320472 | Ppm1e | 0.22 | 9.70 | 4.34E-04 | 7.30E-03 |
| 83962 | Btbd1 | 0.15 | 10.83 | 4.37E-04 | 7.33E-03 |
| 70823 | Hmgb2l1 | 0.14 | 9.20 | 4.48E-04 | 7.38E-03 |
| 100047794 | LOC100047794 | 0.17 | 8.89 | 4.51E-04 | 7.43E-03 |
| 20273 | Scn8a | 0.19 | 8.83 | 4.53E-04 | 7.43E-03 |
| 223666 | D15Wsu169e | -0.18 | 9.41 | 4.53E-04 | 7.43E-03 |
| 16497 | Kcnab1 | 0.14 | 10.25 | 4.57E-04 | 7.46E-03 |
| 66506 | Psmg3 | -0.15 | 8.84 | 4.67E-04 | 7.60E-03 |
| 217310 | C630004H02Rik | -0.16 | 9.19 | 4.78E-04 | 7.69E-03 |
| 75608 | Chmp4b | -0.20 | 9.94 | 4.85E-04 | 7.78E-03 |
| 228355 | Madd | 0.17 | 8.51 | 4.89E-04 | 7.82E-03 |
| 11784 | Apba2 | 0.17 | 9.53 | 4.98E-04 | 7.88E-03 |
| 211548 | Nomo1 | -0.23 | 10.23 | 5.00E-04 | 7.91E-03 |
| 217944 | Rapgef5 | 0.22 | 9.04 | 5.02E-04 | 7.92E-03 |
| 67602 | Necap1 | 0.28 | 9.63 | 5.06E-04 | 7.97E-03 |
| 64011 | Nrgn | -0.18 | 12.57 | 5.07E-04 | 7.97E-03 |
| 72981 | Prkrir | 0.14 | 9.02 | 5.09E-04 | 7.98E-03 |
| 56298 | Atl2 | 0.19 | 8.83 | 5.14E-04 | 8.04E-03 |
| 73094 | Sgip1 | 0.17 | 9.31 | 5.15E-04 | 8.04E-03 |
| 69019 | Spcs1 | -0.16 | 11.19 | 5.33E-04 | 8.27E-03 |
| 227541 | Camk1d | 0.17 | 8.65 | 5.37E-04 | 8.29E-03 |
| 72500 | Ier5l | 0.20 | 9.24 | 5.54E-04 | 8.49E-03 |
| 13640 | Efna5 | 0.24 | 10.04 | 5.58E-04 | 8.53E-03 |
| 15531 | Ndst1 | 0.24 | 9.19 | 5.61E-04 | 8.53E-03 |
| 20910 | Stxbp1 | 0.19 | 8.98 | 5.61E-04 | 8.53E-03 |
| 269608 | Plekhg5 | -0.24 | 9.31 | 5.88E-04 | 8.84E-03 |
| 11767 | Ap1m1 | 0.14 | 9.88 | 5.90E-04 | 8.86E-03 |
| 67041 | Oxct1 | 0.18 | 11.49 | 5.91E-04 | 8.87E-03 |
| 72084 | Pigx | -0.18 | 9.96 | 5.94E-04 | 8.89E-03 |
| 15239 | Hgs | -0.18 | 9.65 | 5.97E-04 | 8.92E-03 |
| 19775 | Xpr1 | -0.16 | 9.72 | 6.03E-04 | 8.92E-03 |
| 106585 | Ankrd12 | 0.30 | 9.34 | 6.04E-04 | 8.92E-03 |
| 217154 | Stac2 | -0.16 | 9.60 | 6.21E-04 | 9.10E-03 |
| 67254 | 2900011O08Rik | 0.28 | 10.32 | 6.25E-04 | 9.14E-03 |
| 72465 | Zfp131 | 0.21 | 9.85 | 6.28E-04 | 9.16E-03 |
| 218397 | Rasa1 | 0.25 | 9.81 | 6.34E-04 | 9.17E-03 |
| 50781 | Dkk3 | 0.16 | 8.42 | 6.35E-04 | 9.17E-03 |
| 75668 | Rasl10a | -0.20 | 9.78 | 6.36E-04 | 9.17E-03 |
| 77125 | Il33 | 0.16 | 8.64 | 6.43E-04 | 9.22E-03 |
| 330941 | AI593442 | 0.30 | 9.23 | 6.60E-04 | 9.34E-03 |
| 54401 | Ywhab | 0.22 | 12.12 | 6.68E-04 | 9.44E-03 |
| 71817 | Tmem50a | -0.22 | 10.86 | 6.75E-04 | 9.52E-03 |
| 57295 | Icmt | -0.22 | 9.55 | 6.83E-04 | 9.57E-03 |
| 11658 | Alcam | 0.23 | 9.15 | 7.10E-04 | 9.83E-03 |
| 16579 | Kifap3 | 0.18 | 11.46 | 7.11E-04 | 9.83E-03 |
| 17991 | Ndufa2 | -0.18 | 12.03 | 7.13E-04 | 9.84E-03 |
| 20168 | Rtn3 | 0.15 | 13.30 | 7.14E-04 | 9.84E-03 |
| 116733 | Vps4a | 0.16 | 9.72 | 7.15E-04 | 9.84E-03 |
| 11941 | Atp2b2 | -0.32 | 10.90 | 7.17E-04 | 9.86E-03 |
| 228942 | Cbln4 | -0.19 | 9.06 | 7.36E-04 | 1.00E-02 |
| 234663 | Dync1li2 | 0.27 | 9.44 | 7.53E-04 | 1.02E-02 |
| 16477 | Junb | -0.16 | 9.14 | 7.54E-04 | 1.02E-02 |
| 232976 | Zfp574 | 0.15 | 9.78 | 7.59E-04 | 1.02E-02 |
| 22359 | Vldlr | 0.24 | 9.42 | 7.74E-04 | 1.04E-02 |
| 229949 | Ak5 | 0.20 | 9.28 | 7.76E-04 | 1.04E-02 |
| 269615 | Plch2 | -0.16 | 9.81 | 7.78E-04 | 1.04E-02 |
| 232910 | Ap2s1 | -0.18 | 11.93 | 7.83E-04 | 1.04E-02 |
| 18213 | Ntrk3 | 0.18 | 8.69 | 8.01E-04 | 1.06E-02 |
| 230249 | AI314180 | 0.19 | 9.11 | 8.04E-04 | 1.06E-02 |
| 56418 | Ykt6 | -0.17 | 9.57 | 8.12E-04 | 1.06E-02 |
| 18710 | Pik3r3 | 0.22 | 9.27 | 8.23E-04 | 1.07E-02 |
| 13722 | Scye1 | -0.16 | 9.95 | 8.27E-04 | 1.07E-02 |
| 12725 | Clcn3 | 0.31 | 10.14 | 8.28E-04 | 1.07E-02 |
| 215653 | Rassf2 | -0.15 | 9.09 | 8.28E-04 | 1.07E-02 |
| 12861 | Cox6a1 | -0.17 | 13.49 | 8.36E-04 | 1.08E-02 |
| 56407 | Trpc4ap | 0.28 | 9.40 | 8.46E-04 | 1.09E-02 |
| 67458 | Ergic1 | -0.15 | 10.16 | 8.59E-04 | 1.10E-02 |
| 80284 | BC003266 | -0.17 | 10.02 | 8.76E-04 | 1.11E-02 |
| 66855 | Tcf25 | 0.20 | 8.63 | 8.96E-04 | 1.13E-02 |
| 18045 | Nfyb | 0.14 | 9.30 | 9.18E-04 | 1.15E-02 |
| 231670 | Fbxo21 | -0.18 | 10.35 | 9.55E-04 | 1.18E-02 |
| 227835 | Gtdc1 | 0.17 | 9.93 | 9.64E-04 | 1.19E-02 |
| 17168 | Mare | 0.18 | 8.77 | 9.85E-04 | 1.21E-02 |
| 22661 | Zfp148 | 0.16 | 8.68 | 9.87E-04 | 1.21E-02 |
| 13193 | Dcx | 0.27 | 8.83 | 9.99E-04 | 1.22E-02 |
| 14199 | Fhl1 | 0.19 | 10.83 | 1.01E-03 | 1.23E-02 |
| 74718 | Snx16 | -0.15 | 9.35 | 1.02E-03 | 1.24E-02 |
| 66366 | Ergic3 | 0.18 | 9.56 | 1.04E-03 | 1.25E-02 |
| 58998 | Pvrl3 | 0.24 | 9.30 | 1.05E-03 | 1.26E-02 |
| 68995 | Mcts1 | -0.16 | 11.26 | 1.05E-03 | 1.26E-02 |
| 30877 | Gnl3 | 0.14 | 9.48 | 1.06E-03 | 1.27E-02 |
| 20843 | Stag2 | 0.14 | 9.08 | 1.06E-03 | 1.27E-02 |
| 226412 | R3hdm1 | 0.29 | 9.27 | 1.06E-03 | 1.27E-02 |
| 233833 | Tnrc6a | 0.21 | 9.76 | 1.10E-03 | 1.29E-02 |
| 100273 | Osbpl9 | 0.15 | 10.48 | 1.12E-03 | 1.30E-02 |
| 229517 | Slc25a44 | -0.20 | 9.70 | 1.12E-03 | 1.30E-02 |
| 14406 | Gabrg2 | 0.14 | 9.22 | 1.13E-03 | 1.31E-02 |
| 225392 | Rell2 | 0.24 | 9.44 | 1.15E-03 | 1.33E-02 |
| 20983 | Syt4 | 0.23 | 11.46 | 1.16E-03 | 1.33E-02 |
| 52850 | Sgsm1 | -0.21 | 9.57 | 1.18E-03 | 1.35E-02 |
| 234854 | Cdk10 | 0.20 | 9.00 | 1.18E-03 | 1.35E-02 |
| 19060 | Ppp5c | 0.17 | 9.45 | 1.20E-03 | 1.36E-02 |
| 100047606 | LOC100047606 | 0.24 | 9.49 | 1.21E-03 | 1.38E-02 |
| 66923 | Pbrm1 | 0.16 | 9.89 | 1.22E-03 | 1.38E-02 |
| 20249 | Scd1 | 0.16 | 10.31 | 1.23E-03 | 1.40E-02 |
| 52040 | Ppp1r10 | -0.18 | 9.06 | 1.27E-03 | 1.43E-02 |
| 217732 | 2310044G17Rik | 0.16 | 9.06 | 1.28E-03 | 1.43E-02 |
| 230868 | Igsf21 | -0.20 | 9.45 | 1.29E-03 | 1.44E-02 |
| 17196 | Mbp | 0.55 | 10.37 | 1.30E-03 | 1.45E-02 |
| 227446 | 2310035C23Rik | 0.15 | 8.66 | 1.31E-03 | 1.45E-02 |
| 21767 | Tex264 | 0.14 | 9.94 | 1.33E-03 | 1.47E-02 |
| 386753 | Dbpht2 | 0.20 | 8.70 | 1.34E-03 | 1.48E-02 |
| 13026 | Pcyt1a | 0.18 | 8.70 | 1.36E-03 | 1.49E-02 |
| 16981 | Lrrn3 | 0.14 | 9.54 | 1.39E-03 | 1.52E-02 |
| 69551 | 2310022B05Rik | 0.16 | 10.09 | 1.42E-03 | 1.54E-02 |
| 66059 | Krtcap2 | -0.16 | 11.17 | 1.43E-03 | 1.55E-02 |
| 66046 | Ndufb5 | -0.15 | 9.43 | 1.44E-03 | 1.55E-02 |
| 20364 | Sepw1 | -0.15 | 11.59 | 1.45E-03 | 1.57E-02 |
| 20384 | Sfrs5 | 0.29 | 10.14 | 1.45E-03 | 1.57E-02 |
| 12322 | Camk2a | 0.19 | 11.21 | 1.46E-03 | 1.57E-02 |
| 11534 | Adk | 0.15 | 10.06 | 1.46E-03 | 1.57E-02 |
| 100045439 | LOC100045439 | 0.18 | 9.68 | 1.46E-03 | 1.57E-02 |
| 170719 | Oxr1 | 0.19 | 11.01 | 1.47E-03 | 1.57E-02 |
| 22153 | Tubb4 | -0.20 | 11.69 | 1.49E-03 | 1.60E-02 |
| 19017 | Ppargc1a | 0.28 | 9.31 | 1.53E-03 | 1.62E-02 |
| 14229 | Fkbp5 | -0.16 | 8.62 | 1.54E-03 | 1.63E-02 |
| 67231 | Tbc1d20 | 0.15 | 9.00 | 1.61E-03 | 1.68E-02 |
| 103694 | Tmed4 | 0.14 | 9.44 | 1.61E-03 | 1.68E-02 |
| 15528 | Hspe1 | -0.17 | 9.27 | 1.62E-03 | 1.69E-02 |
| 13205 | Ddx3x | 0.20 | 9.06 | 1.63E-03 | 1.70E-02 |
| 360216 | Zranb1 | 0.14 | 9.04 | 1.66E-03 | 1.71E-02 |
| 26556 | Homer1 | 0.19 | 11.04 | 1.68E-03 | 1.72E-02 |
| 68365 | Rab14 | 0.24 | 11.32 | 1.71E-03 | 1.74E-02 |
| 228777 | Nrsn2 | 0.15 | 9.67 | 1.73E-03 | 1.76E-02 |
| 94282 | Sfxn5 | -0.16 | 9.30 | 1.73E-03 | 1.76E-02 |
| 233335 | Synm | 0.30 | 9.35 | 1.76E-03 | 1.78E-02 |
| 193742 | Bat5 | 0.17 | 10.82 | 1.77E-03 | 1.78E-02 |
| 108124 | Napa | 0.21 | 9.05 | 1.78E-03 | 1.78E-02 |
| 77045 | Bcl7a | -0.17 | 10.94 | 1.80E-03 | 1.80E-02 |
| 110809 | Sfrs1 | 0.15 | 11.19 | 1.84E-03 | 1.82E-02 |
| 235106 | Hnt | 0.28 | 10.41 | 1.84E-03 | 1.82E-02 |
| 15526 | Hspa9 | 0.16 | 10.28 | 1.84E-03 | 1.82E-02 |
| 23881 | G3bp2 | 0.31 | 10.06 | 1.86E-03 | 1.82E-02 |
| 114886 | Cygb | 0.26 | 9.74 | 1.87E-03 | 1.83E-02 |
| 74563 | Rasgef1c | 0.16 | 9.78 | 1.89E-03 | 1.84E-02 |
| 15452 | Hprt1 | -0.15 | 12.11 | 1.91E-03 | 1.85E-02 |
| 80385 | Tusc2 | 0.14 | 9.82 | 1.93E-03 | 1.87E-02 |
| 19046 | Ppp1cb | 0.33 | 9.92 | 1.94E-03 | 1.87E-02 |
| 11980 | Atp8a1 | 0.16 | 8.50 | 1.94E-03 | 1.87E-02 |
| 70025 | Acot7 | 0.28 | 10.77 | 1.97E-03 | 1.89E-02 |
| 83436 | Plekha2 | 0.21 | 9.36 | 1.98E-03 | 1.89E-02 |
| 70510 | Rnf167 | 0.17 | 8.99 | 1.98E-03 | 1.89E-02 |
| 68585 | Rtn4 | 0.16 | 9.08 | 2.00E-03 | 1.91E-02 |
| 14232 | Fkbp8 | 0.14 | 11.26 | 2.02E-03 | 1.93E-02 |
| 227644 | Snapc4 | -0.16 | 9.16 | 2.06E-03 | 1.95E-02 |
| 68097 | Dynll2 | 0.19 | 8.68 | 2.10E-03 | 1.98E-02 |
| 217342 | Ube2o | 0.21 | 11.90 | 2.10E-03 | 1.98E-02 |
| 17113 | M6pr | 0.15 | 10.39 | 2.18E-03 | 2.03E-02 |
| 56506 | Cib2 | 0.17 | 10.08 | 2.20E-03 | 2.04E-02 |
| 11842 | Arf3 | -0.21 | 12.03 | 2.23E-03 | 2.06E-02 |
| 80987 | Nckipsd | 0.16 | 9.61 | 2.23E-03 | 2.06E-02 |
| 14168 | Fgf13 | 0.18 | 9.15 | 2.24E-03 | 2.06E-02 |
| 18573 | Pde1a | 0.31 | 10.49 | 2.26E-03 | 2.07E-02 |
| 18555 | Pctk1 | -0.19 | 10.56 | 2.26E-03 | 2.07E-02 |
| 74868 | Tmem65 | 0.15 | 11.12 | 2.28E-03 | 2.08E-02 |
| 117171 | 1110038F14Rik | 0.14 | 9.28 | 2.35E-03 | 2.13E-02 |
| 20964 | Syn1 | -0.17 | 13.05 | 2.36E-03 | 2.14E-02 |
| 212518 | Sprn | 0.17 | 9.01 | 2.37E-03 | 2.14E-02 |
| 30853 | Mlf2 | 0.25 | 11.08 | 2.38E-03 | 2.15E-02 |
| 66935 | 1700023B02Rik | 0.18 | 9.08 | 2.40E-03 | 2.15E-02 |
| 72357 | 2210016L21Rik | 0.14 | 9.51 | 2.41E-03 | 2.15E-02 |
| 15510 | Hspd1 | 0.20 | 9.12 | 2.43E-03 | 2.17E-02 |
| 66663 | Uba5 | 0.15 | 10.09 | 2.43E-03 | 2.17E-02 |
| 52897 | D11Bwg0517e | 0.23 | 12.79 | 2.46E-03 | 2.19E-02 |
| 57754 | Cend1 | -0.16 | 10.25 | 2.47E-03 | 2.19E-02 |
| 17775 | Laptm4a | -0.15 | 10.31 | 2.55E-03 | 2.24E-02 |
| 12293 | Cacna2d1 | 0.27 | 8.95 | 2.58E-03 | 2.25E-02 |
| 271457 | Rab5a | 0.24 | 10.45 | 2.64E-03 | 2.30E-02 |
| 17134 | Mafg | -0.16 | 9.95 | 2.68E-03 | 2.32E-02 |
| 12891 | Cpne6 | 0.22 | 12.37 | 2.73E-03 | 2.35E-02 |
| 102866 | Pls3 | 0.16 | 9.73 | 2.74E-03 | 2.35E-02 |
| 21429 | Ubtf | -0.19 | 9.59 | 2.74E-03 | 2.35E-02 |
| 245446 | Slitrk4 | 0.20 | 9.15 | 2.76E-03 | 2.36E-02 |
| 236920 | Stard8 | -0.17 | 9.60 | 2.77E-03 | 2.37E-02 |
| 56736 | Rnf14 | 0.24 | 9.76 | 2.78E-03 | 2.37E-02 |
| 241263 | Gpr158 | 0.15 | 11.39 | 2.79E-03 | 2.38E-02 |
| 71765 | Klhdc3 | -0.15 | 9.48 | 2.81E-03 | 2.39E-02 |
| 58208 | Bcl11b | 0.24 | 10.14 | 2.86E-03 | 2.41E-02 |
| 58802 | Kcnmb4 | -0.17 | 9.52 | 2.88E-03 | 2.42E-02 |
| 140577 | Ankrd6 | 0.15 | 8.90 | 2.89E-03 | 2.43E-02 |
| 230789 | BC008163 | 0.26 | 9.65 | 2.97E-03 | 2.48E-02 |
| 72125 | 2600011E07Rik | 0.26 | 10.23 | 2.99E-03 | 2.49E-02 |
| 56421 | Pfkp | 0.25 | 9.24 | 3.02E-03 | 2.50E-02 |
| 56786 | Tmem9b | 0.14 | 10.74 | 3.02E-03 | 2.50E-02 |
| 213056 | BC049806 | 0.16 | 9.94 | 3.06E-03 | 2.52E-02 |
| 54195 | Gucy1b3 | 0.20 | 9.86 | 3.25E-03 | 2.65E-02 |
| 434858 | EG434858 | 0.17 | 9.48 | 3.36E-03 | 2.72E-02 |
| 18032 | Nfix | 0.21 | 10.94 | 3.46E-03 | 2.77E-02 |
| 54376 | Cacng3 | 0.27 | 9.39 | 3.53E-03 | 2.81E-02 |
| 100045617 | LOC100045617 | 0.20 | 12.59 | 3.65E-03 | 2.87E-02 |
| 100710 | Aprin | 0.14 | 9.29 | 3.83E-03 | 2.96E-02 |
| 14415 | Gad1 | 0.29 | 11.97 | 3.88E-03 | 2.98E-02 |
| 13806 | Eno1 | 0.17 | 12.64 | 3.89E-03 | 2.99E-02 |
| 75089 | Uhrf1bp1l | 0.19 | 9.51 | 3.90E-03 | 2.99E-02 |
| 353187 | Nr1d2 | 0.14 | 9.33 | 3.91E-03 | 2.99E-02 |
| 230316 | Megf9 | 0.14 | 8.62 | 3.98E-03 | 3.03E-02 |
| 75909 | Tmem49 | 0.21 | 9.65 | 3.99E-03 | 3.04E-02 |
| 23859 | Dlg2 | -0.20 | 10.08 | 4.09E-03 | 3.10E-02 |
| 76737 | Creld2 | -0.17 | 8.83 | 4.12E-03 | 3.11E-02 |
| 20775 | Sqle | 0.17 | 8.95 | 4.24E-03 | 3.18E-02 |
| 121022 | Mrps6 | -0.17 | 9.87 | 4.28E-03 | 3.20E-02 |
| 14254 | Flt1 | 0.17 | 10.09 | 4.30E-03 | 3.20E-02 |
| 53378 | Sdcbp | 0.15 | 10.18 | 4.30E-03 | 3.20E-02 |
| 52882 | Rgs7bp | 0.34 | 11.60 | 4.30E-03 | 3.20E-02 |
| 67603 | Dusp6 | -0.16 | 11.16 | 4.32E-03 | 3.21E-02 |
| 67887 | Tmem66 | 0.18 | 10.39 | 4.35E-03 | 3.22E-02 |
| 12933 | Crmp1 | -0.23 | 10.02 | 4.39E-03 | 3.25E-02 |
| 228880 | Prkcbp1 | 0.18 | 9.39 | 4.45E-03 | 3.28E-02 |
| 13138 | Dag1 | 0.15 | 8.65 | 4.48E-03 | 3.29E-02 |
| 57437 | Golga7 | 0.15 | 9.28 | 4.48E-03 | 3.29E-02 |
| 66270 | Fam134b | 0.20 | 9.51 | 4.61E-03 | 3.35E-02 |
| 19736 | Rgs4 | 0.19 | 12.00 | 4.63E-03 | 3.37E-02 |
| 74006 | Dnm1l | 0.17 | 10.57 | 4.67E-03 | 3.39E-02 |
| 20810 | Srm | 0.16 | 9.60 | 4.74E-03 | 3.42E-02 |
| 22110 | Tspyl1 | 0.19 | 9.43 | 4.80E-03 | 3.46E-02 |
| 235402 | Lingo1 | -0.21 | 9.70 | 4.81E-03 | 3.47E-02 |
| 17896 | Myl4 | -0.18 | 10.85 | 4.85E-03 | 3.49E-02 |
| 65114 | Vps35 | 0.19 | 10.32 | 4.86E-03 | 3.49E-02 |
| 24030 | Mrps12 | -0.15 | 10.80 | 4.87E-03 | 3.49E-02 |
| 26938 | St6galnac5 | 0.14 | 9.12 | 4.90E-03 | 3.50E-02 |
| 15275 | Hk1 | 0.18 | 9.05 | 4.97E-03 | 3.54E-02 |
| 52357 | Wwc2 | 0.22 | 10.30 | 5.02E-03 | 3.56E-02 |
| 75956 | Srrm2 | 0.17 | 10.66 | 5.04E-03 | 3.57E-02 |
| 320840 | Negr1 | 0.15 | 8.75 | 5.13E-03 | 3.61E-02 |
| 66039 | D14Ertd449e | -0.16 | 8.97 | 5.30E-03 | 3.70E-02 |
| 382051 | 4833426J09Rik | 0.20 | 9.17 | 5.35E-03 | 3.73E-02 |
| 268709 | Fam107a | -0.21 | 11.27 | 5.43E-03 | 3.77E-02 |
| 269523 | Vcp | 0.17 | 12.01 | 5.46E-03 | 3.78E-02 |
| 217480 | Dgkb | 0.15 | 9.55 | 5.59E-03 | 3.84E-02 |
| 12450 | Ccng1 | 0.14 | 8.76 | 5.67E-03 | 3.87E-02 |
| 100045300 | LOC100045300 | -0.16 | 11.70 | 5.71E-03 | 3.88E-02 |
| 228858 | Gdap1l1 | 0.14 | 9.40 | 5.91E-03 | 3.98E-02 |
| 58248 | 1700123O20Rik | -0.16 | 9.59 | 5.94E-03 | 3.99E-02 |
| 15387 | Hnrnpk | 0.17 | 9.79 | 6.29E-03 | 4.16E-02 |
| 20965 | Syn2 | 0.25 | 12.10 | 6.30E-03 | 4.16E-02 |
| 59041 | Stk25 | 0.14 | 9.52 | 6.57E-03 | 4.27E-02 |
| 67059 | Ola1 | 0.14 | 9.76 | 6.61E-03 | 4.29E-02 |
| 75613 | Med25 | 0.15 | 10.36 | 6.66E-03 | 4.30E-02 |
| 625054 | EG625054 | 0.16 | 13.38 | 6.72E-03 | 4.32E-02 |
| 103711 | Pnpo | 0.15 | 8.95 | 6.74E-03 | 4.33E-02 |
| 67130 | Ndufa6 | -0.16 | 12.05 | 6.79E-03 | 4.35E-02 |
| 100047935 | LOC100047935 | 0.14 | 12.59 | 6.95E-03 | 4.41E-02 |
| 241520 | Fam171b | 0.19 | 11.69 | 7.33E-03 | 4.57E-02 |
| 14571 | Gpd2 | 0.25 | 10.06 | 7.33E-03 | 4.57E-02 |
| 56443 | Arpc1a | 0.17 | 10.40 | 7.33E-03 | 4.57E-02 |
| 104418 | Dgkz | -0.21 | 11.51 | 7.41E-03 | 4.60E-02 |
| 11972 | Atp6v0d1 | 0.21 | 11.15 | 7.47E-03 | 4.62E-02 |
| 16520 | Kcnj4 | 0.14 | 10.16 | 7.49E-03 | 4.62E-02 |
| 23849 | Klf6 | 0.26 | 10.22 | 7.65E-03 | 4.71E-02 |
| 59008 | Anapc5 | 0.15 | 9.48 | 8.19E-03 | 4.93E-02 |
| 52064 | Coq5 | 0.14 | 10.34 | 8.26E-03 | 4.95E-02 |
| 19330 | Rab18 | 0.15 | 9.87 | 8.28E-03 | 4.96E-02 |
| 67800 | Dgat2 | -0.16 | 9.90 | 8.45E-03 | 5.04E-02 |
| 108755 | Lyrm2 | 0.15 | 9.08 | 8.65E-03 | 5.11E-02 |
| 78785 | Clip4 | 0.18 | 9.77 | 8.69E-03 | 5.13E-02 |
| 16500 | Kcnb1 | 0.14 | 9.53 | 8.94E-03 | 5.24E-02 |
| 17957 | Napb | 0.22 | 12.73 | 9.11E-03 | 5.31E-02 |
| 18417 | Cldn11 | -0.22 | 12.07 | 9.27E-03 | 5.37E-02 |
| 67991 | Nacc2 | 0.14 | 10.97 | 9.29E-03 | 5.37E-02 |
| 14396 | Gabra3 | 0.15 | 9.25 | 9.63E-03 | 5.50E-02 |
| 320007 | Sidt1 | 0.15 | 10.10 | 9.64E-03 | 5.51E-02 |
| 18163 | Ctnnd2 | 0.17 | 9.28 | 9.69E-03 | 5.52E-02 |
| 110197 | Dgkg | 0.21 | 9.60 | 9.76E-03 | 5.55E-02 |
| 12053 | Bcl6 | -0.16 | 9.79 | 1.00E-02 | 5.65E-02 |
| 381633 | Gm1673 | -0.18 | 10.12 | 1.00E-02 | 5.66E-02 |
| 17183 | Matn4 | 0.15 | 8.82 | 1.01E-02 | 5.68E-02 |
| 16969 | Zbtb7a | 0.31 | 10.05 | 1.02E-02 | 5.72E-02 |
| 18754 | Prkce | 0.16 | 8.91 | 1.05E-02 | 5.84E-02 |
| 18597 | Pdha1 | 0.14 | 11.46 | 1.05E-02 | 5.85E-02 |
| 20867 | Stip1 | 0.14 | 10.96 | 1.07E-02 | 5.93E-02 |
| 53612 | Vti1b | 0.19 | 10.50 | 1.09E-02 | 5.98E-02 |
| 239102 | Zfhx2 | 0.16 | 10.11 | 1.10E-02 | 6.02E-02 |
| 216976 | BC030499 | 0.15 | 8.60 | 1.12E-02 | 6.10E-02 |
| 100040573 | LOC100040573 | 0.20 | 10.04 | 1.12E-02 | 6.10E-02 |
| 319173 | Hist1h2af | 0.14 | 8.86 | 1.20E-02 | 6.39E-02 |
| 224118 | 1700021K19Rik | -0.16 | 9.47 | 1.21E-02 | 6.45E-02 |
| 67768 | N6amt1 | 0.19 | 9.64 | 1.25E-02 | 6.58E-02 |
| 68268 | Zdhhc21 | 0.19 | 9.09 | 1.26E-02 | 6.62E-02 |
| 104570 | Smek2 | 0.14 | 8.75 | 1.26E-02 | 6.63E-02 |
| 242083 | Ppm1l | 0.20 | 9.51 | 1.29E-02 | 6.69E-02 |
| 11636 | Ak1 | -0.20 | 9.56 | 1.30E-02 | 6.74E-02 |
| 54473 | Tollip | 0.18 | 9.19 | 1.32E-02 | 6.79E-02 |
| 13170 | Dbp | 0.14 | 12.28 | 1.32E-02 | 6.80E-02 |
| 384783 | Irs2 | 0.18 | 9.48 | 1.38E-02 | 6.99E-02 |
| 13385 | Dlg4 | -0.16 | 9.39 | 1.39E-02 | 7.02E-02 |
| 74053 | Grip1 | 0.14 | 9.05 | 1.39E-02 | 7.04E-02 |
| 20512 | Slc1a3 | 0.17 | 13.38 | 1.40E-02 | 7.04E-02 |
| 16510 | Kcnh1 | 0.24 | 9.46 | 1.46E-02 | 7.27E-02 |
| 108645 | Mat2b | 0.16 | 9.62 | 1.63E-02 | 7.86E-02 |
| 19266 | Ptprd | 0.16 | 11.62 | 1.66E-02 | 7.92E-02 |
| 67526 | Atg12 | -0.16 | 10.13 | 1.66E-02 | 7.93E-02 |
| 103742 | 1810046J19Rik | -0.16 | 11.82 | 1.69E-02 | 8.03E-02 |
| 14799 | Gria1 | 0.16 | 9.10 | 1.73E-02 | 8.16E-02 |
| 100048483 | LOC100048483 | -0.15 | 13.69 | 1.74E-02 | 8.17E-02 |
| 64296 | Abhd8 | -0.16 | 10.90 | 1.74E-02 | 8.17E-02 |
| 236604 | 4933439C20Rik | 0.21 | 11.04 | 1.76E-02 | 8.22E-02 |
| 21924 | Tnnc1 | -0.16 | 10.63 | 1.83E-02 | 8.46E-02 |
| 27373 | Csnk1e | 0.20 | 10.23 | 1.84E-02 | 8.50E-02 |
| 100045981 | LOC100045981 | 0.15 | 13.97 | 1.87E-02 | 8.60E-02 |
| 100046320 | LOC100046320 | 0.16 | 9.52 | 1.88E-02 | 8.63E-02 |
| 80890 | Trim2 | 0.24 | 11.26 | 1.92E-02 | 8.74E-02 |
| 668171 | Zxda | 0.23 | 9.77 | 1.92E-02 | 8.75E-02 |
| 110172 | Slc35b1 | 0.17 | 10.75 | 1.92E-02 | 8.75E-02 |
| 20394 | Scg5 | -0.16 | 12.27 | 2.01E-02 | 9.02E-02 |
| 319478 | Cxxc4 | 0.18 | 9.06 | 2.03E-02 | 9.09E-02 |
| 53376 | Usp2 | 0.22 | 10.36 | 2.08E-02 | 9.24E-02 |
| 210027 | Slc35f3 | 0.20 | 10.05 | 2.12E-02 | 9.33E-02 |
| 19384 | Ran | 0.16 | 9.47 | 2.12E-02 | 9.35E-02 |
| 21685 | Tef | 0.15 | 10.88 | 2.21E-02 | 9.61E-02 |
| 219228 | Pcdh17 | 0.21 | 9.73 | 2.21E-02 | 9.63E-02 |
| 16508 | Kcnd2 | 0.17 | 9.10 | 2.25E-02 | 9.73E-02 |

**Table S2. Overrepresentation analysis of miRNA targets among upregulated genes.**

**Table S3. Overrepresentation analysis of miRNA targets among downregulated genes.**

**Table S4. Cell type-specific modular enrichment**.

**Table S5. Functional enrichment analysis for red module genes with high GS and high MM.** Number of genes in query 63, FDR cutoff 0.2.

ToppGene functional enrichment analysis

KEGG pathway enrichment analysis (Bioconductor KEGGprofiler package)

**Table S6. Functional enrichment analysis for brown module genes with high GS and high MM.** Number of genes in query 103, FDR cutoff 0.2.

ToppGene functional enrichment analysis

Table S6(continuation). Functional enrichment analysis for brown module genes with high GS and high MM. Number of genes in query 103, FDR cutoff 0.2

KEGG pathway enrichment analysis (Bioconductor KEGGprofiler package)

**Table S7. Functional enrichment analysis for turquoise module genes with high GS and high MM.** Number of genes in query 185, FDR cutoff 0.2.

ToppGene functional enrichment analysis

KEGG pathway enrichment analysis (Bioconductor KEGGprofiler package)

**Table S8. Prediction of miRNA-mRNA interactions based on expression correlation patterns between single mRNAs and single differentially expressed (alcohol-responsive) miRNAs.** Gene and miRNA expression (“paired”) profiles were obtained for each sample. FDR cutoff 10% (FDR < 0.1).

| **Entrez ID** | **Symbol** | **miRNA ID** | **cor** | **P.value** | **FDR** |
| --- | --- | --- | --- | --- | --- |
| 54354 | Rassf5 | mmu-let-7g-5p | -0.52 | 2.08E-03 | 9.33E-02 |
| 78926 | Gas2l1 | mmu-let-7g-5p | -0.61 | 2.03E-04 | 3.75E-02 |
| 218952 | Fermt2 | mmu-let-7g-5p | 0.59 | 4.32E-04 | 4.87E-02 |
| 73710 | Tubb2b | mmu-let-7g-5p | 0.58 | 5.39E-04 | 5.29E-02 |
| 110891 | Slc8a2 | mmu-let-7g-5p | -0.64 | 7.05E-05 | 3.09E-02 |
| 320563 | Islr2 | mmu-let-7g-5p | -0.54 | 1.50E-03 | 8.17E-02 |
| 15199 | Hebp1 | mmu-let-7g-5p | -0.57 | 7.02E-04 | 5.66E-02 |
| 57740 | Stk32c | mmu-let-7g-5p | -0.62 | 1.66E-04 | 3.71E-02 |
| 266781 | Snx17 | mmu-let-7g-5p | 0.60 | 3.02E-04 | 4.55E-02 |
| 218194 | Phactr1 | mmu-let-7g-5p | 0.64 | 8.02E-05 | 3.19E-02 |
| 11461 | Actb | mmu-let-7g-5p | 0.52 | 2.11E-03 | 9.33E-02 |
| 13629 | Eef2 | mmu-let-7g-5p | 0.53 | 1.97E-03 | 9.21E-02 |
| 54616 | Extl3 | mmu-let-7g-5p | -0.58 | 4.46E-04 | 4.87E-02 |
| 78928 | Pigt | mmu-let-7g-5p | -0.63 | 1.26E-04 | 3.19E-02 |
| 13498 | Atn1 | mmu-let-7g-5p | -0.60 | 3.28E-04 | 4.62E-02 |
| 242773 | Slc45a1 | mmu-let-7g-5p | -0.70 | 6.90E-06 | 9.55E-03 |
| 56219 | Extl1 | mmu-let-7g-5p | -0.54 | 1.47E-03 | 8.17E-02 |
| 74383 | Ubap2l | mmu-let-7g-5p | -0.59 | 3.91E-04 | 4.74E-02 |
| 20525 | Slc2a1 | mmu-let-7g-5p | -0.58 | 5.37E-04 | 5.29E-02 |
| 235431 | Coro2b | mmu-let-7g-5p | -0.57 | 6.58E-04 | 5.51E-02 |
| 18190 | Nrxn2 | mmu-let-7g-5p | -0.58 | 4.85E-04 | 5.00E-02 |
| 14086 | Fscn1 | mmu-let-7g-5p | -0.56 | 9.18E-04 | 6.30E-02 |
| 100046003 | LOC100046003 | mmu-let-7g-5p | -0.59 | 4.01E-04 | 4.74E-02 |
| 54371 | Chst2 | mmu-let-7g-5p | -0.59 | 3.84E-04 | 4.74E-02 |
| 52915 | Zmiz2 | mmu-let-7g-5p | -0.58 | 4.79E-04 | 5.00E-02 |
| 229521 | Syt11 | mmu-let-7g-5p | 0.55 | 1.01E-03 | 6.60E-02 |
| 71770 | Ap2b1 | mmu-let-7g-5p | -0.60 | 2.72E-04 | 4.45E-02 |
| 18626 | Per1 | mmu-let-7g-5p | -0.55 | 1.22E-03 | 7.33E-02 |
| 20779 | Src | mmu-let-7g-5p | -0.58 | 5.45E-04 | 5.29E-02 |
| 69080 | Gmppa | mmu-let-7g-5p | 0.59 | 4.11E-04 | 4.74E-02 |
| 73750 | Whrn | mmu-let-7g-5p | -0.57 | 6.61E-04 | 5.51E-02 |
| 56395 | Tmem115 | mmu-let-7g-5p | -0.55 | 1.14E-03 | 7.10E-02 |
| 242667 | Dlgap3 | mmu-let-7g-5p | -0.53 | 1.98E-03 | 9.21E-02 |
| 78465 | 1700084C01Rik | mmu-let-7g-5p | -0.56 | 9.55E-04 | 6.33E-02 |
| 232791 | Cnot3 | mmu-let-7g-5p | -0.54 | 1.38E-03 | 7.84E-02 |
| 21968 | Tom1 | mmu-let-7g-5p | 0.58 | 5.06E-04 | 5.12E-02 |
| 100047659 | LOC100047659 | mmu-let-7g-5p | -0.56 | 7.63E-04 | 5.85E-02 |
| 57776 | Ttyh1 | mmu-let-7g-5p | 0.55 | 1.26E-03 | 7.46E-02 |
| 216881 | Wscd1 | mmu-let-7g-5p | -0.56 | 8.90E-04 | 6.21E-02 |
| 27374 | Prmt5 | mmu-let-7g-5p | 0.55 | 1.02E-03 | 6.61E-02 |
| 105246 | Brd9 | mmu-let-7g-5p | 0.52 | 2.04E-03 | 9.31E-02 |
| 64144 | Mllt1 | mmu-let-7g-5p | -0.54 | 1.45E-03 | 8.17E-02 |
| 21375 | Tbr1 | mmu-let-7g-5p | -0.61 | 1.83E-04 | 3.71E-02 |
| 75751 | Ipo4 | mmu-let-7g-5p | -0.64 | 6.75E-05 | 3.09E-02 |
| 11932 | Atp1b2 | mmu-let-7g-5p | -0.58 | 4.42E-04 | 4.87E-02 |
| 57783 | Tnip1 | mmu-let-7g-5p | -0.60 | 2.66E-04 | 4.45E-02 |
| 333331 | LOC333331 | mmu-let-7g-5p | 0.63 | 1.17E-04 | 3.19E-02 |
| 21927 | Tnfaip1 | mmu-let-7g-5p | 0.52 | 2.15E-03 | 9.40E-02 |
| 26562 | Ncdn | mmu-let-7g-5p | -0.56 | 8.54E-04 | 6.12E-02 |
| 72503 | 2610507B11Rik | mmu-let-7g-5p | 0.65 | 4.93E-05 | 2.96E-02 |
| 99010 | Lpcat4 | mmu-let-7g-5p | -0.60 | 2.71E-04 | 4.45E-02 |
| 19025 | Ctsa | mmu-let-7g-5p | 0.72 | 3.22E-06 | 5.47E-03 |
| 21807 | Tsc22d1 | mmu-let-7g-5p | 0.60 | 2.99E-04 | 4.55E-02 |
| 53379 | Hnrnpa2b1 | mmu-let-7g-5p | 0.67 | 3.25E-05 | 2.56E-02 |
| 432763 | Prr7 | mmu-let-7g-5p | -0.63 | 9.84E-05 | 3.19E-02 |
| 22278 | Usf1 | mmu-let-7g-5p | 0.55 | 1.14E-03 | 7.10E-02 |
| 19415 | Rasal1 | mmu-let-7g-5p | -0.59 | 3.80E-04 | 4.74E-02 |
| 71310 | Tbc1d9 | mmu-let-7g-5p | -0.60 | 3.02E-04 | 4.55E-02 |
| 59003 | Maea | mmu-let-7g-5p | 0.59 | 3.37E-04 | 4.67E-02 |
| 224997 | Dlgap1 | mmu-let-7g-5p | 0.63 | 9.84E-05 | 3.19E-02 |
| 276770 | Eif5a | mmu-let-7g-5p | 0.66 | 3.39E-05 | 2.56E-02 |
| 94232 | Ubqln4 | mmu-let-7g-5p | -0.57 | 6.04E-04 | 5.46E-02 |
| 67087 | Ctnnbip1 | mmu-let-7g-5p | -0.59 | 3.49E-04 | 4.70E-02 |
| 76898 | B3gat1 | mmu-let-7g-5p | -0.63 | 1.31E-04 | 3.19E-02 |
| 217866 | Cdc42bpb | mmu-let-7g-5p | -0.54 | 1.53E-03 | 8.20E-02 |
| 76820 | D12Ertd553e | mmu-let-7g-5p | 0.59 | 3.67E-04 | 4.70E-02 |
| 56258 | Hnrnph2 | mmu-let-7g-5p | 0.60 | 3.27E-04 | 4.62E-02 |
| 20692 | Sparc | mmu-let-7g-5p | -0.60 | 3.25E-04 | 4.62E-02 |
| 18642 | Pfkm | mmu-let-7g-5p | 0.67 | 2.35E-05 | 2.44E-02 |
| 243819 | Saps1 | mmu-let-7g-5p | -0.59 | 4.00E-04 | 4.74E-02 |
| 12545 | Cdc7 | mmu-let-7g-5p | 0.56 | 9.50E-04 | 6.33E-02 |
| 11518 | Add1 | mmu-let-7g-5p | 0.52 | 2.21E-03 | 9.46E-02 |
| 319477 | 6030419C18Rik | mmu-let-7g-5p | -0.57 | 6.25E-04 | 5.50E-02 |
| 20743 | Spnb3 | mmu-let-7g-5p | -0.56 | 9.60E-04 | 6.33E-02 |
| 100046136 | LOC100046136 | mmu-let-7g-5p | 0.74 | 1.21E-06 | 5.13E-03 |
| 225642 | Grp | mmu-let-7g-5p | -0.55 | 1.04E-03 | 6.67E-02 |
| 236539 | Phgdh | mmu-let-7g-5p | 0.65 | 5.66E-05 | 2.96E-02 |
| 22404 | Wiz | mmu-let-7g-5p | -0.57 | 6.76E-04 | 5.54E-02 |
| 18223 | Numbl | mmu-let-7g-5p | -0.56 | 8.85E-04 | 6.21E-02 |
| 97761 | Sgsm2 | mmu-let-7g-5p | -0.54 | 1.58E-03 | 8.38E-02 |
| 100608 | Noc4l | mmu-let-7g-5p | -0.62 | 1.49E-04 | 3.43E-02 |
| 70231 | Gorasp2 | mmu-let-7g-5p | 0.61 | 2.09E-04 | 3.75E-02 |
| 100046959 | LOC100046959 | mmu-let-7g-5p | 0.53 | 1.95E-03 | 9.21E-02 |
| 11545 | Parp1 | mmu-let-7g-5p | -0.59 | 3.68E-04 | 4.70E-02 |
| 12297 | Cacnb3 | mmu-let-7g-5p | -0.52 | 2.38E-03 | 9.93E-02 |
| 15114 | Hap1 | mmu-let-7g-5p | 0.55 | 1.05E-03 | 6.73E-02 |
| 66513 | Map3k7ip1 | mmu-let-7g-5p | -0.61 | 1.81E-04 | 3.71E-02 |
| 15361 | Hmga1 | mmu-let-7g-5p | -0.53 | 1.61E-03 | 8.42E-02 |
| 229615 | Pias3 | mmu-let-7g-5p | -0.64 | 8.72E-05 | 3.19E-02 |
| 21858 | Timp2 | mmu-let-7g-5p | -0.58 | 5.71E-04 | 5.39E-02 |
| 56149 | Grasp | mmu-let-7g-5p | 0.57 | 6.54E-04 | 5.51E-02 |
| 81018 | Rnf114 | mmu-let-7g-5p | 0.57 | 6.81E-04 | 5.54E-02 |
| 69195 | Tmem121 | mmu-let-7g-5p | -0.54 | 1.53E-03 | 8.20E-02 |
| 71777 | Ing3 | mmu-let-7g-5p | 0.57 | 7.13E-04 | 5.69E-02 |
| 13628 | Eef1a2 | mmu-let-7g-5p | 0.68 | 1.86E-05 | 2.21E-02 |
| 71947 | 2310067B10Rik | mmu-let-7g-5p | -0.61 | 1.97E-04 | 3.75E-02 |
| 545554 | Ankrd34a | mmu-let-7g-5p | -0.63 | 1.17E-04 | 3.19E-02 |
| 100046744 | LOC100046744 | mmu-let-7g-5p | -0.73 | 1.85E-06 | 5.13E-03 |
| 57357 | Srd5a3 | mmu-let-7g-5p | -0.53 | 1.94E-03 | 9.21E-02 |
| 17318 | Mid1 | mmu-let-7g-5p | -0.56 | 7.76E-04 | 5.85E-02 |
| 67905 | Ppm1m | mmu-let-7g-5p | -0.59 | 4.35E-04 | 4.87E-02 |
| 71916 | Dus4l | mmu-let-7g-5p | -0.56 | 8.17E-04 | 5.96E-02 |
| 66830 | Nacc1 | mmu-let-7g-5p | -0.55 | 1.23E-03 | 7.35E-02 |
| 228355 | Madd | mmu-let-7g-5p | 0.65 | 5.55E-05 | 2.96E-02 |
| 230996 | 9430015G10Rik | mmu-let-7g-5p | -0.53 | 1.76E-03 | 8.71E-02 |
| 211548 | Nomo1 | mmu-let-7g-5p | -0.58 | 5.48E-04 | 5.29E-02 |
| 69780 | Smap2 | mmu-let-7g-5p | -0.52 | 2.08E-03 | 9.33E-02 |
| 20910 | Stxbp1 | mmu-let-7g-5p | 0.62 | 1.37E-04 | 3.25E-02 |
| 269608 | Plekhg5 | mmu-let-7g-5p | -0.56 | 7.90E-04 | 5.86E-02 |
| 73826 | Poldip3 | mmu-let-7g-5p | 0.56 | 8.33E-04 | 6.02E-02 |
| 233033 | Samd4b | mmu-let-7g-5p | -0.53 | 1.73E-03 | 8.69E-02 |
| 16906 | Lmnb1 | mmu-let-7g-5p | -0.60 | 3.14E-04 | 4.62E-02 |
| 18710 | Pik3r3 | mmu-let-7g-5p | 0.58 | 5.58E-04 | 5.32E-02 |
| 66366 | Ergic3 | mmu-let-7g-5p | 0.53 | 1.70E-03 | 8.68E-02 |
| 56316 | Ggcx | mmu-let-7g-5p | -0.54 | 1.49E-03 | 8.17E-02 |
| 14661 | Lgsn | mmu-let-7g-5p | 0.72 | 3.29E-06 | 5.47E-03 |
| 229517 | Slc25a44 | mmu-let-7g-5p | -0.55 | 1.20E-03 | 7.30E-02 |
| 234854 | Cdk10 | mmu-let-7g-5p | 0.52 | 2.25E-03 | 9.58E-02 |
| 52040 | Ppp1r10 | mmu-let-7g-5p | -0.64 | 8.89E-05 | 3.19E-02 |
| 17196 | Mbp | mmu-let-7g-5p | 0.55 | 1.08E-03 | 6.85E-02 |
| 228998 | Arfgap1 | mmu-let-7g-5p | 0.53 | 1.93E-03 | 9.21E-02 |
| 16796 | Lasp1 | mmu-let-7g-5p | -0.53 | 1.74E-03 | 8.69E-02 |
| 14229 | Fkbp5 | mmu-let-7g-5p | -0.53 | 2.03E-03 | 9.31E-02 |
| 219022 | Ttc5 | mmu-let-7g-5p | 0.55 | 1.16E-03 | 7.17E-02 |
| 240753 | Plekha6 | mmu-let-7g-5p | 0.54 | 1.27E-03 | 7.48E-02 |
| 69719 | Cad | mmu-let-7g-5p | -0.56 | 7.71E-04 | 5.85E-02 |
| 70025 | Acot7 | mmu-let-7g-5p | 0.66 | 3.30E-05 | 2.56E-02 |
| 70510 | Rnf167 | mmu-let-7g-5p | 0.61 | 2.10E-04 | 3.75E-02 |
| 68585 | Rtn4 | mmu-let-7g-5p | 0.55 | 1.17E-03 | 7.17E-02 |
| 140580 | Elmo1 | mmu-let-7g-5p | 0.56 | 9.42E-04 | 6.33E-02 |
| 69106 | Stoml1 | mmu-let-7g-5p | 0.52 | 2.11E-03 | 9.33E-02 |
| 74763 | Nat15 | mmu-let-7g-5p | 0.57 | 7.46E-04 | 5.85E-02 |
| 233900 | Rnf40 | mmu-let-7g-5p | -0.61 | 2.12E-04 | 3.75E-02 |
| 30853 | Mlf2 | mmu-let-7g-5p | 0.56 | 8.99E-04 | 6.22E-02 |
| 15510 | Hspd1 | mmu-let-7g-5p | 0.52 | 2.16E-03 | 9.40E-02 |
| 21429 | Ubtf | mmu-let-7g-5p | -0.63 | 1.23E-04 | 3.19E-02 |
| 236899 | Pcyt1b | mmu-let-7g-5p | -0.52 | 2.10E-03 | 9.33E-02 |
| 434858 | EG434858 | mmu-let-7g-5p | 0.63 | 1.00E-04 | 3.19E-02 |
| 57265 | Fzd2 | mmu-let-7g-5p | -0.56 | 7.65E-04 | 5.85E-02 |
| 54376 | Cacng3 | mmu-let-7g-5p | 0.53 | 2.00E-03 | 9.23E-02 |
| 279766 | Rhbdd3 | mmu-let-7g-5p | -0.64 | 7.08E-05 | 3.09E-02 |
| 212627 | Prpsap2 | mmu-let-7g-5p | 0.54 | 1.48E-03 | 8.17E-02 |
| 241846 | Lsm14b | mmu-let-7g-5p | -0.65 | 5.71E-05 | 2.96E-02 |
| 23859 | Dlg2 | mmu-let-7g-5p | -0.54 | 1.59E-03 | 8.38E-02 |
| 66431 | 1810049H13Rik | mmu-let-7g-5p | -0.53 | 1.84E-03 | 8.92E-02 |
| 194237 | BC057371 | mmu-let-7g-5p | 0.53 | 1.73E-03 | 8.69E-02 |
| 12933 | Crmp1 | mmu-let-7g-5p | -0.52 | 2.37E-03 | 9.92E-02 |
| 57437 | Golga7 | mmu-let-7g-5p | 0.57 | 7.19E-04 | 5.69E-02 |
| 21885 | Tle1 | mmu-let-7g-5p | -0.52 | 2.12E-03 | 9.33E-02 |
| 94280 | Sfxn3 | mmu-let-7g-5p | -0.53 | 1.62E-03 | 8.43E-02 |
| 20810 | Srm | mmu-let-7g-5p | 0.58 | 4.80E-04 | 5.00E-02 |
| 235402 | Lingo1 | mmu-let-7g-5p | -0.56 | 7.90E-04 | 5.86E-02 |
| 100044576 | LOC100044576 | mmu-let-7g-5p | -0.53 | 1.83E-03 | 8.92E-02 |
| 50935 | St6galnac6 | mmu-let-7g-5p | 0.52 | 2.28E-03 | 9.68E-02 |
| 329777 | Pigk | mmu-let-7g-5p | 0.60 | 2.73E-04 | 4.45E-02 |
| 213742 | Xist | mmu-let-7g-5p | -0.62 | 1.80E-04 | 3.71E-02 |
| 20623 | Snrk | mmu-let-7g-5p | 0.53 | 1.64E-03 | 8.47E-02 |
| 17183 | Matn4 | mmu-let-7g-5p | 0.54 | 1.29E-03 | 7.52E-02 |
| 80906 | Kcnip2 | mmu-let-7g-5p | 0.53 | 1.89E-03 | 9.14E-02 |
| 216976 | BC030499 | mmu-let-7g-5p | 0.57 | 6.12E-04 | 5.46E-02 |
| 27096 | Trappc3 | mmu-let-7g-5p | 0.63 | 1.31E-04 | 3.19E-02 |
| 65257 | Asb3 | mmu-let-7g-5p | 0.59 | 3.53E-04 | 4.70E-02 |
| 100732 | Mapre3 | mmu-let-7g-5p | -0.60 | 2.95E-04 | 4.55E-02 |
| 58887 | Repin1 | mmu-let-7g-5p | -0.53 | 1.80E-03 | 8.84E-02 |
| 13385 | Dlg4 | mmu-let-7g-5p | -0.55 | 1.18E-03 | 7.22E-02 |
| 102657 | Cd276 | mmu-let-7g-5p | -0.62 | 1.74E-04 | 3.71E-02 |
| 57370 | B4galt3 | mmu-let-7g-5p | 0.52 | 2.19E-03 | 9.43E-02 |
| 56807 | Scamp5 | mmu-let-7g-5p | 0.53 | 1.75E-03 | 8.69E-02 |
| 30934 | Tor1b | mmu-let-7g-5p | -0.63 | 1.17E-04 | 3.19E-02 |
| 240025 | Dact2 | mmu-let-7g-5p | -0.57 | 5.93E-04 | 5.46E-02 |
| 16834 | Cog1 | mmu-let-7g-5p | -0.54 | 1.35E-03 | 7.75E-02 |
| 13384 | Mpp3 | mmu-let-7g-5p | 0.57 | 6.05E-04 | 5.46E-02 |
| 11993 | Aup1 | mmu-let-7g-5p | 0.74 | 1.40E-06 | 5.13E-03 |
| 11769 | Ap1s1 | mmu-let-7g-5p | 0.56 | 9.38E-04 | 6.33E-02 |
| 594844 | Tceal3 | mmu-let-7g-5p | 0.52 | 2.08E-03 | 9.33E-02 |
| 27355 | X99384 | mmu-let-7g-5p | -0.57 | 6.54E-04 | 5.51E-02 |
| 231148 | Ablim2 | mmu-let-7g-5p | 0.65 | 5.07E-05 | 2.96E-02 |
| 20586 | Smarca4 | mmu-let-7g-5p | -0.56 | 8.19E-04 | 5.96E-02 |
| 72895 | Setd5 | mmu-let-7g-5p | -0.63 | 1.30E-04 | 3.19E-02 |
| 27643 | Ubl4 | mmu-let-7g-5p | 0.56 | 8.83E-04 | 6.21E-02 |
| 320795 | Pkn1 | mmu-let-7g-5p | -0.59 | 3.59E-04 | 4.70E-02 |
| 24000 | Ptpn21 | mmu-let-7g-5p | -0.58 | 4.88E-04 | 5.00E-02 |
| 227721 | Ppapdc3 | mmu-let-7g-5p | -0.61 | 2.01E-04 | 3.75E-02 |
| 225288 | Fhod3 | mmu-let-7g-5p | -0.57 | 5.86E-04 | 5.46E-02 |
| 53602 | Hpcal1 | mmu-let-7g-5p | 0.52 | 2.36E-03 | 9.92E-02 |
| 13831 | Epc1 | mmu-let-7g-5p | 0.54 | 1.48E-03 | 8.17E-02 |
| 26987 | Eif4e2 | mmu-let-7g-5p | 0.57 | 6.30E-04 | 5.50E-02 |
| 16210 | Impact | mmu-let-7g-5p | 0.54 | 1.34E-03 | 7.72E-02 |
| 230757 | 5730409E04Rik | mmu-let-7g-5p | -0.64 | 8.53E-05 | 3.19E-02 |
| 330222 | Sdk1 | mmu-let-7g-5p | -0.52 | 2.19E-03 | 9.43E-02 |
| 70317 | Arl16 | mmu-let-7g-5p | 0.53 | 1.98E-03 | 9.21E-02 |
| 17760 | Mtap6 | mmu-let-7g-5p | 0.57 | 6.64E-04 | 5.51E-02 |
| 17750 | Mt2 | mmu-let-7g-5p | -0.54 | 1.51E-03 | 8.20E-02 |
| 27407 | Abcf2 | mmu-let-7g-5p | 0.54 | 1.31E-03 | 7.60E-02 |
| 73072 | BC068157 | mmu-let-7g-5p | -0.53 | 1.98E-03 | 9.21E-02 |
| 59049 | Slc22a17 | mmu-let-7g-5p | 0.53 | 1.68E-03 | 8.62E-02 |
| 13356 | Dgcr2 | mmu-let-7g-5p | -0.59 | 4.08E-04 | 4.74E-02 |
| 76789 | 2410129H14Rik | mmu-let-7g-5p | 0.58 | 4.55E-04 | 4.91E-02 |
| 13116 | Cyp46a1 | mmu-let-7g-5p | 0.54 | 1.60E-03 | 8.38E-02 |
| 236539 | Phgdh | mmu-miR-26a-5p | 0.66 | 4.05E-05 | 9.43E-02 |
| 11545 | Parp1 | mmu-miR-26a-5p | -0.68 | 2.22E-05 | 9.43E-02 |
| 20910 | Stxbp1 | mmu-miR-26a-5p | 0.66 | 4.54E-05 | 9.43E-02 |
| 14661 | Lgsn | mmu-miR-26a-5p | 0.67 | 2.49E-05 | 9.43E-02 |
| 242773 | Slc45a1 | mmu-let-7c-5p | -0.66 | 3.55E-05 | 3.28E-02 |
| 75751 | Ipo4 | mmu-let-7c-5p | -0.64 | 7.54E-05 | 4.96E-02 |
| 333331 | LOC333331 | mmu-let-7c-5p | 0.63 | 1.06E-04 | 5.87E-02 |
| 19025 | Ctsa | mmu-let-7c-5p | 0.67 | 2.90E-05 | 3.28E-02 |
| 53379 | Hnrnpa2b1 | mmu-let-7c-5p | 0.65 | 6.29E-05 | 4.75E-02 |
| 432763 | Prr7 | mmu-let-7c-5p | -0.63 | 9.95E-05 | 5.87E-02 |
| 276770 | Eif5a | mmu-let-7c-5p | 0.70 | 8.49E-06 | 2.78E-02 |
| 100046136 | LOC100046136 | mmu-let-7c-5p | 0.69 | 1.49E-05 | 3.09E-02 |
| 236539 | Phgdh | mmu-let-7c-5p | 0.72 | 2.99E-06 | 2.49E-02 |
| 69195 | Tmem121 | mmu-let-7c-5p | -0.65 | 5.93E-05 | 4.75E-02 |
| 100046744 | LOC100046744 | mmu-let-7c-5p | -0.64 | 7.76E-05 | 4.96E-02 |
| 14661 | Lgsn | mmu-let-7c-5p | 0.67 | 3.26E-05 | 3.28E-02 |
| 434858 | EG434858 | mmu-let-7c-5p | 0.67 | 2.40E-05 | 3.28E-02 |
| 11993 | Aup1 | mmu-let-7c-5p | 0.70 | 1.00E-05 | 2.78E-02 |
| 217615 | Ctage5 | mmu-let-7c-5p | 0.68 | 2.16E-05 | 3.28E-02 |
| 54354 | Rassf5 | mmu-miR-30d-5p | -0.58 | 5.74E-04 | 9.23E-02 |
| 78926 | Gas2l1 | mmu-miR-30d-5p | -0.57 | 6.04E-04 | 9.28E-02 |
| 218952 | Fermt2 | mmu-miR-30d-5p | 0.63 | 1.14E-04 | 6.71E-02 |
| 110891 | Slc8a2 | mmu-miR-30d-5p | -0.60 | 3.19E-04 | 8.16E-02 |
| 57740 | Stk32c | mmu-miR-30d-5p | -0.62 | 1.33E-04 | 6.71E-02 |
| 266781 | Snx17 | mmu-miR-30d-5p | 0.60 | 3.05E-04 | 8.16E-02 |
| 242773 | Slc45a1 | mmu-miR-30d-5p | -0.60 | 2.50E-04 | 8.03E-02 |
| 20525 | Slc2a1 | mmu-miR-30d-5p | -0.62 | 1.59E-04 | 6.71E-02 |
| 57776 | Ttyh1 | mmu-miR-30d-5p | 0.58 | 5.72E-04 | 9.23E-02 |
| 15270 | H2afx | mmu-miR-30d-5p | -0.58 | 4.71E-04 | 8.79E-02 |
| 75751 | Ipo4 | mmu-miR-30d-5p | -0.60 | 2.59E-04 | 8.03E-02 |
| 333331 | LOC333331 | mmu-miR-30d-5p | 0.62 | 1.61E-04 | 6.71E-02 |
| 21927 | Tnfaip1 | mmu-miR-30d-5p | 0.61 | 2.12E-04 | 7.65E-02 |
| 99010 | Lpcat4 | mmu-miR-30d-5p | -0.57 | 6.90E-04 | 9.87E-02 |
| 22232 | Slc35a2 | mmu-miR-30d-5p | -0.58 | 4.45E-04 | 8.79E-02 |
| 19025 | Ctsa | mmu-miR-30d-5p | 0.71 | 6.37E-06 | 1.76E-02 |
| 53379 | Hnrnpa2b1 | mmu-miR-30d-5p | 0.62 | 1.39E-04 | 6.71E-02 |
| 224997 | Dlgap1 | mmu-miR-30d-5p | 0.62 | 1.69E-04 | 6.71E-02 |
| 276770 | Eif5a | mmu-miR-30d-5p | 0.69 | 1.52E-05 | 3.10E-02 |
| 18642 | Pfkm | mmu-miR-30d-5p | 0.57 | 6.69E-04 | 9.77E-02 |
| 211712 | Pcdh9 | mmu-miR-30d-5p | 0.60 | 3.12E-04 | 8.16E-02 |
| 319477 | 6030419C18Rik | mmu-miR-30d-5p | -0.63 | 9.71E-05 | 6.20E-02 |
| 100046136 | LOC100046136 | mmu-miR-30d-5p | 0.68 | 2.11E-05 | 3.10E-02 |
| 225642 | Grp | mmu-miR-30d-5p | -0.62 | 1.72E-04 | 6.71E-02 |
| 236539 | Phgdh | mmu-miR-30d-5p | 0.64 | 8.51E-05 | 5.89E-02 |
| 100608 | Noc4l | mmu-miR-30d-5p | -0.58 | 5.40E-04 | 9.15E-02 |
| 70231 | Gorasp2 | mmu-miR-30d-5p | 0.59 | 3.77E-04 | 8.67E-02 |
| 11545 | Parp1 | mmu-miR-30d-5p | -0.64 | 7.80E-05 | 5.89E-02 |
| 74255 | Smu1 | mmu-miR-30d-5p | 0.57 | 6.71E-04 | 9.77E-02 |
| 30937 | Lmcd1 | mmu-miR-30d-5p | 0.64 | 8.30E-05 | 5.89E-02 |
| 69195 | Tmem121 | mmu-miR-30d-5p | -0.58 | 4.40E-04 | 8.79E-02 |
| 71777 | Ing3 | mmu-miR-30d-5p | 0.61 | 2.43E-04 | 8.03E-02 |
| 13628 | Eef1a2 | mmu-miR-30d-5p | 0.59 | 4.20E-04 | 8.79E-02 |
| 71947 | 2310067B10Rik | mmu-miR-30d-5p | -0.58 | 4.37E-04 | 8.79E-02 |
| 545554 | Ankrd34a | mmu-miR-30d-5p | -0.68 | 2.24E-05 | 3.10E-02 |
| 76899 | Golga1 | mmu-miR-30d-5p | 0.60 | 2.68E-04 | 8.03E-02 |
| 100046744 | LOC100046744 | mmu-miR-30d-5p | -0.62 | 1.78E-04 | 6.71E-02 |
| 71916 | Dus4l | mmu-miR-30d-5p | -0.59 | 3.91E-04 | 8.67E-02 |
| 228355 | Madd | mmu-miR-30d-5p | 0.57 | 7.38E-04 | 9.94E-02 |
| 20910 | Stxbp1 | mmu-miR-30d-5p | 0.60 | 3.24E-04 | 8.16E-02 |
| 67420 | Far1 | mmu-miR-30d-5p | 0.60 | 2.71E-04 | 8.03E-02 |
| 66366 | Ergic3 | mmu-miR-30d-5p | 0.57 | 6.56E-04 | 9.77E-02 |
| 14661 | Lgsn | mmu-miR-30d-5p | 0.71 | 4.74E-06 | 1.76E-02 |
| 12608 | Cebpb | mmu-miR-30d-5p | -0.57 | 7.24E-04 | 9.94E-02 |
| 228998 | Arfgap1 | mmu-miR-30d-5p | 0.58 | 4.58E-04 | 8.79E-02 |
| 14229 | Fkbp5 | mmu-miR-30d-5p | -0.67 | 2.62E-05 | 3.11E-02 |
| 219022 | Ttc5 | mmu-miR-30d-5p | 0.60 | 3.13E-04 | 8.16E-02 |
| 76959 | Chmp5 | mmu-miR-30d-5p | -0.58 | 4.76E-04 | 8.79E-02 |
| 74763 | Nat15 | mmu-miR-30d-5p | 0.59 | 3.97E-04 | 8.67E-02 |
| 233900 | Rnf40 | mmu-miR-30d-5p | -0.58 | 4.95E-04 | 8.89E-02 |
| 21429 | Ubtf | mmu-miR-30d-5p | -0.57 | 7.42E-04 | 9.94E-02 |
| 434858 | EG434858 | mmu-miR-30d-5p | 0.65 | 6.62E-05 | 5.89E-02 |
| 279766 | Rhbdd3 | mmu-miR-30d-5p | -0.58 | 5.03E-04 | 8.89E-02 |
| 241846 | Lsm14b | mmu-miR-30d-5p | -0.59 | 3.68E-04 | 8.67E-02 |
| 108115 | Slco4a1 | mmu-miR-30d-5p | -0.57 | 5.97E-04 | 9.28E-02 |
| 30930 | Vps26a | mmu-miR-30d-5p | 0.57 | 5.78E-04 | 9.23E-02 |
| 102657 | Cd276 | mmu-miR-30d-5p | -0.57 | 7.44E-04 | 9.94E-02 |
| 13384 | Mpp3 | mmu-miR-30d-5p | 0.59 | 3.45E-04 | 8.42E-02 |
| 11993 | Aup1 | mmu-miR-30d-5p | 0.73 | 2.29E-06 | 1.76E-02 |
| 11769 | Ap1s1 | mmu-miR-30d-5p | 0.56 | 7.54E-04 | 9.94E-02 |
| 227721 | Ppapdc3 | mmu-miR-30d-5p | -0.65 | 6.30E-05 | 5.89E-02 |
| 17760 | Mtap6 | mmu-miR-30d-5p | 0.62 | 1.57E-04 | 6.71E-02 |
| 83435 | Plekha3 | mmu-miR-30d-5p | -0.58 | 5.24E-04 | 9.06E-02 |
| 54354 | Rassf5 | mmu-miR-34c-5p | -0.60 | 2.96E-04 | 3.24E-02 |
| 67972 | Atp2b1 | mmu-miR-34c-5p | 0.56 | 7.71E-04 | 4.39E-02 |
| 55978 | Ift20 | mmu-miR-34c-5p | -0.54 | 1.29E-03 | 5.48E-02 |
| 78926 | Gas2l1 | mmu-miR-34c-5p | -0.57 | 6.43E-04 | 4.06E-02 |
| 218952 | Fermt2 | mmu-miR-34c-5p | 0.60 | 2.59E-04 | 3.08E-02 |
| 114641 | Rpl31 | mmu-miR-34c-5p | -0.55 | 1.02E-03 | 4.97E-02 |
| 73710 | Tubb2b | mmu-miR-34c-5p | 0.66 | 4.49E-05 | 1.83E-02 |
| 216767 | Mrpl22 | mmu-miR-34c-5p | -0.52 | 2.08E-03 | 6.54E-02 |
| 68262 | Agpat4 | mmu-miR-34c-5p | -0.53 | 1.78E-03 | 6.11E-02 |
| 56632 | Sphk2 | mmu-miR-34c-5p | -0.57 | 6.51E-04 | 4.06E-02 |
| 110891 | Slc8a2 | mmu-miR-34c-5p | -0.65 | 5.75E-05 | 1.91E-02 |
| 320563 | Islr2 | mmu-miR-34c-5p | -0.59 | 3.46E-04 | 3.54E-02 |
| 12349 | Car2 | mmu-miR-34c-5p | 0.52 | 2.21E-03 | 6.67E-02 |
| 19299 | Abcd3 | mmu-miR-34c-5p | 0.53 | 1.61E-03 | 5.90E-02 |
| 11490 | Adam15 | mmu-miR-34c-5p | -0.53 | 1.83E-03 | 6.15E-02 |
| 16716 | Ky | mmu-miR-34c-5p | -0.57 | 5.81E-04 | 4.02E-02 |
| 381677 | Vgf | mmu-miR-34c-5p | -0.57 | 6.85E-04 | 4.12E-02 |
| 15199 | Hebp1 | mmu-miR-34c-5p | -0.59 | 4.23E-04 | 3.74E-02 |
| 57740 | Stk32c | mmu-miR-34c-5p | -0.63 | 1.11E-04 | 2.51E-02 |
| 20018 | Polr1d | mmu-miR-34c-5p | -0.55 | 1.02E-03 | 4.97E-02 |
| 266781 | Snx17 | mmu-miR-34c-5p | 0.77 | 3.28E-07 | 2.72E-03 |
| 14009 | Etv1 | mmu-miR-34c-5p | 0.60 | 2.53E-04 | 3.05E-02 |
| 218194 | Phactr1 | mmu-miR-34c-5p | 0.64 | 8.14E-05 | 2.20E-02 |
| 108013 | Brunol4 | mmu-miR-34c-5p | 0.54 | 1.27E-03 | 5.48E-02 |
| 69833 | Polr2f | mmu-miR-34c-5p | -0.52 | 2.21E-03 | 6.67E-02 |
| 23827 | Bpnt1 | mmu-miR-34c-5p | -0.50 | 3.80E-03 | 8.44E-02 |
| 212090 | Tmem60 | mmu-miR-34c-5p | -0.52 | 2.35E-03 | 6.82E-02 |
| 228410 | Cstf3 | mmu-miR-34c-5p | -0.49 | 4.73E-03 | 9.22E-02 |
| 21681 | Thoc4 | mmu-miR-34c-5p | -0.50 | 3.72E-03 | 8.41E-02 |
| 74277 | Chic2 | mmu-miR-34c-5p | -0.54 | 1.36E-03 | 5.56E-02 |
| 11461 | Actb | mmu-miR-34c-5p | 0.63 | 1.22E-04 | 2.51E-02 |
| 17748 | Mt1 | mmu-miR-34c-5p | -0.66 | 3.49E-05 | 1.81E-02 |
| 13629 | Eef2 | mmu-miR-34c-5p | 0.65 | 4.84E-05 | 1.83E-02 |
| 54616 | Extl3 | mmu-miR-34c-5p | -0.62 | 1.34E-04 | 2.51E-02 |
| 26396 | Map2k2 | mmu-miR-34c-5p | 0.55 | 1.20E-03 | 5.32E-02 |
| 66664 | Tmem41a | mmu-miR-34c-5p | -0.55 | 1.06E-03 | 5.09E-02 |
| 78928 | Pigt | mmu-miR-34c-5p | -0.60 | 2.50E-04 | 3.05E-02 |
| 11782 | Ap4s1 | mmu-miR-34c-5p | -0.61 | 2.27E-04 | 2.92E-02 |
| 13498 | Atn1 | mmu-miR-34c-5p | -0.58 | 4.78E-04 | 3.74E-02 |
| 242773 | Slc45a1 | mmu-miR-34c-5p | -0.67 | 2.28E-05 | 1.76E-02 |
| 56219 | Extl1 | mmu-miR-34c-5p | -0.49 | 4.65E-03 | 9.17E-02 |
| 74383 | Ubap2l | mmu-miR-34c-5p | -0.61 | 2.23E-04 | 2.92E-02 |
| 76308 | Rab1b | mmu-miR-34c-5p | 0.57 | 5.99E-04 | 4.06E-02 |
| 109145 | Gins4 | mmu-miR-34c-5p | -0.50 | 3.27E-03 | 7.97E-02 |
| 20525 | Slc2a1 | mmu-miR-34c-5p | -0.56 | 9.03E-04 | 4.74E-02 |
| 235431 | Coro2b | mmu-miR-34c-5p | -0.55 | 1.07E-03 | 5.12E-02 |
| 117109 | Pop5 | mmu-miR-34c-5p | -0.49 | 4.20E-03 | 8.72E-02 |
| 67916 | Ppap2b | mmu-miR-34c-5p | 0.49 | 4.79E-03 | 9.26E-02 |
| 319604 | Fam168a | mmu-miR-34c-5p | 0.51 | 3.13E-03 | 7.84E-02 |
| 18190 | Nrxn2 | mmu-miR-34c-5p | -0.58 | 5.08E-04 | 3.85E-02 |
| 14086 | Fscn1 | mmu-miR-34c-5p | -0.54 | 1.33E-03 | 5.54E-02 |
| 54371 | Chst2 | mmu-miR-34c-5p | -0.63 | 1.23E-04 | 2.51E-02 |
| 52915 | Zmiz2 | mmu-miR-34c-5p | -0.53 | 1.89E-03 | 6.20E-02 |
| 229521 | Syt11 | mmu-miR-34c-5p | 0.63 | 9.57E-05 | 2.34E-02 |
| 71770 | Ap2b1 | mmu-miR-34c-5p | -0.56 | 9.20E-04 | 4.74E-02 |
| 80909 | Gats | mmu-miR-34c-5p | 0.51 | 3.03E-03 | 7.82E-02 |
| 18626 | Per1 | mmu-miR-34c-5p | -0.53 | 1.79E-03 | 6.11E-02 |
| 20779 | Src | mmu-miR-34c-5p | -0.66 | 4.42E-05 | 1.83E-02 |
| 68177 | Ebpl | mmu-miR-34c-5p | -0.61 | 2.21E-04 | 2.92E-02 |
| 52822 | Rufy3 | mmu-miR-34c-5p | 0.56 | 8.83E-04 | 4.72E-02 |
| 68796 | 1110039B18Rik | mmu-miR-34c-5p | -0.49 | 4.50E-03 | 8.95E-02 |
| 69080 | Gmppa | mmu-miR-34c-5p | 0.62 | 1.33E-04 | 2.51E-02 |
| 30948 | Bin1 | mmu-miR-34c-5p | 0.66 | 3.89E-05 | 1.83E-02 |
| 73750 | Whrn | mmu-miR-34c-5p | -0.61 | 2.24E-04 | 2.92E-02 |
| 56395 | Tmem115 | mmu-miR-34c-5p | -0.56 | 8.92E-04 | 4.72E-02 |
| 78465 | 1700084C01Rik | mmu-miR-34c-5p | -0.64 | 9.21E-05 | 2.32E-02 |
| 67941 | Rps27l | mmu-miR-34c-5p | -0.49 | 3.98E-03 | 8.58E-02 |
| 13527 | Dtna | mmu-miR-34c-5p | 0.54 | 1.26E-03 | 5.48E-02 |
| 232791 | Cnot3 | mmu-miR-34c-5p | -0.59 | 4.15E-04 | 3.74E-02 |
| 13885 | Esd | mmu-miR-34c-5p | -0.56 | 8.52E-04 | 4.66E-02 |
| 21968 | Tom1 | mmu-miR-34c-5p | 0.62 | 1.55E-04 | 2.68E-02 |
| 57776 | Ttyh1 | mmu-miR-34c-5p | 0.68 | 2.14E-05 | 1.76E-02 |
| 216881 | Wscd1 | mmu-miR-34c-5p | -0.57 | 6.45E-04 | 4.06E-02 |
| 99237 | Tm9sf4 | mmu-miR-34c-5p | -0.59 | 3.99E-04 | 3.74E-02 |
| 73046 | Glrx5 | mmu-miR-34c-5p | -0.48 | 5.49E-03 | 9.69E-02 |
| 17751 | Mt3 | mmu-miR-34c-5p | -0.50 | 3.96E-03 | 8.58E-02 |
| 27374 | Prmt5 | mmu-miR-34c-5p | 0.60 | 2.66E-04 | 3.11E-02 |
| 74747 | Ddit4 | mmu-miR-34c-5p | -0.48 | 5.80E-03 | 9.91E-02 |
| 18242 | Oat | mmu-miR-34c-5p | 0.49 | 4.38E-03 | 8.85E-02 |
| 50918 | Myadm | mmu-miR-34c-5p | -0.58 | 4.39E-04 | 3.74E-02 |
| 105246 | Brd9 | mmu-miR-34c-5p | 0.53 | 1.89E-03 | 6.20E-02 |
| 20713 | Serpini1 | mmu-miR-34c-5p | 0.58 | 4.46E-04 | 3.74E-02 |
| 15270 | H2afx | mmu-miR-34c-5p | -0.56 | 8.32E-04 | 4.57E-02 |
| 21375 | Tbr1 | mmu-miR-34c-5p | -0.53 | 1.90E-03 | 6.20E-02 |
| 13855 | Epn2 | mmu-miR-34c-5p | 0.48 | 4.94E-03 | 9.32E-02 |
| 76987 | Hdhd2 | mmu-miR-34c-5p | 0.67 | 2.91E-05 | 1.76E-02 |
| 74143 | Opa1 | mmu-miR-34c-5p | 0.51 | 2.79E-03 | 7.52E-02 |
| 331063 | AI987692 | mmu-miR-34c-5p | 0.49 | 4.53E-03 | 9.00E-02 |
| 75751 | Ipo4 | mmu-miR-34c-5p | -0.58 | 4.76E-04 | 3.74E-02 |
| 72018 | Fundc1 | mmu-miR-34c-5p | 0.51 | 3.10E-03 | 7.84E-02 |
| 170750 | Xpnpep1 | mmu-miR-34c-5p | 0.58 | 5.45E-04 | 3.94E-02 |
| 212398 | Frat2 | mmu-miR-34c-5p | -0.52 | 2.24E-03 | 6.67E-02 |
| 59287 | Ncstn | mmu-miR-34c-5p | -0.48 | 4.93E-03 | 9.32E-02 |
| 108083 | Pip4k2b | mmu-miR-34c-5p | -0.58 | 5.24E-04 | 3.85E-02 |
| 11932 | Atp1b2 | mmu-miR-34c-5p | -0.57 | 6.71E-04 | 4.09E-02 |
| 69568 | Vkorc1l1 | mmu-miR-34c-5p | -0.51 | 2.83E-03 | 7.53E-02 |
| 11816 | Apoe | mmu-miR-34c-5p | 0.60 | 3.20E-04 | 3.44E-02 |
| 27370 | Rps26 | mmu-miR-34c-5p | -0.50 | 3.75E-03 | 8.41E-02 |
| 57783 | Tnip1 | mmu-miR-34c-5p | -0.54 | 1.54E-03 | 5.81E-02 |
| 20182 | Rxrb | mmu-miR-34c-5p | 0.61 | 1.92E-04 | 2.91E-02 |
| 66119 | Tomm6 | mmu-miR-34c-5p | -0.50 | 3.21E-03 | 7.91E-02 |
| 77980 | Sbf1 | mmu-miR-34c-5p | 0.59 | 3.29E-04 | 3.46E-02 |
| 100637 | B230342M21Rik | mmu-miR-34c-5p | 0.49 | 4.74E-03 | 9.22E-02 |
| 20338 | Sel1l | mmu-miR-34c-5p | 0.53 | 1.98E-03 | 6.34E-02 |
| 21927 | Tnfaip1 | mmu-miR-34c-5p | 0.57 | 7.42E-04 | 4.28E-02 |
| 407785 | Ndufs6 | mmu-miR-34c-5p | -0.48 | 5.79E-03 | 9.91E-02 |
| 26562 | Ncdn | mmu-miR-34c-5p | -0.58 | 4.53E-04 | 3.74E-02 |
| 69202 | Ptms | mmu-miR-34c-5p | -0.50 | 3.50E-03 | 8.36E-02 |
| 68936 | 1190017O12Rik | mmu-miR-34c-5p | -0.53 | 2.03E-03 | 6.44E-02 |
| 232670 | Tspan33 | mmu-miR-34c-5p | -0.51 | 3.14E-03 | 7.84E-02 |
| 72503 | 2610507B11Rik | mmu-miR-34c-5p | 0.58 | 5.70E-04 | 3.98E-02 |
| 18117 | Cox4nb | mmu-miR-34c-5p | -0.61 | 2.09E-04 | 2.92E-02 |
| 99010 | Lpcat4 | mmu-miR-34c-5p | -0.57 | 6.74E-04 | 4.09E-02 |
| 22232 | Slc35a2 | mmu-miR-34c-5p | -0.52 | 2.26E-03 | 6.68E-02 |
| 22594 | Xrcc1 | mmu-miR-34c-5p | -0.64 | 8.22E-05 | 2.20E-02 |
| 18029 | Nfic | mmu-miR-34c-5p | -0.51 | 3.11E-03 | 7.84E-02 |
| 27366 | Txnl4a | mmu-miR-34c-5p | -0.52 | 2.07E-03 | 6.53E-02 |
| 69072 | Ebna1bp2 | mmu-miR-34c-5p | -0.58 | 4.40E-04 | 3.74E-02 |
| 21761 | Morf4l1 | mmu-miR-34c-5p | 0.55 | 1.18E-03 | 5.32E-02 |
| 19025 | Ctsa | mmu-miR-34c-5p | 0.63 | 1.31E-04 | 2.51E-02 |
| 21807 | Tsc22d1 | mmu-miR-34c-5p | 0.62 | 1.35E-04 | 2.51E-02 |
| 100213 | Rusc2 | mmu-miR-34c-5p | -0.64 | 6.86E-05 | 2.11E-02 |
| 53379 | Hnrnpa2b1 | mmu-miR-34c-5p | 0.68 | 1.91E-05 | 1.76E-02 |
| 104010 | Cdh22 | mmu-miR-34c-5p | -0.48 | 5.79E-03 | 9.91E-02 |
| 432763 | Prr7 | mmu-miR-34c-5p | -0.54 | 1.32E-03 | 5.53E-02 |
| 14683 | Gnas | mmu-miR-34c-5p | 0.61 | 1.81E-04 | 2.91E-02 |
| 69961 | 2810432D09Rik | mmu-miR-34c-5p | -0.55 | 1.09E-03 | 5.15E-02 |
| 229473 | D930015E06Rik | mmu-miR-34c-5p | -0.55 | 1.11E-03 | 5.15E-02 |
| 71310 | Tbc1d9 | mmu-miR-34c-5p | -0.57 | 7.35E-04 | 4.27E-02 |
| 59003 | Maea | mmu-miR-34c-5p | 0.63 | 1.07E-04 | 2.51E-02 |
| 53380 | Psmd10 | mmu-miR-34c-5p | -0.53 | 1.96E-03 | 6.32E-02 |
| 224997 | Dlgap1 | mmu-miR-34c-5p | 0.61 | 2.35E-04 | 2.92E-02 |
| 21664 | Phlda1 | mmu-miR-34c-5p | -0.53 | 1.62E-03 | 5.90E-02 |
| 226646 | Ndufs2 | mmu-miR-34c-5p | 0.54 | 1.40E-03 | 5.62E-02 |
| 77827 | Krba1 | mmu-miR-34c-5p | 0.61 | 1.89E-04 | 2.91E-02 |
| 246696 | Slc25a28 | mmu-miR-34c-5p | 0.49 | 4.88E-03 | 9.32E-02 |
| 12540 | Cdc42 | mmu-miR-34c-5p | 0.48 | 5.79E-03 | 9.91E-02 |
| 235044 | BC018242 | mmu-miR-34c-5p | -0.49 | 4.16E-03 | 8.70E-02 |
| 276770 | Eif5a | mmu-miR-34c-5p | 0.67 | 3.19E-05 | 1.77E-02 |
| 66108 | Ndufa9 | mmu-miR-34c-5p | -0.50 | 3.70E-03 | 8.41E-02 |
| 72416 | Lrpprc | mmu-miR-34c-5p | 0.55 | 1.11E-03 | 5.15E-02 |
| 94232 | Ubqln4 | mmu-miR-34c-5p | -0.56 | 9.17E-04 | 4.74E-02 |
| 67087 | Ctnnbip1 | mmu-miR-34c-5p | -0.66 | 4.52E-05 | 1.83E-02 |
| 319749 | C230078M08Rik | mmu-miR-34c-5p | -0.55 | 1.20E-03 | 5.32E-02 |
| 26874 | Abcd2 | mmu-miR-34c-5p | 0.48 | 5.02E-03 | 9.36E-02 |
| 224143 | Ktelc1 | mmu-miR-34c-5p | 0.55 | 1.20E-03 | 5.32E-02 |
| 76898 | B3gat1 | mmu-miR-34c-5p | -0.51 | 2.69E-03 | 7.39E-02 |
| 15516 | Hsp90ab1 | mmu-miR-34c-5p | 0.59 | 3.84E-04 | 3.70E-02 |
| 105689 | Mycbp2 | mmu-miR-34c-5p | 0.51 | 2.64E-03 | 7.33E-02 |
| 12729 | Clns1a | mmu-miR-34c-5p | 0.53 | 1.78E-03 | 6.11E-02 |
| 106861 | Abhd3 | mmu-miR-34c-5p | 0.52 | 2.29E-03 | 6.76E-02 |
| 56258 | Hnrnph2 | mmu-miR-34c-5p | 0.53 | 1.79E-03 | 6.11E-02 |
| 20692 | Sparc | mmu-miR-34c-5p | -0.54 | 1.41E-03 | 5.63E-02 |
| 18810 | Plec1 | mmu-miR-34c-5p | 0.60 | 2.93E-04 | 3.24E-02 |
| 18642 | Pfkm | mmu-miR-34c-5p | 0.65 | 5.31E-05 | 1.84E-02 |
| 75410 | Wbp7 | mmu-miR-34c-5p | -0.54 | 1.30E-03 | 5.48E-02 |
| 66861 | Dnajc10 | mmu-miR-34c-5p | 0.51 | 2.84E-03 | 7.53E-02 |
| 216869 | Arrb2 | mmu-miR-34c-5p | -0.54 | 1.29E-03 | 5.48E-02 |
| 277939 | C2cd3 | mmu-miR-34c-5p | -0.58 | 5.31E-04 | 3.87E-02 |
| 16467 | Atcay | mmu-miR-34c-5p | -0.50 | 3.25E-03 | 7.94E-02 |
| 243819 | Saps1 | mmu-miR-34c-5p | -0.58 | 5.71E-04 | 3.98E-02 |
| 241943 | BC050811 | mmu-miR-34c-5p | -0.52 | 2.14E-03 | 6.66E-02 |
| 23825 | Banf1 | mmu-miR-34c-5p | -0.48 | 5.74E-03 | 9.89E-02 |
| 66878 | Riok3 | mmu-miR-34c-5p | 0.54 | 1.60E-03 | 5.90E-02 |
| 11518 | Add1 | mmu-miR-34c-5p | 0.67 | 2.77E-05 | 1.76E-02 |
| 211712 | Pcdh9 | mmu-miR-34c-5p | 0.48 | 5.38E-03 | 9.63E-02 |
| 76687 | Spcs3 | mmu-miR-34c-5p | 0.53 | 1.80E-03 | 6.11E-02 |
| 67178 | Zmat5 | mmu-miR-34c-5p | -0.50 | 3.70E-03 | 8.41E-02 |
| 319477 | 6030419C18Rik | mmu-miR-34c-5p | -0.62 | 1.37E-04 | 2.51E-02 |
| 18194 | Nsdhl | mmu-miR-34c-5p | 0.60 | 2.79E-04 | 3.22E-02 |
| 11772 | Ap2a2 | mmu-miR-34c-5p | -0.57 | 5.98E-04 | 4.06E-02 |
| 245688 | Rbbp7 | mmu-miR-34c-5p | 0.57 | 6.04E-04 | 4.06E-02 |
| 100046136 | LOC100046136 | mmu-miR-34c-5p | 0.53 | 1.74E-03 | 6.11E-02 |
| 74192 | Arpc5l | mmu-miR-34c-5p | -0.53 | 1.87E-03 | 6.17E-02 |
| 19261 | Sirpa | mmu-miR-34c-5p | 0.62 | 1.51E-04 | 2.66E-02 |
| 236539 | Phgdh | mmu-miR-34c-5p | 0.55 | 1.06E-03 | 5.09E-02 |
| 12153 | Bmp1 | mmu-miR-34c-5p | 0.57 | 7.29E-04 | 4.27E-02 |
| 22134 | Tgoln1 | mmu-miR-34c-5p | 0.53 | 1.73E-03 | 6.11E-02 |
| 320078 | Olfml2b | mmu-miR-34c-5p | -0.52 | 2.18E-03 | 6.67E-02 |
| 217335 | Fbf1 | mmu-miR-34c-5p | -0.49 | 4.05E-03 | 8.67E-02 |
| 22404 | Wiz | mmu-miR-34c-5p | -0.59 | 4.29E-04 | 3.74E-02 |
| 74322 | Cxxc1 | mmu-miR-34c-5p | -0.56 | 8.04E-04 | 4.51E-02 |
| 23945 | Mgll | mmu-miR-34c-5p | 0.51 | 2.80E-03 | 7.52E-02 |
| 97761 | Sgsm2 | mmu-miR-34c-5p | -0.54 | 1.37E-03 | 5.56E-02 |
| 108058 | Camk2d | mmu-miR-34c-5p | 0.49 | 4.29E-03 | 8.81E-02 |
| 17965 | Nbl1 | mmu-miR-34c-5p | 0.68 | 1.87E-05 | 1.76E-02 |
| 53324 | Nptx2 | mmu-miR-34c-5p | -0.50 | 3.73E-03 | 8.41E-02 |
| 100608 | Noc4l | mmu-miR-34c-5p | -0.54 | 1.35E-03 | 5.56E-02 |
| 212127 | 2810046L04Rik | mmu-miR-34c-5p | -0.50 | 3.92E-03 | 8.58E-02 |
| 70231 | Gorasp2 | mmu-miR-34c-5p | 0.67 | 2.97E-05 | 1.76E-02 |
| 56876 | Nelf | mmu-miR-34c-5p | 0.59 | 3.77E-04 | 3.68E-02 |
| 218811 | Sec24c | mmu-miR-34c-5p | -0.49 | 4.38E-03 | 8.85E-02 |
| 667977 | EG667977 | mmu-miR-34c-5p | 0.59 | 4.13E-04 | 3.74E-02 |
| 234730 | Fuk | mmu-miR-34c-5p | -0.57 | 7.22E-04 | 4.27E-02 |
| 11545 | Parp1 | mmu-miR-34c-5p | -0.72 | 4.18E-06 | 1.74E-02 |
| 104444 | Rexo2 | mmu-miR-34c-5p | -0.49 | 4.15E-03 | 8.70E-02 |
| 77038 | Arfgap2 | mmu-miR-34c-5p | -0.50 | 3.73E-03 | 8.41E-02 |
| 216739 | Acsl6 | mmu-miR-34c-5p | 0.61 | 2.04E-04 | 2.92E-02 |
| 74255 | Smu1 | mmu-miR-34c-5p | 0.53 | 1.76E-03 | 6.11E-02 |
| 13819 | Epas1 | mmu-miR-34c-5p | 0.62 | 1.39E-04 | 2.51E-02 |
| 19245 | Ptp4a3 | mmu-miR-34c-5p | -0.52 | 2.19E-03 | 6.67E-02 |
| 15114 | Hap1 | mmu-miR-34c-5p | 0.50 | 3.41E-03 | 8.23E-02 |
| 66513 | Map3k7ip1 | mmu-miR-34c-5p | -0.61 | 2.23E-04 | 2.92E-02 |
| 15361 | Hmga1 | mmu-miR-34c-5p | -0.52 | 2.36E-03 | 6.82E-02 |
| 227801 | Dennd1a | mmu-miR-34c-5p | -0.50 | 3.74E-03 | 8.41E-02 |
| 229615 | Pias3 | mmu-miR-34c-5p | -0.48 | 5.21E-03 | 9.42E-02 |
| 75785 | Klhl24 | mmu-miR-34c-5p | 0.58 | 5.20E-04 | 3.85E-02 |
| 21858 | Timp2 | mmu-miR-34c-5p | -0.56 | 8.81E-04 | 4.72E-02 |
| 57435 | S3-12 | mmu-miR-34c-5p | -0.54 | 1.47E-03 | 5.73E-02 |
| 19055 | Ppp3ca | mmu-miR-34c-5p | 0.49 | 4.17E-03 | 8.70E-02 |
| 66359 | 2310005N03Rik | mmu-miR-34c-5p | -0.64 | 7.55E-05 | 2.16E-02 |
| 329015 | Atg2a | mmu-miR-34c-5p | -0.51 | 3.02E-03 | 7.80E-02 |
| 108155 | Ogt | mmu-miR-34c-5p | 0.55 | 1.24E-03 | 5.44E-02 |
| 20887 | Sult1a1 | mmu-miR-34c-5p | -0.55 | 1.20E-03 | 5.32E-02 |
| 13424 | Dync1h1 | mmu-miR-34c-5p | -0.50 | 3.59E-03 | 8.41E-02 |
| 17388 | Mmp15 | mmu-miR-34c-5p | -0.51 | 3.14E-03 | 7.84E-02 |
| 19042 | Ppm1a | mmu-miR-34c-5p | 0.48 | 5.44E-03 | 9.66E-02 |
| 665250 | LOC665250 | mmu-miR-34c-5p | -0.51 | 3.07E-03 | 7.84E-02 |
| 56149 | Grasp | mmu-miR-34c-5p | 0.58 | 5.16E-04 | 3.85E-02 |
| 101869 | Unc45a | mmu-miR-34c-5p | -0.56 | 8.81E-04 | 4.72E-02 |
| 228608 | Smox | mmu-miR-34c-5p | -0.50 | 3.79E-03 | 8.43E-02 |
| 30937 | Lmcd1 | mmu-miR-34c-5p | 0.57 | 6.46E-04 | 4.06E-02 |
| 94180 | Acsbg1 | mmu-miR-34c-5p | 0.53 | 1.61E-03 | 5.90E-02 |
| 71777 | Ing3 | mmu-miR-34c-5p | 0.51 | 3.00E-03 | 7.80E-02 |
| 545007 | ENSMUSG00000068790 | mmu-miR-34c-5p | 0.48 | 5.70E-03 | 9.84E-02 |
| 78308 | Gpr108 | mmu-miR-34c-5p | 0.56 | 9.60E-04 | 4.80E-02 |
| 13628 | Eef1a2 | mmu-miR-34c-5p | 0.61 | 2.36E-04 | 2.92E-02 |
| 71947 | 2310067B10Rik | mmu-miR-34c-5p | -0.57 | 7.48E-04 | 4.28E-02 |
| 14678 | Gnai2 | mmu-miR-34c-5p | -0.56 | 9.22E-04 | 4.74E-02 |
| 66836 | 0610006I08Rik | mmu-miR-34c-5p | -0.53 | 1.90E-03 | 6.20E-02 |
| 228836 | Dlgap4 | mmu-miR-34c-5p | 0.48 | 4.99E-03 | 9.35E-02 |
| 14911 | Thumpd3 | mmu-miR-34c-5p | 0.54 | 1.48E-03 | 5.73E-02 |
| 100046744 | LOC100046744 | mmu-miR-34c-5p | -0.65 | 6.56E-05 | 2.09E-02 |
| 22283 | Ush2a | mmu-miR-34c-5p | -0.59 | 3.94E-04 | 3.74E-02 |
| 12660 | Chka | mmu-miR-34c-5p | 0.59 | 3.51E-04 | 3.56E-02 |
| 70439 | Taf15 | mmu-miR-34c-5p | -0.49 | 4.45E-03 | 8.92E-02 |
| 229791 | D3Bwg0562e | mmu-miR-34c-5p | 0.54 | 1.57E-03 | 5.89E-02 |
| 320982 | Arl4c | mmu-miR-34c-5p | 0.53 | 1.84E-03 | 6.15E-02 |
| 11475 | Acta2 | mmu-miR-34c-5p | 0.56 | 8.88E-04 | 4.72E-02 |
| 20273 | Scn8a | mmu-miR-34c-5p | 0.51 | 2.79E-03 | 7.52E-02 |
| 71916 | Dus4l | mmu-miR-34c-5p | -0.62 | 1.74E-04 | 2.90E-02 |
| 269397 | Ss18l1 | mmu-miR-34c-5p | 0.58 | 4.48E-04 | 3.74E-02 |
| 68499 | Mrpl53 | mmu-miR-34c-5p | -0.48 | 5.07E-03 | 9.40E-02 |
| 66830 | Nacc1 | mmu-miR-34c-5p | -0.49 | 4.67E-03 | 9.17E-02 |
| 228355 | Madd | mmu-miR-34c-5p | 0.56 | 9.36E-04 | 4.74E-02 |
| 230996 | 9430015G10Rik | mmu-miR-34c-5p | -0.52 | 2.21E-03 | 6.67E-02 |
| 211548 | Nomo1 | mmu-miR-34c-5p | -0.49 | 4.06E-03 | 8.67E-02 |
| 69780 | Smap2 | mmu-miR-34c-5p | -0.58 | 5.06E-04 | 3.85E-02 |
| 75533 | Nme5 | mmu-miR-34c-5p | -0.48 | 4.96E-03 | 9.32E-02 |
| 20910 | Stxbp1 | mmu-miR-34c-5p | 0.68 | 2.19E-05 | 1.76E-02 |
| 11737 | Anp32a | mmu-miR-34c-5p | -0.64 | 8.51E-05 | 2.21E-02 |
| 19775 | Xpr1 | mmu-miR-34c-5p | -0.55 | 1.00E-03 | 4.93E-02 |
| 100737 | Dcun1d4 | mmu-miR-34c-5p | 0.57 | 6.08E-04 | 4.06E-02 |
| 50781 | Dkk3 | mmu-miR-34c-5p | 0.57 | 7.31E-04 | 4.27E-02 |
| 83486 | Rbm5 | mmu-miR-34c-5p | 0.51 | 3.09E-03 | 7.84E-02 |
| 73826 | Poldip3 | mmu-miR-34c-5p | 0.61 | 2.10E-04 | 2.92E-02 |
| 100046802 | LOC100046802 | mmu-miR-34c-5p | -0.52 | 2.36E-03 | 6.82E-02 |
| 233033 | Samd4b | mmu-miR-34c-5p | -0.48 | 5.17E-03 | 9.42E-02 |
| 57295 | Icmt | mmu-miR-34c-5p | -0.49 | 4.34E-03 | 8.81E-02 |
| 69837 | Pcgf1 | mmu-miR-34c-5p | -0.49 | 4.63E-03 | 9.16E-02 |
| 208171 | Tmprss7 | mmu-miR-34c-5p | -0.51 | 2.91E-03 | 7.68E-02 |
| 16579 | Kifap3 | mmu-miR-34c-5p | 0.51 | 2.85E-03 | 7.53E-02 |
| 116733 | Vps4a | mmu-miR-34c-5p | 0.51 | 2.67E-03 | 7.39E-02 |
| 16906 | Lmnb1 | mmu-miR-34c-5p | -0.69 | 1.12E-05 | 1.76E-02 |
| 269615 | Plch2 | mmu-miR-34c-5p | -0.54 | 1.45E-03 | 5.71E-02 |
| 104318 | Csnk1d | mmu-miR-34c-5p | 0.50 | 3.57E-03 | 8.41E-02 |
| 68112 | Sdccag3 | mmu-miR-34c-5p | 0.48 | 5.22E-03 | 9.42E-02 |
| 18710 | Pik3r3 | mmu-miR-34c-5p | 0.50 | 3.75E-03 | 8.41E-02 |
| 67420 | Far1 | mmu-miR-34c-5p | 0.66 | 4.74E-05 | 1.83E-02 |
| 19305 | Pex5 | mmu-miR-34c-5p | -0.57 | 6.31E-04 | 4.06E-02 |
| 108075 | Ltbp4 | mmu-miR-34c-5p | 0.61 | 1.83E-04 | 2.91E-02 |
| 20933 | Med22 | mmu-miR-34c-5p | -0.51 | 3.09E-03 | 7.84E-02 |
| 74302 | Mtmr3 | mmu-miR-34c-5p | 0.50 | 3.38E-03 | 8.20E-02 |
| 231670 | Fbxo21 | mmu-miR-34c-5p | -0.54 | 1.33E-03 | 5.54E-02 |
| 17168 | Mare | mmu-miR-34c-5p | 0.50 | 3.59E-03 | 8.41E-02 |
| 68229 | AI846148 | mmu-miR-34c-5p | -0.52 | 2.51E-03 | 7.12E-02 |
| 66366 | Ergic3 | mmu-miR-34c-5p | 0.58 | 5.52E-04 | 3.95E-02 |
| 228368 | Slc35c1 | mmu-miR-34c-5p | -0.48 | 5.13E-03 | 9.42E-02 |
| 235661 | Dync1li1 | mmu-miR-34c-5p | 0.52 | 2.11E-03 | 6.59E-02 |
| 22057 | Tob1 | mmu-miR-34c-5p | -0.67 | 2.70E-05 | 1.76E-02 |
| 56316 | Ggcx | mmu-miR-34c-5p | -0.53 | 1.70E-03 | 6.11E-02 |
| 107035 | Fbxo38 | mmu-miR-34c-5p | 0.48 | 5.11E-03 | 9.42E-02 |
| 16202 | Ilk | mmu-miR-34c-5p | -0.56 | 8.29E-04 | 4.57E-02 |
| 14661 | Lgsn | mmu-miR-34c-5p | 0.61 | 1.96E-04 | 2.91E-02 |
| 225392 | Rell2 | mmu-miR-34c-5p | 0.50 | 3.76E-03 | 8.42E-02 |
| 109754 | Cyb5r3 | mmu-miR-34c-5p | -0.54 | 1.50E-03 | 5.78E-02 |
| 234854 | Cdk10 | mmu-miR-34c-5p | 0.58 | 4.40E-04 | 3.74E-02 |
| 19060 | Ppp5c | mmu-miR-34c-5p | 0.68 | 2.07E-05 | 1.76E-02 |
| 52040 | Ppp1r10 | mmu-miR-34c-5p | -0.51 | 2.63E-03 | 7.33E-02 |
| 217732 | 2310044G17Rik | mmu-miR-34c-5p | 0.55 | 1.16E-03 | 5.32E-02 |
| 319601 | Zfp653 | mmu-miR-34c-5p | -0.55 | 1.21E-03 | 5.32E-02 |
| 17196 | Mbp | mmu-miR-34c-5p | 0.50 | 3.82E-03 | 8.45E-02 |
| 19654 | Rbm6 | mmu-miR-34c-5p | 0.49 | 4.48E-03 | 8.93E-02 |
| 21767 | Tex264 | mmu-miR-34c-5p | 0.51 | 3.18E-03 | 7.88E-02 |
| 228998 | Arfgap1 | mmu-miR-34c-5p | 0.52 | 2.06E-03 | 6.52E-02 |
| 20384 | Sfrs5 | mmu-miR-34c-5p | 0.55 | 1.20E-03 | 5.32E-02 |
| 16796 | Lasp1 | mmu-miR-34c-5p | -0.54 | 1.44E-03 | 5.71E-02 |
| 101471 | Phrf1 | mmu-miR-34c-5p | -0.50 | 3.83E-03 | 8.45E-02 |
| 14229 | Fkbp5 | mmu-miR-34c-5p | -0.54 | 1.42E-03 | 5.66E-02 |
| 19935 | Mrpl23 | mmu-miR-34c-5p | -0.50 | 3.88E-03 | 8.54E-02 |
| 16792 | Laptm5 | mmu-miR-34c-5p | 0.52 | 2.34E-03 | 6.82E-02 |
| 219022 | Ttc5 | mmu-miR-34c-5p | 0.61 | 2.30E-04 | 2.92E-02 |
| 75627 | Snapc1 | mmu-miR-34c-5p | -0.55 | 1.12E-03 | 5.18E-02 |
| 240753 | Plekha6 | mmu-miR-34c-5p | 0.52 | 2.42E-03 | 6.94E-02 |
| 214572 | Prmt7 | mmu-miR-34c-5p | 0.58 | 4.78E-04 | 3.74E-02 |
| 108124 | Napa | mmu-miR-34c-5p | 0.57 | 6.75E-04 | 4.09E-02 |
| 328949 | Mcc | mmu-miR-34c-5p | -0.59 | 4.07E-04 | 3.74E-02 |
| 66399 | Tsfm | mmu-miR-34c-5p | -0.52 | 2.53E-03 | 7.12E-02 |
| 100048622 | LOC100048622 | mmu-miR-34c-5p | 0.49 | 4.24E-03 | 8.73E-02 |
| 574404 | OTTMUSG00000017677 | mmu-miR-34c-5p | -0.51 | 2.84E-03 | 7.53E-02 |
| 70025 | Acot7 | mmu-miR-34c-5p | 0.63 | 1.20E-04 | 2.51E-02 |
| 70510 | Rnf167 | mmu-miR-34c-5p | 0.54 | 1.36E-03 | 5.56E-02 |
| 68585 | Rtn4 | mmu-miR-34c-5p | 0.52 | 2.40E-03 | 6.88E-02 |
| 14232 | Fkbp8 | mmu-miR-34c-5p | 0.56 | 9.24E-04 | 4.74E-02 |
| 69106 | Stoml1 | mmu-miR-34c-5p | 0.52 | 2.10E-03 | 6.58E-02 |
| 227644 | Snapc4 | mmu-miR-34c-5p | -0.56 | 9.52E-04 | 4.79E-02 |
| 50767 | Pnpla6 | mmu-miR-34c-5p | 0.48 | 5.67E-03 | 9.84E-02 |
| 67345 | Herc4 | mmu-miR-34c-5p | 0.57 | 6.38E-04 | 4.06E-02 |
| 74763 | Nat15 | mmu-miR-34c-5p | 0.70 | 8.34E-06 | 1.76E-02 |
| 100044363 | LOC100044363 | mmu-miR-34c-5p | 0.50 | 3.71E-03 | 8.41E-02 |
| 14168 | Fgf13 | mmu-miR-34c-5p | 0.50 | 3.66E-03 | 8.41E-02 |
| 233900 | Rnf40 | mmu-miR-34c-5p | -0.53 | 1.78E-03 | 6.11E-02 |
| 12321 | Calu | mmu-miR-34c-5p | 0.57 | 7.11E-04 | 4.25E-02 |
| 50997 | Mpp2 | mmu-miR-34c-5p | -0.49 | 4.34E-03 | 8.81E-02 |
| 234595 | BC031853 | mmu-miR-34c-5p | -0.57 | 6.39E-04 | 4.06E-02 |
| 212518 | Sprn | mmu-miR-34c-5p | 0.51 | 2.78E-03 | 7.52E-02 |
| 30853 | Mlf2 | mmu-miR-34c-5p | 0.52 | 2.15E-03 | 6.66E-02 |
| 100047184 | LOC100047184 | mmu-miR-34c-5p | -0.53 | 1.95E-03 | 6.29E-02 |
| 14312 | Brd2 | mmu-miR-34c-5p | -0.50 | 3.53E-03 | 8.41E-02 |
| 15510 | Hspd1 | mmu-miR-34c-5p | 0.52 | 2.22E-03 | 6.67E-02 |
| 69942 | Rnf113a1 | mmu-miR-34c-5p | -0.50 | 3.74E-03 | 8.41E-02 |
| 56323 | Dnajb5 | mmu-miR-34c-5p | -0.56 | 8.12E-04 | 4.52E-02 |
| 64453 | Suhw2 | mmu-miR-34c-5p | -0.50 | 3.22E-03 | 7.91E-02 |
| 21429 | Ubtf | mmu-miR-34c-5p | -0.52 | 2.51E-03 | 7.12E-02 |
| 236920 | Stard8 | mmu-miR-34c-5p | -0.50 | 3.73E-03 | 8.41E-02 |
| 329559 | Zfp335 | mmu-miR-34c-5p | -0.58 | 5.71E-04 | 3.98E-02 |
| 66433 | Chchd7 | mmu-miR-34c-5p | -0.49 | 4.13E-03 | 8.70E-02 |
| 14131 | Fcgr3 | mmu-miR-34c-5p | -0.60 | 3.23E-04 | 3.44E-02 |
| 56421 | Pfkp | mmu-miR-34c-5p | 0.58 | 4.68E-04 | 3.74E-02 |
| 56786 | Tmem9b | mmu-miR-34c-5p | 0.51 | 2.63E-03 | 7.33E-02 |
| 66743 | Rnf220 | mmu-miR-34c-5p | 0.58 | 4.98E-04 | 3.85E-02 |
| 73103 | 3110009E18Rik | mmu-miR-34c-5p | -0.54 | 1.60E-03 | 5.90E-02 |
| 57265 | Fzd2 | mmu-miR-34c-5p | -0.48 | 5.49E-03 | 9.69E-02 |
| 14670 | Gnal1 | mmu-miR-34c-5p | -0.48 | 5.64E-03 | 9.81E-02 |
| 54376 | Cacng3 | mmu-miR-34c-5p | 0.50 | 3.94E-03 | 8.58E-02 |
| 504193 | Npcd | mmu-miR-34c-5p | 0.48 | 5.12E-03 | 9.42E-02 |
| 23882 | Gadd45g | mmu-miR-34c-5p | -0.51 | 2.68E-03 | 7.39E-02 |
| 52815 | Ldhd | mmu-miR-34c-5p | -0.51 | 2.80E-03 | 7.52E-02 |
| 100045617 | LOC100045617 | mmu-miR-34c-5p | 0.59 | 3.76E-04 | 3.68E-02 |
| 54614 | Prpf40b | mmu-miR-34c-5p | -0.49 | 4.60E-03 | 9.11E-02 |
| 212627 | Prpsap2 | mmu-miR-34c-5p | 0.64 | 7.14E-05 | 2.12E-02 |
| 19385 | Ranbp1 | mmu-miR-34c-5p | -0.49 | 4.79E-03 | 9.26E-02 |
| 23928 | Lamc3 | mmu-miR-34c-5p | -0.60 | 2.97E-04 | 3.24E-02 |
| 241846 | Lsm14b | mmu-miR-34c-5p | -0.54 | 1.55E-03 | 5.82E-02 |
| 229504 | Isg20l2 | mmu-miR-34c-5p | -0.50 | 3.45E-03 | 8.30E-02 |
| 23991 | Cib1 | mmu-miR-34c-5p | -0.51 | 3.20E-03 | 7.91E-02 |
| 20351 | Sema4a | mmu-miR-34c-5p | 0.53 | 1.85E-03 | 6.15E-02 |
| 67887 | Tmem66 | mmu-miR-34c-5p | 0.48 | 4.93E-03 | 9.32E-02 |
| 57437 | Golga7 | mmu-miR-34c-5p | 0.60 | 2.84E-04 | 3.23E-02 |
| 21885 | Tle1 | mmu-miR-34c-5p | -0.52 | 2.25E-03 | 6.67E-02 |
| 20810 | Srm | mmu-miR-34c-5p | 0.52 | 2.36E-03 | 6.82E-02 |
| 26875 | Pclo | mmu-miR-34c-5p | 0.51 | 2.75E-03 | 7.52E-02 |
| 53621 | Cnot4 | mmu-miR-34c-5p | 0.50 | 3.67E-03 | 8.41E-02 |
| 15275 | Hk1 | mmu-miR-34c-5p | 0.53 | 1.69E-03 | 6.11E-02 |
| 74334 | Ranbp10 | mmu-miR-34c-5p | -0.49 | 4.00E-03 | 8.59E-02 |
| 99151 | Cercam | mmu-miR-34c-5p | 0.54 | 1.30E-03 | 5.48E-02 |
| 53627 | Porcn | mmu-miR-34c-5p | 0.57 | 6.63E-04 | 4.09E-02 |
| 13123 | Cyp7b1 | mmu-miR-34c-5p | -0.49 | 4.90E-03 | 9.32E-02 |
| 108115 | Slco4a1 | mmu-miR-34c-5p | -0.48 | 4.96E-03 | 9.32E-02 |
| 20536 | Slc4a3 | mmu-miR-34c-5p | 0.53 | 1.94E-03 | 6.29E-02 |
| 328424 | Kcnrg | mmu-miR-34c-5p | -0.49 | 4.31E-03 | 8.81E-02 |
| 19349 | Rab7 | mmu-miR-34c-5p | 0.48 | 5.42E-03 | 9.64E-02 |
| 57170 | Dolpp1 | mmu-miR-34c-5p | 0.48 | 5.55E-03 | 9.72E-02 |
| 22241 | Ulk1 | mmu-miR-34c-5p | -0.53 | 1.78E-03 | 6.11E-02 |
| 67604 | 1110007L15Rik | mmu-miR-34c-5p | 0.55 | 1.00E-03 | 4.93E-02 |
| 56505 | Ruvbl1 | mmu-miR-34c-5p | 0.52 | 2.34E-03 | 6.82E-02 |
| 66910 | Tmem107 | mmu-miR-34c-5p | -0.49 | 4.46E-03 | 8.92E-02 |
| 76936 | Hnrpm | mmu-miR-34c-5p | -0.52 | 2.49E-03 | 7.11E-02 |
| 230857 | Ece1 | mmu-miR-34c-5p | -0.48 | 5.20E-03 | 9.42E-02 |
| 218035 | Vps41 | mmu-miR-34c-5p | 0.53 | 1.73E-03 | 6.11E-02 |
| 103711 | Pnpo | mmu-miR-34c-5p | 0.56 | 9.72E-04 | 4.83E-02 |
| 329777 | Pigk | mmu-miR-34c-5p | 0.55 | 1.10E-03 | 5.15E-02 |
| 100047935 | LOC100047935 | mmu-miR-34c-5p | 0.58 | 4.46E-04 | 3.74E-02 |
| 72029 | Cnpy3 | mmu-miR-34c-5p | 0.61 | 1.89E-04 | 2.91E-02 |
| 27041 | G3bp1 | mmu-miR-34c-5p | -0.48 | 5.07E-03 | 9.40E-02 |
| 22275 | Urod | mmu-miR-34c-5p | -0.50 | 3.62E-03 | 8.41E-02 |
| 66733 | Kcng4 | mmu-miR-34c-5p | -0.48 | 5.83E-03 | 9.93E-02 |
| 106840 | Unc119b | mmu-miR-34c-5p | -0.58 | 4.60E-04 | 3.74E-02 |
| 30930 | Vps26a | mmu-miR-34c-5p | 0.53 | 1.70E-03 | 6.11E-02 |
| 105239 | Rnf44 | mmu-miR-34c-5p | -0.49 | 3.98E-03 | 8.58E-02 |
| 19330 | Rab18 | mmu-miR-34c-5p | 0.53 | 1.98E-03 | 6.34E-02 |
| 78339 | Ttyh3 | mmu-miR-34c-5p | -0.49 | 4.17E-03 | 8.70E-02 |
| 15356 | Hmgcl | mmu-miR-34c-5p | 0.51 | 2.81E-03 | 7.52E-02 |
| 59048 | C1galt1c1 | mmu-miR-34c-5p | -0.53 | 1.81E-03 | 6.11E-02 |
| 29871 | Scmh1 | mmu-miR-34c-5p | -0.48 | 5.37E-03 | 9.62E-02 |
| 26949 | Vat1 | mmu-miR-34c-5p | -0.51 | 2.95E-03 | 7.72E-02 |
| 80906 | Kcnip2 | mmu-miR-34c-5p | 0.48 | 5.69E-03 | 9.84E-02 |
| 231571 | Rpap2 | mmu-miR-34c-5p | 0.57 | 6.33E-04 | 4.06E-02 |
| 53612 | Vti1b | mmu-miR-34c-5p | 0.49 | 4.44E-03 | 8.92E-02 |
| 217837 | Itpk1 | mmu-miR-34c-5p | 0.49 | 4.14E-03 | 8.70E-02 |
| 93685 | Entpd7 | mmu-miR-34c-5p | 0.48 | 5.70E-03 | 9.84E-02 |
| 66515 | Cul7 | mmu-miR-34c-5p | 0.49 | 4.83E-03 | 9.29E-02 |
| 12048 | Bcl2l1 | mmu-miR-34c-5p | 0.49 | 4.30E-03 | 8.81E-02 |
| 22436 | Xdh | mmu-miR-34c-5p | -0.48 | 5.23E-03 | 9.42E-02 |
| 30926 | Txnl2 | mmu-miR-34c-5p | -0.49 | 4.83E-03 | 9.29E-02 |
| 16524 | Kcnj9 | mmu-miR-34c-5p | 0.62 | 1.70E-04 | 2.88E-02 |
| 112407 | Egln3 | mmu-miR-34c-5p | -0.52 | 2.18E-03 | 6.67E-02 |
| 97827 | Exdl2 | mmu-miR-34c-5p | 0.49 | 3.97E-03 | 8.58E-02 |
| 216874 | Camta2 | mmu-miR-34c-5p | 0.48 | 5.01E-03 | 9.36E-02 |
| 14548 | Mrps33 | mmu-miR-34c-5p | -0.50 | 3.96E-03 | 8.58E-02 |
| 67529 | Fgfr1op2 | mmu-miR-34c-5p | 0.50 | 3.59E-03 | 8.41E-02 |
| 20510 | Slc1a1 | mmu-miR-34c-5p | -0.48 | 5.20E-03 | 9.42E-02 |
| 14299 | Freq | mmu-miR-34c-5p | -0.51 | 3.12E-03 | 7.84E-02 |
| 102657 | Cd276 | mmu-miR-34c-5p | -0.58 | 5.10E-04 | 3.85E-02 |
| 57370 | B4galt3 | mmu-miR-34c-5p | 0.48 | 5.50E-03 | 9.69E-02 |
| 56807 | Scamp5 | mmu-miR-34c-5p | 0.53 | 1.85E-03 | 6.15E-02 |
| 30934 | Tor1b | mmu-miR-34c-5p | -0.50 | 3.24E-03 | 7.94E-02 |
| 69698 | 2310046K01Rik | mmu-miR-34c-5p | -0.62 | 1.31E-04 | 2.51E-02 |
| 66940 | Scotin | mmu-miR-34c-5p | 0.51 | 3.14E-03 | 7.84E-02 |
| 17005 | Ltk | mmu-miR-34c-5p | 0.56 | 9.30E-04 | 4.74E-02 |
| 16784 | Lamp2 | mmu-miR-34c-5p | 0.55 | 1.09E-03 | 5.15E-02 |
| 68045 | 2700060E02Rik | mmu-miR-34c-5p | -0.54 | 1.53E-03 | 5.81E-02 |
| 11993 | Aup1 | mmu-miR-34c-5p | 0.54 | 1.48E-03 | 5.73E-02 |
| 231997 | Fkbp14 | mmu-miR-34c-5p | 0.51 | 2.59E-03 | 7.27E-02 |
| 11769 | Ap1s1 | mmu-miR-34c-5p | 0.50 | 3.46E-03 | 8.30E-02 |
| 21454 | Tcp1 | mmu-miR-34c-5p | 0.49 | 4.24E-03 | 8.73E-02 |
| 22327 | Vbp1 | mmu-miR-34c-5p | -0.49 | 4.23E-03 | 8.73E-02 |
| 118452 | Baalc | mmu-miR-34c-5p | 0.48 | 5.03E-03 | 9.37E-02 |
| 56412 | 2610024G14Rik | mmu-miR-34c-5p | -0.50 | 3.75E-03 | 8.41E-02 |
| 26403 | Map3k11 | mmu-miR-34c-5p | -0.52 | 2.24E-03 | 6.67E-02 |
| 217980 | Larp5 | mmu-miR-34c-5p | 0.53 | 1.61E-03 | 5.90E-02 |
| 27364 | Srr | mmu-miR-34c-5p | 0.58 | 4.65E-04 | 3.74E-02 |
| 22223 | Uchl1 | mmu-miR-34c-5p | 0.48 | 5.15E-03 | 9.42E-02 |
| 224907 | Dus3l | mmu-miR-34c-5p | 0.54 | 1.51E-03 | 5.79E-02 |
| 192786 | Rapgef6 | mmu-miR-34c-5p | 0.49 | 4.86E-03 | 9.32E-02 |
| 210044 | Adcy2 | mmu-miR-34c-5p | 0.57 | 6.40E-04 | 4.06E-02 |
| 109689 | Arrb1 | mmu-miR-34c-5p | -0.54 | 1.53E-03 | 5.81E-02 |
| 27355 | X99384 | mmu-miR-34c-5p | -0.51 | 3.11E-03 | 7.84E-02 |
| 100044862 | LOC100044862 | mmu-miR-34c-5p | 0.56 | 7.95E-04 | 4.49E-02 |
| 74781 | Wipi2 | mmu-miR-34c-5p | 0.49 | 4.71E-03 | 9.22E-02 |
| 231148 | Ablim2 | mmu-miR-34c-5p | 0.51 | 2.76E-03 | 7.52E-02 |
| 76411 | 1700019E19Rik | mmu-miR-34c-5p | -0.49 | 4.11E-03 | 8.70E-02 |
| 29812 | Ndrg3 | mmu-miR-34c-5p | 0.50 | 3.64E-03 | 8.41E-02 |
| 11981 | Atp9a | mmu-miR-34c-5p | 0.59 | 3.69E-04 | 3.68E-02 |
| 20091 | Rps3a | mmu-miR-34c-5p | 0.48 | 5.25E-03 | 9.44E-02 |
| 26950 | Vsnl1 | mmu-miR-34c-5p | 0.53 | 1.80E-03 | 6.11E-02 |
| 20586 | Smarca4 | mmu-miR-34c-5p | -0.54 | 1.30E-03 | 5.48E-02 |
| 66701 | Spryd4 | mmu-miR-34c-5p | -0.52 | 2.23E-03 | 6.67E-02 |
| 72895 | Setd5 | mmu-miR-34c-5p | -0.48 | 5.57E-03 | 9.73E-02 |
| 27643 | Ubl4 | mmu-miR-34c-5p | 0.65 | 5.26E-05 | 1.84E-02 |
| 12363 | Casp4 | mmu-miR-34c-5p | -0.49 | 4.67E-03 | 9.17E-02 |
| 11773 | Ap2m1 | mmu-miR-34c-5p | 0.48 | 5.18E-03 | 9.42E-02 |
| 107173 | Gpr137 | mmu-miR-34c-5p | 0.48 | 5.40E-03 | 9.63E-02 |
| 16918 | Mycl1 | mmu-miR-34c-5p | -0.49 | 4.72E-03 | 9.22E-02 |
| 67808 | Tprgl | mmu-miR-34c-5p | 0.51 | 3.01E-03 | 7.80E-02 |
| 28040 | D6Wsu163e | mmu-miR-34c-5p | 0.49 | 4.45E-03 | 8.92E-02 |
| 24000 | Ptpn21 | mmu-miR-34c-5p | -0.48 | 5.61E-03 | 9.78E-02 |
| 320213 | Senp5 | mmu-miR-34c-5p | 0.50 | 3.41E-03 | 8.23E-02 |
| 110058 | Syt17 | mmu-miR-34c-5p | 0.48 | 4.96E-03 | 9.32E-02 |
| 56085 | Ubqln1 | mmu-miR-34c-5p | 0.51 | 2.95E-03 | 7.72E-02 |
| 15360 | Hmgcs2 | mmu-miR-34c-5p | -0.51 | 2.99E-03 | 7.80E-02 |
| 53602 | Hpcal1 | mmu-miR-34c-5p | 0.50 | 3.91E-03 | 8.58E-02 |
| 14151 | Fech | mmu-miR-34c-5p | 0.52 | 2.17E-03 | 6.67E-02 |
| 57259 | Tob2 | mmu-miR-34c-5p | -0.49 | 4.17E-03 | 8.70E-02 |
| 65254 | Dpysl5 | mmu-miR-34c-5p | -0.48 | 5.52E-03 | 9.70E-02 |
| 76559 | Atg2b | mmu-miR-34c-5p | -0.49 | 4.11E-03 | 8.70E-02 |
| 99890 | Prmt6 | mmu-miR-34c-5p | 0.54 | 1.37E-03 | 5.56E-02 |
| 26987 | Eif4e2 | mmu-miR-34c-5p | 0.51 | 3.16E-03 | 7.86E-02 |
| 56494 | Gosr2 | mmu-miR-34c-5p | 0.48 | 4.93E-03 | 9.32E-02 |
| 100045228 | LOC100045228 | mmu-miR-34c-5p | -0.50 | 3.65E-03 | 8.41E-02 |
| 102247 | Agpat6 | mmu-miR-34c-5p | -0.59 | 3.37E-04 | 3.50E-02 |
| 100046120 | LOC100046120 | mmu-miR-34c-5p | 0.50 | 3.63E-03 | 8.41E-02 |
| 15384 | Hnrnpab | mmu-miR-34c-5p | -0.49 | 4.32E-03 | 8.81E-02 |
| 230757 | 5730409E04Rik | mmu-miR-34c-5p | -0.48 | 5.53E-03 | 9.70E-02 |
| 68024 | Hist1h2bc | mmu-miR-34c-5p | -0.54 | 1.48E-03 | 5.73E-02 |
| 17760 | Mtap6 | mmu-miR-34c-5p | 0.52 | 2.52E-03 | 7.12E-02 |
| 66642 | Ctnnbl1 | mmu-miR-34c-5p | -0.53 | 1.73E-03 | 6.11E-02 |
| 27407 | Abcf2 | mmu-miR-34c-5p | 0.49 | 4.19E-03 | 8.71E-02 |
| 57751 | Rnf25 | mmu-miR-34c-5p | 0.48 | 5.11E-03 | 9.42E-02 |
| 20907 | Stx1a | mmu-miR-34c-5p | 0.49 | 4.02E-03 | 8.63E-02 |
| 73072 | BC068157 | mmu-miR-34c-5p | -0.48 | 5.40E-03 | 9.63E-02 |
| 18173 | Slc11a1 | mmu-miR-34c-5p | -0.54 | 1.40E-03 | 5.62E-02 |
| 103266 | AI597468 | mmu-miR-34c-5p | 0.49 | 4.75E-03 | 9.22E-02 |
| 100041290 | LOC100041290 | mmu-miR-34c-5p | 0.48 | 5.14E-03 | 9.42E-02 |
| 76130 | Las1l | mmu-miR-34c-5p | -0.53 | 1.71E-03 | 6.11E-02 |
| 140499 | Ube2j2 | mmu-miR-34c-5p | 0.50 | 3.77E-03 | 8.42E-02 |
| 110006 | Gusb | mmu-miR-34c-5p | -0.54 | 1.30E-03 | 5.48E-02 |
| 384814 | EG384814 | mmu-miR-34c-5p | -0.48 | 5.83E-03 | 9.93E-02 |
| 19025 | Ctsa | mmu-let-7a-5p | 0.65 | 4.88E-05 | 8.24E-02 |
| 276770 | Eif5a | mmu-let-7a-5p | 0.66 | 4.11E-05 | 8.24E-02 |
| 100046136 | LOC100046136 | mmu-let-7a-5p | 0.65 | 5.95E-05 | 8.24E-02 |
| 236539 | Phgdh | mmu-let-7a-5p | 0.64 | 8.51E-05 | 8.83E-02 |
| 14661 | Lgsn | mmu-let-7a-5p | 0.66 | 3.35E-05 | 8.24E-02 |
| 434858 | EG434858 | mmu-let-7a-5p | 0.65 | 5.71E-05 | 8.24E-02 |
| 11993 | Aup1 | mmu-let-7a-5p | 0.67 | 2.86E-05 | 8.24E-02 |
| 227721 | Ppapdc3 | mmu-let-7a-5p | -0.64 | 7.03E-05 | 8.34E-02 |
| 110891 | Slc8a2 | mmu-miR-320-3p | -0.60 | 2.54E-04 | 6.38E-02 |
| 11490 | Adam15 | mmu-miR-320-3p | -0.57 | 6.68E-04 | 8.09E-02 |
| 381677 | Vgf | mmu-miR-320-3p | -0.54 | 1.55E-03 | 9.60E-02 |
| 27360 | Add3 | mmu-miR-320-3p | 0.54 | 1.52E-03 | 9.60E-02 |
| 242773 | Slc45a1 | mmu-miR-320-3p | -0.53 | 1.64E-03 | 9.84E-02 |
| 235431 | Coro2b | mmu-miR-320-3p | -0.54 | 1.46E-03 | 9.60E-02 |
| 100046003 | LOC100046003 | mmu-miR-320-3p | -0.58 | 4.38E-04 | 6.83E-02 |
| 18626 | Per1 | mmu-miR-320-3p | -0.59 | 4.36E-04 | 6.83E-02 |
| 56395 | Tmem115 | mmu-miR-320-3p | -0.55 | 1.26E-03 | 9.60E-02 |
| 242667 | Dlgap3 | mmu-miR-320-3p | -0.66 | 3.60E-05 | 3.82E-02 |
| 100047659 | LOC100047659 | mmu-miR-320-3p | -0.56 | 8.41E-04 | 8.62E-02 |
| 22340 | Vegfb | mmu-miR-320-3p | -0.54 | 1.35E-03 | 9.60E-02 |
| 21375 | Tbr1 | mmu-miR-320-3p | -0.60 | 2.52E-04 | 6.38E-02 |
| 270109 | Pcnxl2 | mmu-miR-320-3p | -0.54 | 1.53E-03 | 9.60E-02 |
| 57783 | Tnip1 | mmu-miR-320-3p | -0.55 | 1.05E-03 | 9.34E-02 |
| 333331 | LOC333331 | mmu-miR-320-3p | 0.64 | 8.44E-05 | 5.01E-02 |
| 170755 | Sgk3 | mmu-miR-320-3p | -0.59 | 3.86E-04 | 6.67E-02 |
| 16581 | Kifc2 | mmu-miR-320-3p | -0.55 | 1.07E-03 | 9.34E-02 |
| 99010 | Lpcat4 | mmu-miR-320-3p | -0.55 | 1.02E-03 | 9.34E-02 |
| 19415 | Rasal1 | mmu-miR-320-3p | -0.66 | 4.08E-05 | 3.82E-02 |
| 106064 | AW549877 | mmu-miR-320-3p | 0.57 | 6.92E-04 | 8.09E-02 |
| 71310 | Tbc1d9 | mmu-miR-320-3p | -0.57 | 5.90E-04 | 7.78E-02 |
| 19894 | Rph3a | mmu-miR-320-3p | -0.60 | 2.91E-04 | 6.56E-02 |
| 76820 | D12Ertd553e | mmu-miR-320-3p | 0.55 | 1.14E-03 | 9.53E-02 |
| 13864 | Nr2f6 | mmu-miR-320-3p | -0.54 | 1.30E-03 | 9.60E-02 |
| 216869 | Arrb2 | mmu-miR-320-3p | -0.55 | 9.88E-04 | 9.19E-02 |
| 243819 | Saps1 | mmu-miR-320-3p | -0.54 | 1.57E-03 | 9.60E-02 |
| 12545 | Cdc7 | mmu-miR-320-3p | 0.56 | 7.75E-04 | 8.35E-02 |
| 64050 | Yeats4 | mmu-miR-320-3p | -0.59 | 4.08E-04 | 6.83E-02 |
| 20743 | Spnb3 | mmu-miR-320-3p | -0.58 | 4.44E-04 | 6.83E-02 |
| 100046136 | LOC100046136 | mmu-miR-320-3p | 0.59 | 3.57E-04 | 6.56E-02 |
| 68035 | Rbm42 | mmu-miR-320-3p | -0.57 | 7.53E-04 | 8.35E-02 |
| 269643 | Ppp2r2c | mmu-miR-320-3p | -0.61 | 2.01E-04 | 6.38E-02 |
| 100608 | Noc4l | mmu-miR-320-3p | -0.58 | 5.54E-04 | 7.51E-02 |
| 100046959 | LOC100046959 | mmu-miR-320-3p | 0.55 | 1.21E-03 | 9.53E-02 |
| 215303 | Camk1g | mmu-miR-320-3p | -0.59 | 3.66E-04 | 6.56E-02 |
| 229615 | Pias3 | mmu-miR-320-3p | -0.62 | 1.57E-04 | 6.38E-02 |
| 29858 | Pmm1 | mmu-miR-320-3p | -0.54 | 1.41E-03 | 9.60E-02 |
| 13618 | Ednrb | mmu-miR-320-3p | 0.61 | 2.37E-04 | 6.38E-02 |
| 69195 | Tmem121 | mmu-miR-320-3p | -0.65 | 6.03E-05 | 4.30E-02 |
| 545554 | Ankrd34a | mmu-miR-320-3p | -0.63 | 1.21E-04 | 6.23E-02 |
| 16570 | Kif3c | mmu-miR-320-3p | -0.53 | 1.88E-03 | 9.97E-02 |
| 107767 | Scamp1 | mmu-miR-320-3p | 0.56 | 9.39E-04 | 8.97E-02 |
| 20355 | Sema4f | mmu-miR-320-3p | -0.64 | 7.76E-05 | 4.95E-02 |
| 57357 | Srd5a3 | mmu-miR-320-3p | -0.54 | 1.57E-03 | 9.60E-02 |
| 66830 | Nacc1 | mmu-miR-320-3p | -0.55 | 1.18E-03 | 9.53E-02 |
| 320634 | Ocrl | mmu-miR-320-3p | 0.54 | 1.53E-03 | 9.60E-02 |
| 230996 | 9430015G10Rik | mmu-miR-320-3p | -0.56 | 7.91E-04 | 8.35E-02 |
| 211548 | Nomo1 | mmu-miR-320-3p | -0.54 | 1.51E-03 | 9.60E-02 |
| 26895 | Cops7b | mmu-miR-320-3p | -0.53 | 1.82E-03 | 9.97E-02 |
| 214290 | Zcchc6 | mmu-miR-320-3p | 0.54 | 1.56E-03 | 9.60E-02 |
| 269608 | Plekhg5 | mmu-miR-320-3p | -0.62 | 1.60E-04 | 6.38E-02 |
| 15239 | Hgs | mmu-miR-320-3p | -0.55 | 1.20E-03 | 9.53E-02 |
| 54198 | Snx3 | mmu-miR-320-3p | -0.54 | 1.37E-03 | 9.60E-02 |
| 11305 | Abca2 | mmu-miR-320-3p | -0.59 | 3.61E-04 | 6.56E-02 |
| 215653 | Rassf2 | mmu-miR-320-3p | -0.53 | 1.89E-03 | 9.97E-02 |
| 76454 | Fbxo31 | mmu-miR-320-3p | -0.53 | 1.75E-03 | 9.84E-02 |
| 66414 | Ndufa12 | mmu-miR-320-3p | -0.58 | 4.98E-04 | 7.25E-02 |
| 30877 | Gnl3 | mmu-miR-320-3p | 0.59 | 3.29E-04 | 6.56E-02 |
| 12608 | Cebpb | mmu-miR-320-3p | -0.60 | 3.24E-04 | 6.56E-02 |
| 20364 | Sepw1 | mmu-miR-320-3p | -0.56 | 8.00E-04 | 8.35E-02 |
| 228777 | Nrsn2 | mmu-miR-320-3p | 0.57 | 6.85E-04 | 8.09E-02 |
| 50849 | Rnf10 | mmu-miR-320-3p | -0.53 | 1.88E-03 | 9.97E-02 |
| 100044257 | LOC100044257 | mmu-miR-320-3p | -0.54 | 1.32E-03 | 9.60E-02 |
| 230075 | Ndufb6 | mmu-miR-320-3p | -0.71 | 5.46E-06 | 1.13E-02 |
| 83436 | Plekha2 | mmu-miR-320-3p | 0.58 | 4.84E-04 | 7.17E-02 |
| 233900 | Rnf40 | mmu-miR-320-3p | -0.59 | 3.71E-04 | 6.56E-02 |
| 110816 | Pwp2 | mmu-miR-320-3p | -0.55 | 1.24E-03 | 9.60E-02 |
| 12891 | Cpne6 | mmu-miR-320-3p | 0.61 | 1.87E-04 | 6.38E-02 |
| 21429 | Ubtf | mmu-miR-320-3p | -0.59 | 3.55E-04 | 6.56E-02 |
| 58802 | Kcnmb4 | mmu-miR-320-3p | -0.61 | 2.06E-04 | 6.38E-02 |
| 20256 | Clec11a | mmu-miR-320-3p | -0.57 | 7.43E-04 | 8.35E-02 |
| 56433 | Vps29 | mmu-miR-320-3p | -0.65 | 6.22E-05 | 4.30E-02 |
| 56349 | Net1 | mmu-miR-320-3p | -0.55 | 1.22E-03 | 9.53E-02 |
| 434858 | EG434858 | mmu-miR-320-3p | 0.54 | 1.57E-03 | 9.60E-02 |
| 279766 | Rhbdd3 | mmu-miR-320-3p | -0.55 | 1.06E-03 | 9.34E-02 |
| 52815 | Ldhd | mmu-miR-320-3p | -0.54 | 1.52E-03 | 9.60E-02 |
| 20014 | Rpn2 | mmu-miR-320-3p | 0.61 | 2.11E-04 | 6.38E-02 |
| 14979 | H2-Ke6 | mmu-miR-320-3p | 0.53 | 1.83E-03 | 9.97E-02 |
| 14415 | Gad1 | mmu-miR-320-3p | 0.55 | 1.21E-03 | 9.53E-02 |
| 66431 | 1810049H13Rik | mmu-miR-320-3p | -0.56 | 7.68E-04 | 8.35E-02 |
| 194237 | BC057371 | mmu-miR-320-3p | 0.55 | 1.16E-03 | 9.53E-02 |
| 72265 | Tram1 | mmu-miR-320-3p | 0.54 | 1.39E-03 | 9.60E-02 |
| 94280 | Sfxn3 | mmu-miR-320-3p | -0.56 | 9.50E-04 | 8.97E-02 |
| 100044576 | LOC100044576 | mmu-miR-320-3p | -0.62 | 1.32E-04 | 6.23E-02 |
| 230857 | Ece1 | mmu-miR-320-3p | -0.58 | 5.61E-04 | 7.51E-02 |
| 16594 | Klc2 | mmu-miR-320-3p | -0.60 | 2.46E-04 | 6.38E-02 |
| 76974 | 1190003J15Rik | mmu-miR-320-3p | -0.62 | 1.35E-04 | 6.23E-02 |
| 67095 | Trak1 | mmu-miR-320-3p | -0.53 | 1.69E-03 | 9.84E-02 |
| 52064 | Coq5 | mmu-miR-320-3p | 0.54 | 1.41E-03 | 9.60E-02 |
| 102693 | Phldb1 | mmu-miR-320-3p | 0.58 | 5.43E-04 | 7.51E-02 |
| 67800 | Dgat2 | mmu-miR-320-3p | -0.56 | 9.38E-04 | 8.97E-02 |
| 245684 | Cnksr2 | mmu-miR-320-3p | 0.53 | 1.88E-03 | 9.97E-02 |
| 17183 | Matn4 | mmu-miR-320-3p | 0.53 | 1.74E-03 | 9.84E-02 |
| 11546 | Parp2 | mmu-miR-320-3p | 0.57 | 6.05E-04 | 7.85E-02 |
| 58887 | Repin1 | mmu-miR-320-3p | -0.53 | 1.83E-03 | 9.97E-02 |
| 107448 | Unc5a | mmu-miR-320-3p | -0.54 | 1.39E-03 | 9.60E-02 |
| 233208 | Scaf1 | mmu-miR-320-3p | -0.53 | 1.70E-03 | 9.84E-02 |
| 240025 | Dact2 | mmu-miR-320-3p | -0.60 | 2.59E-04 | 6.38E-02 |
| 217125 | Samd14 | mmu-miR-320-3p | 0.53 | 1.75E-03 | 9.84E-02 |
| 208718 | Dis3l2 | mmu-miR-320-3p | -0.54 | 1.31E-03 | 9.60E-02 |
| 16834 | Cog1 | mmu-miR-320-3p | -0.56 | 7.71E-04 | 8.35E-02 |
| 22218 | Sumo1 | mmu-miR-320-3p | -0.55 | 1.15E-03 | 9.53E-02 |
| 212276 | Zfp748 | mmu-miR-320-3p | 0.53 | 1.72E-03 | 9.84E-02 |
| 22225 | Usp5 | mmu-miR-320-3p | -0.75 | 7.11E-07 | 5.38E-03 |
| 320299 | Iqcb1 | mmu-miR-320-3p | 0.58 | 4.60E-04 | 6.95E-02 |
| 59035 | Carm1 | mmu-miR-320-3p | -0.61 | 2.23E-04 | 6.38E-02 |
| 268567 | 6330442E10Rik | mmu-miR-320-3p | -0.57 | 6.66E-04 | 8.09E-02 |
| 15574 | Hus1 | mmu-miR-320-3p | 0.60 | 3.00E-04 | 6.56E-02 |
| 12424 | Cck | mmu-miR-320-3p | -0.55 | 1.08E-03 | 9.34E-02 |
| 108100 | Baiap2 | mmu-miR-320-3p | 0.59 | 3.48E-04 | 6.56E-02 |
| 216345 | Zfc3h1 | mmu-miR-320-3p | 0.54 | 1.56E-03 | 9.60E-02 |
| 234776 | Atmin | mmu-miR-320-3p | -0.63 | 1.29E-04 | 6.23E-02 |
| 18563 | Pcx | mmu-miR-320-3p | -0.59 | 4.28E-04 | 6.83E-02 |
| 56463 | Snd1 | mmu-miR-320-3p | -0.55 | 1.17E-03 | 9.53E-02 |
| 140919 | Slc17a6 | mmu-miR-320-3p | 0.62 | 1.73E-04 | 6.38E-02 |
| 22187 | Ubb | mmu-miR-320-3p | 0.53 | 1.84E-03 | 9.97E-02 |
| 65956 | Ccl21c | mmu-miR-320-3p | -0.55 | 1.22E-03 | 9.53E-02 |
| 77630 | A730035I17Rik | mmu-miR-320-3p | -0.55 | 1.06E-03 | 9.34E-02 |
| 105859 | Csdc2 | mmu-miR-320-3p | 0.65 | 5.20E-05 | 4.30E-02 |
| 20399 | Sh2b1 | mmu-miR-320-3p | -0.54 | 1.36E-03 | 9.60E-02 |
| 66416 | Ndufa7 | mmu-miR-320-3p | -0.56 | 9.12E-04 | 8.97E-02 |
| 73296 | Rhobtb3 | mmu-miR-320-3p | 0.55 | 9.96E-04 | 9.19E-02 |
| 14160 | Lgr5 | mmu-miR-320-3p | 0.59 | 4.29E-04 | 6.83E-02 |
| 68801 | Elovl5 | mmu-miR-320-3p | 0.61 | 2.00E-04 | 6.38E-02 |
| 226154 | Lzts2 | mmu-miR-320-3p | 0.53 | 1.77E-03 | 9.89E-02 |
| 20452 | St8sia4 | mmu-miR-320-3p | 0.53 | 1.73E-03 | 9.84E-02 |
| 12308 | Calb2 | mmu-miR-320-3p | 0.54 | 1.45E-03 | 9.60E-02 |
| 232441 | Rerg | mmu-miR-320-3p | 0.66 | 4.14E-05 | 3.82E-02 |
| 100764 | 1110008J03Rik | mmu-miR-320-3p | -0.53 | 1.62E-03 | 9.84E-02 |
| 70762 | Dclk2 | mmu-miR-320-3p | 0.57 | 6.48E-04 | 8.09E-02 |
| 76510 | Trappc9 | mmu-miR-320-3p | -0.54 | 1.43E-03 | 9.60E-02 |
| 225631 | Onecut2 | mmu-miR-320-3p | -0.57 | 6.67E-04 | 8.09E-02 |
| 50771 | Atp9b | mmu-miR-320-3p | -0.72 | 3.76E-06 | 1.04E-02 |
| 72658 | 2700097O09Rik | mmu-miR-320-3p | 0.56 | 9.01E-04 | 8.97E-02 |
| 54723 | Tfip11 | mmu-miR-320-3p | -0.66 | 3.72E-05 | 3.82E-02 |
| 433224 | EG433224 | mmu-miR-320-3p | 0.53 | 1.73E-03 | 9.84E-02 |
| 20833 | Ssrp1 | mmu-miR-320-3p | -0.57 | 6.89E-04 | 8.09E-02 |
| 217232 | Cdc27 | mmu-miR-320-3p | -0.61 | 2.02E-04 | 6.38E-02 |
| 100047353 | LOC100047353 | mmu-miR-320-3p | 0.68 | 1.80E-05 | 2.99E-02 |
| 72102 | Dusp11 | mmu-miR-320-3p | 0.53 | 1.70E-03 | 9.84E-02 |
| 67414 | Mfn1 | mmu-miR-320-3p | 0.60 | 2.75E-04 | 6.53E-02 |
| 12226 | Btg1 | mmu-miR-320-3p | 0.60 | 2.61E-04 | 6.38E-02 |
| 66413 | Psmd6 | mmu-miR-320-3p | -0.54 | 1.29E-03 | 9.60E-02 |
| 57138 | Slc12a5 | mmu-miR-320-3p | -0.56 | 9.21E-04 | 8.97E-02 |
| 319180 | Hist1h2bf | mmu-miR-320-3p | 0.56 | 7.62E-04 | 8.35E-02 |
| 208076 | Pknox2 | mmu-miR-320-3p | -0.56 | 9.42E-04 | 8.97E-02 |
| 19286 | Pts | mmu-miR-320-3p | 0.59 | 3.70E-04 | 6.56E-02 |
| 26373 | Clcn7 | mmu-miR-320-3p | -0.53 | 1.70E-03 | 9.84E-02 |
| 100045359 | LOC100045359 | mmu-miR-320-3p | -0.54 | 1.38E-03 | 9.60E-02 |
| 22044 | Trh | mmu-miR-320-3p | 0.54 | 1.38E-03 | 9.60E-02 |
| 13446 | Doc2a | mmu-miR-320-3p | -0.74 | 1.30E-06 | 5.38E-03 |
| 112403 | Dom3z | mmu-miR-320-3p | 0.54 | 1.44E-03 | 9.60E-02 |
| 53972 | Ngef | mmu-miR-320-3p | -0.58 | 5.37E-04 | 7.51E-02 |
| 12988 | Csk | mmu-miR-320-3p | 0.53 | 1.90E-03 | 9.97E-02 |
| 242736 | 4732496O08Rik | mmu-miR-320-3p | 0.59 | 3.71E-04 | 6.56E-02 |
| 13730 | Emp1 | mmu-miR-320-3p | 0.58 | 5.50E-04 | 7.51E-02 |
| 18000 | 40057 | mmu-miR-320-3p | 0.56 | 8.04E-04 | 8.35E-02 |
| 242773 | Slc45a1 | mmu-let-7i-5p | -0.63 | 1.16E-04 | 9.97E-02 |
| 19025 | Ctsa | mmu-let-7i-5p | 0.67 | 2.35E-05 | 4.89E-02 |
| 53379 | Hnrnpa2b1 | mmu-let-7i-5p | 0.63 | 1.20E-04 | 9.97E-02 |
| 276770 | Eif5a | mmu-let-7i-5p | 0.65 | 5.94E-05 | 9.86E-02 |
| 100046136 | LOC100046136 | mmu-let-7i-5p | 0.70 | 6.69E-06 | 2.48E-02 |
| 545554 | Ankrd34a | mmu-let-7i-5p | -0.63 | 1.15E-04 | 9.97E-02 |
| 100046744 | LOC100046744 | mmu-let-7i-5p | -0.64 | 8.44E-05 | 9.97E-02 |
| 14661 | Lgsn | mmu-let-7i-5p | 0.70 | 8.95E-06 | 2.48E-02 |
| 434858 | EG434858 | mmu-let-7i-5p | 0.64 | 8.69E-05 | 9.97E-02 |
| 11993 | Aup1 | mmu-let-7i-5p | 0.72 | 3.15E-06 | 2.48E-02 |
| 236539 | Phgdh | mmu-miR-101b-3p | 0.67 | 3.05E-05 | 8.44E-02 |
| 20910 | Stxbp1 | mmu-miR-101b-3p | 0.64 | 7.11E-05 | 9.84E-02 |
| 14168 | Fgf13 | mmu-miR-101b-3p | 0.65 | 5.38E-05 | 9.84E-02 |
| 11769 | Ap1s1 | mmu-miR-101b-3p | 0.69 | 1.53E-05 | 7.04E-02 |
| 12733 | Clcnka | mmu-miR-101b-3p | -0.64 | 6.92E-05 | 9.84E-02 |
| 71989 | Rpusd4 | mmu-miR-101b-3p | -0.68 | 1.70E-05 | 7.04E-02 |
| 110891 | Slc8a2 | mmu-miR-24-2-5p | -0.62 | 1.41E-04 | 4.33E-02 |
| 57740 | Stk32c | mmu-miR-24-2-5p | -0.61 | 2.22E-04 | 5.30E-02 |
| 266781 | Snx17 | mmu-miR-24-2-5p | 0.62 | 1.77E-04 | 4.91E-02 |
| 218194 | Phactr1 | mmu-miR-24-2-5p | 0.59 | 3.55E-04 | 6.64E-02 |
| 17748 | Mt1 | mmu-miR-24-2-5p | -0.56 | 8.79E-04 | 9.35E-02 |
| 242773 | Slc45a1 | mmu-miR-24-2-5p | -0.61 | 2.40E-04 | 5.34E-02 |
| 229521 | Syt11 | mmu-miR-24-2-5p | 0.56 | 9.70E-04 | 9.71E-02 |
| 69080 | Gmppa | mmu-miR-24-2-5p | 0.57 | 5.87E-04 | 8.70E-02 |
| 57776 | Ttyh1 | mmu-miR-24-2-5p | 0.61 | 1.97E-04 | 5.22E-02 |
| 72503 | 2610507B11Rik | mmu-miR-24-2-5p | 0.62 | 1.40E-04 | 4.33E-02 |
| 19025 | Ctsa | mmu-miR-24-2-5p | 0.67 | 2.30E-05 | 3.02E-02 |
| 21807 | Tsc22d1 | mmu-miR-24-2-5p | 0.61 | 2.23E-04 | 5.30E-02 |
| 53379 | Hnrnpa2b1 | mmu-miR-24-2-5p | 0.63 | 1.13E-04 | 4.33E-02 |
| 71310 | Tbc1d9 | mmu-miR-24-2-5p | -0.56 | 8.48E-04 | 9.14E-02 |
| 59003 | Maea | mmu-miR-24-2-5p | 0.58 | 5.59E-04 | 8.68E-02 |
| 224997 | Dlgap1 | mmu-miR-24-2-5p | 0.62 | 1.37E-04 | 4.33E-02 |
| 276770 | Eif5a | mmu-miR-24-2-5p | 0.63 | 9.63E-05 | 4.33E-02 |
| 67087 | Ctnnbip1 | mmu-miR-24-2-5p | -0.63 | 1.12E-04 | 4.33E-02 |
| 20692 | Sparc | mmu-miR-24-2-5p | -0.56 | 8.26E-04 | 9.14E-02 |
| 18642 | Pfkm | mmu-miR-24-2-5p | 0.65 | 5.23E-05 | 3.95E-02 |
| 100046136 | LOC100046136 | mmu-miR-24-2-5p | 0.61 | 2.01E-04 | 5.22E-02 |
| 236539 | Phgdh | mmu-miR-24-2-5p | 0.59 | 4.15E-04 | 7.34E-02 |
| 12153 | Bmp1 | mmu-miR-24-2-5p | 0.56 | 9.48E-04 | 9.71E-02 |
| 100608 | Noc4l | mmu-miR-24-2-5p | -0.57 | 7.44E-04 | 8.85E-02 |
| 70231 | Gorasp2 | mmu-miR-24-2-5p | 0.65 | 6.59E-05 | 4.33E-02 |
| 11545 | Parp1 | mmu-miR-24-2-5p | -0.64 | 7.81E-05 | 4.33E-02 |
| 66513 | Map3k7ip1 | mmu-miR-24-2-5p | -0.58 | 4.48E-04 | 7.59E-02 |
| 56149 | Grasp | mmu-miR-24-2-5p | 0.56 | 8.45E-04 | 9.14E-02 |
| 13628 | Eef1a2 | mmu-miR-24-2-5p | 0.67 | 2.54E-05 | 3.02E-02 |
| 100046744 | LOC100046744 | mmu-miR-24-2-5p | -0.71 | 5.58E-06 | 1.56E-02 |
| 228355 | Madd | mmu-miR-24-2-5p | 0.66 | 4.24E-05 | 3.52E-02 |
| 69780 | Smap2 | mmu-miR-24-2-5p | -0.56 | 7.95E-04 | 8.92E-02 |
| 20910 | Stxbp1 | mmu-miR-24-2-5p | 0.71 | 5.65E-06 | 1.56E-02 |
| 16906 | Lmnb1 | mmu-miR-24-2-5p | -0.63 | 1.19E-04 | 4.33E-02 |
| 67420 | Far1 | mmu-miR-24-2-5p | 0.59 | 3.29E-04 | 6.50E-02 |
| 66366 | Ergic3 | mmu-miR-24-2-5p | 0.57 | 7.46E-04 | 8.85E-02 |
| 14661 | Lgsn | mmu-miR-24-2-5p | 0.73 | 2.07E-06 | 1.56E-02 |
| 228998 | Arfgap1 | mmu-miR-24-2-5p | 0.57 | 6.77E-04 | 8.85E-02 |
| 240753 | Plekha6 | mmu-miR-24-2-5p | 0.59 | 4.12E-04 | 7.34E-02 |
| 108124 | Napa | mmu-miR-24-2-5p | 0.56 | 8.90E-04 | 9.35E-02 |
| 70025 | Acot7 | mmu-miR-24-2-5p | 0.70 | 8.93E-06 | 1.85E-02 |
| 68585 | Rtn4 | mmu-miR-24-2-5p | 0.57 | 7.23E-04 | 8.85E-02 |
| 140580 | Elmo1 | mmu-miR-24-2-5p | 0.57 | 7.14E-04 | 8.85E-02 |
| 233900 | Rnf40 | mmu-miR-24-2-5p | -0.64 | 8.74E-05 | 4.33E-02 |
| 30853 | Mlf2 | mmu-miR-24-2-5p | 0.58 | 4.83E-04 | 8.02E-02 |
| 15510 | Hspd1 | mmu-miR-24-2-5p | 0.57 | 7.25E-04 | 8.85E-02 |
| 21429 | Ubtf | mmu-miR-24-2-5p | -0.56 | 9.47E-04 | 9.71E-02 |
| 56421 | Pfkp | mmu-miR-24-2-5p | 0.58 | 5.02E-04 | 8.18E-02 |
| 54376 | Cacng3 | mmu-miR-24-2-5p | 0.57 | 6.56E-04 | 8.85E-02 |
| 212627 | Prpsap2 | mmu-miR-24-2-5p | 0.64 | 8.71E-05 | 4.33E-02 |
| 57437 | Golga7 | mmu-miR-24-2-5p | 0.69 | 1.18E-05 | 1.97E-02 |
| 103711 | Pnpo | mmu-miR-24-2-5p | 0.57 | 7.28E-04 | 8.85E-02 |
| 329777 | Pigk | mmu-miR-24-2-5p | 0.58 | 5.64E-04 | 8.68E-02 |
| 27096 | Trappc3 | mmu-miR-24-2-5p | 0.56 | 7.59E-04 | 8.85E-02 |
| 65257 | Asb3 | mmu-miR-24-2-5p | 0.56 | 9.60E-04 | 9.71E-02 |
| 102657 | Cd276 | mmu-miR-24-2-5p | -0.64 | 9.26E-05 | 4.33E-02 |
| 56807 | Scamp5 | mmu-miR-24-2-5p | 0.57 | 7.30E-04 | 8.85E-02 |
| 11993 | Aup1 | mmu-miR-24-2-5p | 0.66 | 3.95E-05 | 3.52E-02 |
| 11769 | Ap1s1 | mmu-miR-24-2-5p | 0.62 | 1.53E-04 | 4.52E-02 |
| 100044862 | LOC100044862 | mmu-miR-24-2-5p | 0.61 | 2.35E-04 | 5.34E-02 |
| 231148 | Ablim2 | mmu-miR-24-2-5p | 0.67 | 2.91E-05 | 3.02E-02 |
| 26950 | Vsnl1 | mmu-miR-24-2-5p | 0.58 | 5.77E-04 | 8.70E-02 |
| 20586 | Smarca4 | mmu-miR-24-2-5p | -0.60 | 2.45E-04 | 5.34E-02 |
| 72895 | Setd5 | mmu-miR-24-2-5p | -0.57 | 6.13E-04 | 8.85E-02 |
| 27643 | Ubl4 | mmu-miR-24-2-5p | 0.63 | 1.28E-04 | 4.33E-02 |
| 97820 | 4833439L19Rik | mmu-miR-24-2-5p | -0.59 | 3.60E-04 | 6.64E-02 |
| 24000 | Ptpn21 | mmu-miR-24-2-5p | -0.63 | 1.20E-04 | 4.33E-02 |
| 227721 | Ppapdc3 | mmu-miR-24-2-5p | -0.59 | 4.34E-04 | 7.51E-02 |
| 26987 | Eif4e2 | mmu-miR-24-2-5p | 0.56 | 7.64E-04 | 8.85E-02 |
| 16210 | Impact | mmu-miR-24-2-5p | 0.60 | 2.95E-04 | 6.24E-02 |
| 102247 | Agpat6 | mmu-miR-24-2-5p | -0.62 | 1.74E-04 | 4.91E-02 |
| 230757 | 5730409E04Rik | mmu-miR-24-2-5p | -0.63 | 1.03E-04 | 4.33E-02 |
| 17760 | Mtap6 | mmu-miR-24-2-5p | 0.64 | 6.82E-05 | 4.33E-02 |
| 66642 | Ctnnbl1 | mmu-miR-24-2-5p | -0.60 | 3.22E-04 | 6.50E-02 |
| 27407 | Abcf2 | mmu-miR-24-2-5p | 0.60 | 3.01E-04 | 6.24E-02 |
| 20907 | Stx1a | mmu-miR-24-2-5p | 0.57 | 6.46E-04 | 8.85E-02 |
| 76130 | Las1l | mmu-miR-24-2-5p | -0.57 | 6.65E-04 | 8.85E-02 |
| 67896 | Ccdc80 | mmu-miR-24-2-5p | -0.61 | 2.17E-04 | 5.30E-02 |
| 76789 | 2410129H14Rik | mmu-miR-24-2-5p | 0.56 | 7.68E-04 | 8.85E-02 |
| 72514 | Fgfbp3 | mmu-miR-24-2-5p | -0.56 | 7.94E-04 | 8.92E-02 |
| 13116 | Cyp46a1 | mmu-miR-24-2-5p | 0.59 | 3.37E-04 | 6.50E-02 |
| 224630 | Bnip1 | mmu-miR-24-2-5p | 0.58 | 5.33E-04 | 8.52E-02 |
| 235574 | Atp2c1 | mmu-miR-24-2-5p | 0.57 | 6.92E-04 | 8.85E-02 |
| 57740 | Stk32c | mmu-miR-29a-5p | -0.68 | 1.54E-05 | 3.06E-02 |
| 266781 | Snx17 | mmu-miR-29a-5p | 0.60 | 3.20E-04 | 6.82E-02 |
| 218194 | Phactr1 | mmu-miR-29a-5p | 0.56 | 8.23E-04 | 9.81E-02 |
| 11461 | Actb | mmu-miR-29a-5p | 0.63 | 1.05E-04 | 4.25E-02 |
| 17748 | Mt1 | mmu-miR-29a-5p | -0.58 | 4.41E-04 | 7.40E-02 |
| 78928 | Pigt | mmu-miR-29a-5p | -0.60 | 2.77E-04 | 6.28E-02 |
| 242773 | Slc45a1 | mmu-miR-29a-5p | -0.65 | 6.25E-05 | 3.80E-02 |
| 78465 | 1700084C01Rik | mmu-miR-29a-5p | -0.57 | 7.39E-04 | 9.60E-02 |
| 75751 | Ipo4 | mmu-miR-29a-5p | -0.59 | 3.77E-04 | 7.11E-02 |
| 72503 | 2610507B11Rik | mmu-miR-29a-5p | 0.57 | 6.63E-04 | 8.88E-02 |
| 19025 | Ctsa | mmu-miR-29a-5p | 0.63 | 1.09E-04 | 4.25E-02 |
| 21807 | Tsc22d1 | mmu-miR-29a-5p | 0.64 | 9.33E-05 | 4.25E-02 |
| 53379 | Hnrnpa2b1 | mmu-miR-29a-5p | 0.65 | 6.70E-05 | 3.80E-02 |
| 432763 | Prr7 | mmu-miR-29a-5p | -0.66 | 3.65E-05 | 3.72E-02 |
| 224997 | Dlgap1 | mmu-miR-29a-5p | 0.63 | 1.00E-04 | 4.25E-02 |
| 276770 | Eif5a | mmu-miR-29a-5p | 0.65 | 6.12E-05 | 3.80E-02 |
| 76820 | D12Ertd553e | mmu-miR-29a-5p | 0.56 | 8.27E-04 | 9.81E-02 |
| 18642 | Pfkm | mmu-miR-29a-5p | 0.58 | 4.49E-04 | 7.40E-02 |
| 11518 | Add1 | mmu-miR-29a-5p | 0.58 | 5.36E-04 | 7.87E-02 |
| 319477 | 6030419C18Rik | mmu-miR-29a-5p | -0.65 | 6.10E-05 | 3.80E-02 |
| 100046136 | LOC100046136 | mmu-miR-29a-5p | 0.62 | 1.77E-04 | 5.24E-02 |
| 236539 | Phgdh | mmu-miR-29a-5p | 0.68 | 2.24E-05 | 3.10E-02 |
| 100608 | Noc4l | mmu-miR-29a-5p | -0.56 | 7.54E-04 | 9.63E-02 |
| 70231 | Gorasp2 | mmu-miR-29a-5p | 0.58 | 4.85E-04 | 7.60E-02 |
| 66513 | Map3k7ip1 | mmu-miR-29a-5p | -0.66 | 4.04E-05 | 3.72E-02 |
| 56149 | Grasp | mmu-miR-29a-5p | 0.57 | 6.59E-04 | 8.88E-02 |
| 71777 | Ing3 | mmu-miR-29a-5p | 0.58 | 4.64E-04 | 7.40E-02 |
| 13628 | Eef1a2 | mmu-miR-29a-5p | 0.61 | 2.27E-04 | 5.40E-02 |
| 71947 | 2310067B10Rik | mmu-miR-29a-5p | -0.56 | 7.94E-04 | 9.81E-02 |
| 100046744 | LOC100046744 | mmu-miR-29a-5p | -0.73 | 2.01E-06 | 1.67E-02 |
| 71916 | Dus4l | mmu-miR-29a-5p | -0.56 | 8.10E-04 | 9.81E-02 |
| 228355 | Madd | mmu-miR-29a-5p | 0.61 | 2.07E-04 | 5.36E-02 |
| 20910 | Stxbp1 | mmu-miR-29a-5p | 0.70 | 8.92E-06 | 3.06E-02 |
| 16906 | Lmnb1 | mmu-miR-29a-5p | -0.59 | 4.03E-04 | 7.40E-02 |
| 66366 | Ergic3 | mmu-miR-29a-5p | 0.57 | 7.40E-04 | 9.60E-02 |
| 14661 | Lgsn | mmu-miR-29a-5p | 0.64 | 7.74E-05 | 4.02E-02 |
| 219022 | Ttc5 | mmu-miR-29a-5p | 0.60 | 2.80E-04 | 6.28E-02 |
| 240753 | Plekha6 | mmu-miR-29a-5p | 0.59 | 3.76E-04 | 7.11E-02 |
| 70025 | Acot7 | mmu-miR-29a-5p | 0.65 | 5.86E-05 | 3.80E-02 |
| 233900 | Rnf40 | mmu-miR-29a-5p | -0.59 | 3.55E-04 | 7.02E-02 |
| 56421 | Pfkp | mmu-miR-29a-5p | 0.59 | 3.50E-04 | 7.02E-02 |
| 241846 | Lsm14b | mmu-miR-29a-5p | -0.59 | 4.18E-04 | 7.40E-02 |
| 20351 | Sema4a | mmu-miR-29a-5p | 0.58 | 5.68E-04 | 8.13E-02 |
| 57437 | Golga7 | mmu-miR-29a-5p | 0.62 | 1.32E-04 | 4.77E-02 |
| 20810 | Srm | mmu-miR-29a-5p | 0.58 | 5.32E-04 | 7.87E-02 |
| 329777 | Pigk | mmu-miR-29a-5p | 0.57 | 5.91E-04 | 8.32E-02 |
| 72029 | Cnpy3 | mmu-miR-29a-5p | 0.61 | 2.02E-04 | 5.36E-02 |
| 12048 | Bcl2l1 | mmu-miR-29a-5p | 0.61 | 1.93E-04 | 5.35E-02 |
| 65257 | Asb3 | mmu-miR-29a-5p | 0.59 | 4.21E-04 | 7.40E-02 |
| 102657 | Cd276 | mmu-miR-29a-5p | -0.68 | 1.79E-05 | 3.06E-02 |
| 30934 | Tor1b | mmu-miR-29a-5p | -0.56 | 8.42E-04 | 9.85E-02 |
| 11993 | Aup1 | mmu-miR-29a-5p | 0.66 | 3.51E-05 | 3.72E-02 |
| 11769 | Ap1s1 | mmu-miR-29a-5p | 0.64 | 6.87E-05 | 3.80E-02 |
| 231148 | Ablim2 | mmu-miR-29a-5p | 0.63 | 1.13E-04 | 4.25E-02 |
| 72895 | Setd5 | mmu-miR-29a-5p | -0.62 | 1.65E-04 | 5.24E-02 |
| 27643 | Ubl4 | mmu-miR-29a-5p | 0.61 | 2.26E-04 | 5.40E-02 |
| 236792 | Tmem32 | mmu-miR-29a-5p | -0.62 | 1.49E-04 | 4.93E-02 |
| 24000 | Ptpn21 | mmu-miR-29a-5p | -0.57 | 6.20E-04 | 8.58E-02 |
| 12460 | Ccs | mmu-miR-29a-5p | -0.61 | 1.93E-04 | 5.35E-02 |
| 53602 | Hpcal1 | mmu-miR-29a-5p | 0.61 | 2.27E-04 | 5.40E-02 |
| 26987 | Eif4e2 | mmu-miR-29a-5p | 0.62 | 1.72E-04 | 5.24E-02 |
| 17760 | Mtap6 | mmu-miR-29a-5p | 0.59 | 3.52E-04 | 7.02E-02 |
| 240332 | Slc6a7 | mmu-miR-29a-5p | 0.62 | 1.45E-04 | 4.93E-02 |
| 12733 | Clcnka | mmu-miR-29a-5p | -0.58 | 5.41E-04 | 7.87E-02 |
| 66642 | Ctnnbl1 | mmu-miR-29a-5p | -0.59 | 4.32E-04 | 7.40E-02 |
| 27407 | Abcf2 | mmu-miR-29a-5p | 0.58 | 5.23E-04 | 7.87E-02 |
| 60321 | Wbp11 | mmu-miR-29a-5p | -0.60 | 3.08E-04 | 6.73E-02 |
| 67896 | Ccdc80 | mmu-miR-29a-5p | -0.56 | 7.75E-04 | 9.74E-02 |
| 100048299 | LOC100048299 | mmu-miR-29a-5p | 0.58 | 4.56E-04 | 7.40E-02 |
| 30838 | Fbxw4 | mmu-miR-29a-5p | 0.63 | 9.97E-05 | 4.25E-02 |
| 13116 | Cyp46a1 | mmu-miR-29a-5p | 0.68 | 1.84E-05 | 3.06E-02 |
| 20910 | Stxbp1 | mmu-miR-376b-3p | 0.69 | 1.27E-05 | 3.50E-02 |
| 14661 | Lgsn | mmu-miR-376b-3p | 0.68 | 2.03E-05 | 4.22E-02 |
| 11993 | Aup1 | mmu-miR-376b-3p | 0.65 | 4.76E-05 | 7.90E-02 |
| 11769 | Ap1s1 | mmu-miR-376b-3p | 0.69 | 1.21E-05 | 3.50E-02 |
| 17760 | Mtap6 | mmu-miR-376b-3p | 0.73 | 1.79E-06 | 1.48E-02 |
| 20910 | Stxbp1 | mmu-miR-181d-5p | 0.66 | 3.47E-05 | 9.59E-02 |
| 102657 | Cd276 | mmu-miR-181d-5p | -0.67 | 3.18E-05 | 9.59E-02 |
| 11769 | Ap1s1 | mmu-miR-181d-5p | 0.71 | 4.47E-06 | 3.71E-02 |
| 20910 | Stxbp1 | mmu-miR-434-3p | 0.72 | 4.04E-06 | 3.36E-02 |
| 276770 | Eif5a | mmu-miR-24-3p | 0.65 | 6.15E-05 | 8.24E-02 |
| 20910 | Stxbp1 | mmu-miR-24-3p | 0.74 | 1.21E-06 | 8.91E-03 |
| 14661 | Lgsn | mmu-miR-24-3p | 0.68 | 1.68E-05 | 3.49E-02 |
| 11769 | Ap1s1 | mmu-miR-24-3p | 0.72 | 3.22E-06 | 8.91E-03 |
| 231148 | Ablim2 | mmu-miR-24-3p | 0.65 | 5.00E-05 | 8.24E-02 |
| 56085 | Ubqln1 | mmu-miR-24-3p | 0.64 | 9.12E-05 | 9.47E-02 |
| 17760 | Mtap6 | mmu-miR-24-3p | 0.73 | 2.45E-06 | 8.91E-03 |
| 27407 | Abcf2 | mmu-miR-24-3p | 0.64 | 6.95E-05 | 8.24E-02 |
| 20910 | Stxbp1 | mmu-miR-222-3p | 0.68 | 1.61E-05 | 4.45E-02 |
| 102657 | Cd276 | mmu-miR-222-3p | -0.69 | 1.44E-05 | 4.45E-02 |
| 11769 | Ap1s1 | mmu-miR-222-3p | 0.69 | 1.35E-05 | 4.45E-02 |
| 236539 | Phgdh | mmu-miR-193-3p | 0.68 | 1.72E-05 | 6.55E-02 |
| 20910 | Stxbp1 | mmu-miR-193-3p | 0.70 | 8.42E-06 | 6.55E-02 |
| 11769 | Ap1s1 | mmu-miR-193-3p | 0.67 | 2.37E-05 | 6.55E-02 |
| 20910 | Stxbp1 | mmu-miR-335-5p | 0.75 | 6.97E-07 | 5.79E-03 |
| 11769 | Ap1s1 | mmu-miR-335-5p | 0.70 | 7.14E-06 | 2.96E-02 |
| 27360 | Add3 | mmu-miR-204-5p | 0.62 | 1.36E-04 | 8.79E-02 |
| 76267 | Fads1 | mmu-miR-204-5p | 0.61 | 2.44E-04 | 8.79E-02 |
| 230101 | Gba2 | mmu-miR-204-5p | -0.61 | 2.15E-04 | 8.79E-02 |
| 13618 | Ednrb | mmu-miR-204-5p | 0.61 | 2.41E-04 | 8.79E-02 |
| 17920 | Myo6 | mmu-miR-204-5p | 0.61 | 2.10E-04 | 8.79E-02 |
| 69195 | Tmem121 | mmu-miR-204-5p | -0.64 | 9.38E-05 | 8.79E-02 |
| 83962 | Btbd1 | mmu-miR-204-5p | 0.65 | 6.19E-05 | 7.84E-02 |
| 21417 | Zeb1 | mmu-miR-204-5p | 0.72 | 3.81E-06 | 3.16E-02 |
| 13193 | Dcx | mmu-miR-204-5p | 0.61 | 2.17E-04 | 8.79E-02 |
| 14199 | Fhl1 | mmu-miR-204-5p | 0.61 | 2.16E-04 | 8.79E-02 |
| 17775 | Laptm4a | mmu-miR-204-5p | -0.61 | 2.33E-04 | 8.79E-02 |
| 58802 | Kcnmb4 | mmu-miR-204-5p | -0.65 | 6.03E-05 | 7.84E-02 |
| 69168 | Bola1 | mmu-miR-204-5p | -0.65 | 6.61E-05 | 7.84E-02 |
| 74246 | Gale | mmu-miR-204-5p | -0.69 | 1.49E-05 | 6.19E-02 |
| 19277 | Ptpro | mmu-miR-204-5p | 0.62 | 1.61E-04 | 8.79E-02 |
| 12632 | Cfl2 | mmu-miR-204-5p | -0.61 | 2.25E-04 | 8.79E-02 |
| 14706 | Gng4 | mmu-miR-204-5p | 0.60 | 2.74E-04 | 9.28E-02 |
| 20254 | Scg2 | mmu-miR-204-5p | 0.63 | 1.00E-04 | 8.79E-02 |
| 239217 | Kctd12 | mmu-miR-204-5p | 0.60 | 2.79E-04 | 9.28E-02 |
| 26401 | Map3k1 | mmu-miR-204-5p | 0.63 | 1.17E-04 | 8.79E-02 |
| 19699 | Reln | mmu-miR-204-5p | 0.62 | 1.44E-04 | 8.79E-02 |
| 60425 | Doc2g | mmu-miR-204-5p | 0.66 | 4.23E-05 | 7.84E-02 |
| 13390 | Dlx1 | mmu-miR-204-5p | 0.62 | 1.65E-04 | 8.79E-02 |
| 107976 | Bre | mmu-miR-204-5p | -0.65 | 6.61E-05 | 7.84E-02 |
| 140723 | Cacng5 | mmu-miR-204-5p | 0.63 | 1.18E-04 | 8.79E-02 |
| 102657 | Cd276 | mmu-miR-221-3p | -0.70 | 7.14E-06 | 5.93E-02 |
| 20910 | Stxbp1 | mmu-miR-340-5p | 0.70 | 7.92E-06 | 6.20E-02 |
| 11769 | Ap1s1 | mmu-miR-340-5p | 0.69 | 1.49E-05 | 6.20E-02 |
| 57740 | Stk32c | mmu-miR-93-5p | -0.57 | 7.47E-04 | 8.60E-02 |
| 266781 | Snx17 | mmu-miR-93-5p | 0.58 | 5.32E-04 | 8.01E-02 |
| 218194 | Phactr1 | mmu-miR-93-5p | 0.56 | 8.35E-04 | 8.60E-02 |
| 11461 | Actb | mmu-miR-93-5p | 0.55 | 1.01E-03 | 9.00E-02 |
| 54616 | Extl3 | mmu-miR-93-5p | -0.55 | 1.24E-03 | 9.56E-02 |
| 78928 | Pigt | mmu-miR-93-5p | -0.65 | 6.59E-05 | 4.21E-02 |
| 242773 | Slc45a1 | mmu-miR-93-5p | -0.66 | 3.76E-05 | 3.71E-02 |
| 20525 | Slc2a1 | mmu-miR-93-5p | -0.59 | 3.57E-04 | 6.61E-02 |
| 235431 | Coro2b | mmu-miR-93-5p | -0.56 | 7.98E-04 | 8.60E-02 |
| 18190 | Nrxn2 | mmu-miR-93-5p | -0.57 | 6.90E-04 | 8.50E-02 |
| 14086 | Fscn1 | mmu-miR-93-5p | -0.57 | 5.91E-04 | 8.01E-02 |
| 52915 | Zmiz2 | mmu-miR-93-5p | -0.56 | 8.32E-04 | 8.60E-02 |
| 71770 | Ap2b1 | mmu-miR-93-5p | -0.61 | 1.94E-04 | 5.70E-02 |
| 20779 | Src | mmu-miR-93-5p | -0.55 | 1.17E-03 | 9.56E-02 |
| 13855 | Epn2 | mmu-miR-93-5p | 0.56 | 9.58E-04 | 8.83E-02 |
| 75751 | Ipo4 | mmu-miR-93-5p | -0.72 | 3.85E-06 | 8.00E-03 |
| 11932 | Atp1b2 | mmu-miR-93-5p | -0.63 | 1.06E-04 | 5.34E-02 |
| 333331 | LOC333331 | mmu-miR-93-5p | 0.55 | 9.82E-04 | 8.96E-02 |
| 26562 | Ncdn | mmu-miR-93-5p | -0.56 | 8.46E-04 | 8.60E-02 |
| 99010 | Lpcat4 | mmu-miR-93-5p | -0.60 | 2.61E-04 | 6.16E-02 |
| 19025 | Ctsa | mmu-miR-93-5p | 0.61 | 2.15E-04 | 5.76E-02 |
| 21807 | Tsc22d1 | mmu-miR-93-5p | 0.54 | 1.31E-03 | 9.72E-02 |
| 53379 | Hnrnpa2b1 | mmu-miR-93-5p | 0.62 | 1.65E-04 | 5.70E-02 |
| 432763 | Prr7 | mmu-miR-93-5p | -0.63 | 1.01E-04 | 5.34E-02 |
| 22278 | Usf1 | mmu-miR-93-5p | 0.54 | 1.34E-03 | 9.79E-02 |
| 229473 | D930015E06Rik | mmu-miR-93-5p | -0.62 | 1.69E-04 | 5.70E-02 |
| 224997 | Dlgap1 | mmu-miR-93-5p | 0.58 | 5.41E-04 | 8.01E-02 |
| 276770 | Eif5a | mmu-miR-93-5p | 0.63 | 1.22E-04 | 5.34E-02 |
| 94232 | Ubqln4 | mmu-miR-93-5p | -0.57 | 6.59E-04 | 8.46E-02 |
| 76898 | B3gat1 | mmu-miR-93-5p | -0.65 | 5.36E-05 | 3.71E-02 |
| 217866 | Cdc42bpb | mmu-miR-93-5p | -0.57 | 5.81E-04 | 8.01E-02 |
| 76820 | D12Ertd553e | mmu-miR-93-5p | 0.55 | 1.23E-03 | 9.56E-02 |
| 17299 | Mettl1 | mmu-miR-93-5p | -0.56 | 9.35E-04 | 8.73E-02 |
| 20692 | Sparc | mmu-miR-93-5p | -0.56 | 8.27E-04 | 8.60E-02 |
| 18642 | Pfkm | mmu-miR-93-5p | 0.59 | 3.58E-04 | 6.61E-02 |
| 11518 | Add1 | mmu-miR-93-5p | 0.54 | 1.28E-03 | 9.70E-02 |
| 319477 | 6030419C18Rik | mmu-miR-93-5p | -0.59 | 3.94E-04 | 6.96E-02 |
| 20743 | Spnb3 | mmu-miR-93-5p | -0.55 | 1.01E-03 | 9.00E-02 |
| 100046136 | LOC100046136 | mmu-miR-93-5p | 0.67 | 2.34E-05 | 3.24E-02 |
| 225642 | Grp | mmu-miR-93-5p | -0.57 | 6.95E-04 | 8.50E-02 |
| 236539 | Phgdh | mmu-miR-93-5p | 0.58 | 5.27E-04 | 8.01E-02 |
| 22404 | Wiz | mmu-miR-93-5p | -0.56 | 9.18E-04 | 8.73E-02 |
| 12297 | Cacnb3 | mmu-miR-93-5p | -0.56 | 8.96E-04 | 8.73E-02 |
| 74204 | Xpo6 | mmu-miR-93-5p | -0.55 | 1.12E-03 | 9.49E-02 |
| 66513 | Map3k7ip1 | mmu-miR-93-5p | -0.64 | 7.16E-05 | 4.24E-02 |
| 229615 | Pias3 | mmu-miR-93-5p | -0.61 | 1.99E-04 | 5.70E-02 |
| 21858 | Timp2 | mmu-miR-93-5p | -0.60 | 3.26E-04 | 6.61E-02 |
| 20887 | Sult1a1 | mmu-miR-93-5p | -0.57 | 7.06E-04 | 8.50E-02 |
| 13628 | Eef1a2 | mmu-miR-93-5p | 0.57 | 6.02E-04 | 8.01E-02 |
| 71947 | 2310067B10Rik | mmu-miR-93-5p | -0.62 | 1.73E-04 | 5.70E-02 |
| 545554 | Ankrd34a | mmu-miR-93-5p | -0.60 | 3.04E-04 | 6.61E-02 |
| 100046744 | LOC100046744 | mmu-miR-93-5p | -0.72 | 3.51E-06 | 8.00E-03 |
| 17318 | Mid1 | mmu-miR-93-5p | -0.55 | 1.15E-03 | 9.53E-02 |
| 71916 | Dus4l | mmu-miR-93-5p | -0.56 | 7.89E-04 | 8.60E-02 |
| 228355 | Madd | mmu-miR-93-5p | 0.56 | 8.49E-04 | 8.60E-02 |
| 211548 | Nomo1 | mmu-miR-93-5p | -0.57 | 7.01E-04 | 8.50E-02 |
| 66366 | Ergic3 | mmu-miR-93-5p | 0.56 | 8.45E-04 | 8.60E-02 |
| 14661 | Lgsn | mmu-miR-93-5p | 0.56 | 8.93E-04 | 8.73E-02 |
| 229517 | Slc25a44 | mmu-miR-93-5p | -0.63 | 1.22E-04 | 5.34E-02 |
| 52040 | Ppp1r10 | mmu-miR-93-5p | -0.63 | 1.18E-04 | 5.34E-02 |
| 14229 | Fkbp5 | mmu-miR-93-5p | -0.62 | 1.79E-04 | 5.70E-02 |
| 219022 | Ttc5 | mmu-miR-93-5p | 0.55 | 1.24E-03 | 9.56E-02 |
| 240753 | Plekha6 | mmu-miR-93-5p | 0.57 | 5.83E-04 | 8.01E-02 |
| 69719 | Cad | mmu-miR-93-5p | -0.61 | 2.07E-04 | 5.74E-02 |
| 70025 | Acot7 | mmu-miR-93-5p | 0.62 | 1.72E-04 | 5.70E-02 |
| 70510 | Rnf167 | mmu-miR-93-5p | 0.56 | 8.04E-04 | 8.60E-02 |
| 100044363 | LOC100044363 | mmu-miR-93-5p | 0.55 | 1.24E-03 | 9.56E-02 |
| 233900 | Rnf40 | mmu-miR-93-5p | -0.58 | 4.99E-04 | 8.01E-02 |
| 30853 | Mlf2 | mmu-miR-93-5p | 0.54 | 1.31E-03 | 9.72E-02 |
| 21429 | Ubtf | mmu-miR-93-5p | -0.58 | 4.74E-04 | 7.87E-02 |
| 71472 | Usp19 | mmu-miR-93-5p | -0.55 | 1.20E-03 | 9.56E-02 |
| 100044204 | LOC100044204 | mmu-miR-93-5p | -0.54 | 1.27E-03 | 9.64E-02 |
| 434858 | EG434858 | mmu-miR-93-5p | 0.56 | 8.15E-04 | 8.60E-02 |
| 110147 | Ehmt2 | mmu-miR-93-5p | -0.54 | 1.38E-03 | 9.86E-02 |
| 100045617 | LOC100045617 | mmu-miR-93-5p | 0.55 | 1.05E-03 | 9.12E-02 |
| 241846 | Lsm14b | mmu-miR-93-5p | -0.66 | 4.73E-05 | 3.71E-02 |
| 23859 | Dlg2 | mmu-miR-93-5p | -0.60 | 2.92E-04 | 6.54E-02 |
| 20810 | Srm | mmu-miR-93-5p | 0.65 | 5.27E-05 | 3.71E-02 |
| 230857 | Ece1 | mmu-miR-93-5p | -0.54 | 1.41E-03 | 9.98E-02 |
| 103711 | Pnpo | mmu-miR-93-5p | 0.58 | 5.40E-04 | 8.01E-02 |
| 329777 | Pigk | mmu-miR-93-5p | 0.59 | 3.47E-04 | 6.61E-02 |
| 213742 | Xist | mmu-miR-93-5p | -0.61 | 1.86E-04 | 5.70E-02 |
| 72029 | Cnpy3 | mmu-miR-93-5p | 0.55 | 1.05E-03 | 9.12E-02 |
| 71732 | Vps11 | mmu-miR-93-5p | -0.60 | 2.67E-04 | 6.16E-02 |
| 53334 | Gosr1 | mmu-miR-93-5p | -0.57 | 6.08E-04 | 8.01E-02 |
| 26949 | Vat1 | mmu-miR-93-5p | -0.55 | 1.10E-03 | 9.40E-02 |
| 93685 | Entpd7 | mmu-miR-93-5p | 0.55 | 1.15E-03 | 9.53E-02 |
| 12048 | Bcl2l1 | mmu-miR-93-5p | 0.57 | 6.63E-04 | 8.46E-02 |
| 27096 | Trappc3 | mmu-miR-93-5p | 0.61 | 2.42E-04 | 6.08E-02 |
| 100732 | Mapre3 | mmu-miR-93-5p | -0.58 | 5.17E-04 | 8.01E-02 |
| 13385 | Dlg4 | mmu-miR-93-5p | -0.56 | 7.58E-04 | 8.60E-02 |
| 102657 | Cd276 | mmu-miR-93-5p | -0.68 | 1.81E-05 | 3.01E-02 |
| 57370 | B4galt3 | mmu-miR-93-5p | 0.60 | 3.28E-04 | 6.61E-02 |
| 30934 | Tor1b | mmu-miR-93-5p | -0.59 | 3.55E-04 | 6.61E-02 |
| 67016 | Tbc1d2b | mmu-miR-93-5p | -0.56 | 8.21E-04 | 8.60E-02 |
| 432486 | Gnptab | mmu-miR-93-5p | -0.56 | 9.14E-04 | 8.73E-02 |
| 240025 | Dact2 | mmu-miR-93-5p | -0.55 | 1.23E-03 | 9.56E-02 |
| 16834 | Cog1 | mmu-miR-93-5p | -0.58 | 5.65E-04 | 8.01E-02 |
| 13384 | Mpp3 | mmu-miR-93-5p | 0.62 | 1.63E-04 | 5.70E-02 |
| 11993 | Aup1 | mmu-miR-93-5p | 0.76 | 3.76E-07 | 3.12E-03 |
| 270058 | Mtap1s | mmu-miR-93-5p | -0.56 | 8.62E-04 | 8.63E-02 |
| 68525 | Evc2 | mmu-miR-93-5p | -0.59 | 3.50E-04 | 6.61E-02 |
| 243616 | Slc6a11 | mmu-miR-93-5p | -0.54 | 1.33E-03 | 9.74E-02 |
| 14102 | Fas | mmu-miR-93-5p | -0.56 | 9.33E-04 | 8.73E-02 |
| 594844 | Tceal3 | mmu-miR-93-5p | 0.59 | 4.36E-04 | 7.39E-02 |
| 66158 | Cxx1a | mmu-miR-93-5p | 0.54 | 1.37E-03 | 9.86E-02 |
| 72895 | Setd5 | mmu-miR-93-5p | -0.72 | 2.99E-06 | 8.00E-03 |
| 56055 | Gtpbp2 | mmu-miR-93-5p | 0.61 | 2.29E-04 | 5.94E-02 |
| 53602 | Hpcal1 | mmu-miR-93-5p | 0.59 | 3.94E-04 | 6.96E-02 |
| 13831 | Epc1 | mmu-miR-93-5p | 0.59 | 4.11E-04 | 7.11E-02 |
| 26987 | Eif4e2 | mmu-miR-93-5p | 0.66 | 4.58E-05 | 3.71E-02 |
| 17760 | Mtap6 | mmu-miR-93-5p | 0.57 | 6.06E-04 | 8.01E-02 |
| 76459 | Car12 | mmu-miR-93-5p | -0.55 | 1.03E-03 | 9.12E-02 |
| 217232 | Cdc27 | mmu-miR-93-5p | -0.55 | 1.19E-03 | 9.56E-02 |
| 100048299 | LOC100048299 | mmu-miR-93-5p | 0.62 | 1.40E-04 | 5.70E-02 |
| 13116 | Cyp46a1 | mmu-miR-93-5p | 0.67 | 2.83E-05 | 3.35E-02 |
| 11682 | Alk | mmu-miR-93-5p | -0.60 | 2.55E-04 | 6.16E-02 |
| 56433 | Vps29 | mmu-miR-676-3p | -0.73 | 1.74E-06 | 1.44E-02 |
| 20910 | Stxbp1 | mmu-miR-376a-3p | 0.71 | 5.56E-06 | 2.31E-02 |
| 11769 | Ap1s1 | mmu-miR-376a-3p | 0.72 | 2.88E-06 | 2.31E-02 |
| 20910 | Stxbp1 | mmu-miR-24-1-5p | 0.71 | 4.43E-06 | 3.68E-02 |
| 14661 | Lgsn | mmu-miR-24-1-5p | 0.65 | 5.59E-05 | 9.20E-02 |
| 11769 | Ap1s1 | mmu-miR-24-1-5p | 0.65 | 6.73E-05 | 9.20E-02 |
| 231148 | Ablim2 | mmu-miR-24-1-5p | 0.65 | 6.24E-05 | 9.20E-02 |
| 27643 | Ubl4 | mmu-miR-24-1-5p | 0.66 | 4.65E-05 | 9.20E-02 |
| 17760 | Mtap6 | mmu-miR-24-1-5p | 0.66 | 3.35E-05 | 9.20E-02 |
| 66642 | Ctnnbl1 | mmu-miR-24-1-5p | -0.63 | 9.56E-05 | 9.92E-02 |
| 27407 | Abcf2 | mmu-miR-24-1-5p | 0.64 | 7.76E-05 | 9.20E-02 |
| 98404 | AI597479 | mmu-miR-181a-5p | 0.70 | 1.00E-05 | 8.30E-02 |
| 13618 | Ednrb | mmu-miR-183-5p | 0.63 | 9.57E-05 | 1.44E-02 |
| 13193 | Dcx | mmu-miR-183-5p | 0.58 | 5.10E-04 | 4.33E-02 |
| 14199 | Fhl1 | mmu-miR-183-5p | 0.62 | 1.34E-04 | 1.76E-02 |
| 83436 | Plekha2 | mmu-miR-183-5p | 0.57 | 7.24E-04 | 5.44E-02 |
| 12891 | Cpne6 | mmu-miR-183-5p | 0.63 | 1.07E-04 | 1.54E-02 |
| 58802 | Kcnmb4 | mmu-miR-183-5p | -0.61 | 2.20E-04 | 2.48E-02 |
| 245857 | Ssh3 | mmu-miR-183-5p | -0.58 | 5.57E-04 | 4.58E-02 |
| 20014 | Rpn2 | mmu-miR-183-5p | 0.67 | 2.69E-05 | 5.73E-03 |
| 70351 | Ppp4r1 | mmu-miR-183-5p | 0.59 | 4.13E-04 | 3.73E-02 |
| 14415 | Gad1 | mmu-miR-183-5p | 0.76 | 3.79E-07 | 2.42E-04 |
| 72265 | Tram1 | mmu-miR-183-5p | 0.54 | 1.37E-03 | 8.13E-02 |
| 76974 | 1190003J15Rik | mmu-miR-183-5p | -0.56 | 7.97E-04 | 5.80E-02 |
| 100019 | Mdn1 | mmu-miR-183-5p | 0.63 | 1.29E-04 | 1.75E-02 |
| 107448 | Unc5a | mmu-miR-183-5p | -0.57 | 6.49E-04 | 5.13E-02 |
| 68273 | Pomgnt1 | mmu-miR-183-5p | -0.63 | 9.84E-05 | 1.46E-02 |
| 11514 | Adcy8 | mmu-miR-183-5p | 0.65 | 5.00E-05 | 9.03E-03 |
| 210711 | 1110007A13Rik | mmu-miR-183-5p | 0.55 | 9.88E-04 | 6.57E-02 |
| 217125 | Samd14 | mmu-miR-183-5p | 0.67 | 2.98E-05 | 6.18E-03 |
| 225849 | Ppp2r5b | mmu-miR-183-5p | -0.66 | 3.84E-05 | 7.58E-03 |
| 22225 | Usp5 | mmu-miR-183-5p | -0.60 | 2.67E-04 | 2.77E-02 |
| 320299 | Iqcb1 | mmu-miR-183-5p | 0.53 | 1.72E-03 | 9.46E-02 |
| 14537 | Gcnt1 | mmu-miR-183-5p | 0.59 | 3.76E-04 | 3.55E-02 |
| 14664 | Slc6a9 | mmu-miR-183-5p | 0.64 | 8.47E-05 | 1.33E-02 |
| 59035 | Carm1 | mmu-miR-183-5p | -0.56 | 8.99E-04 | 6.27E-02 |
| 67971 | Tppp3 | mmu-miR-183-5p | -0.57 | 7.17E-04 | 5.44E-02 |
| 216345 | Zfc3h1 | mmu-miR-183-5p | 0.65 | 4.78E-05 | 8.81E-03 |
| 234776 | Atmin | mmu-miR-183-5p | -0.57 | 5.99E-04 | 4.83E-02 |
| 140919 | Slc17a6 | mmu-miR-183-5p | 0.54 | 1.37E-03 | 8.13E-02 |
| 22187 | Ubb | mmu-miR-183-5p | 0.54 | 1.54E-03 | 8.95E-02 |
| 18111 | Nnat | mmu-miR-183-5p | 0.54 | 1.41E-03 | 8.30E-02 |
| 22348 | Slc32a1 | mmu-miR-183-5p | 0.63 | 1.18E-04 | 1.65E-02 |
| 14706 | Gng4 | mmu-miR-183-5p | 0.72 | 3.58E-06 | 1.35E-03 |
| 53599 | Cd164 | mmu-miR-183-5p | 0.60 | 2.87E-04 | 2.81E-02 |
| 20254 | Scg2 | mmu-miR-183-5p | 0.58 | 4.49E-04 | 3.95E-02 |
| 75104 | Mmd2 | mmu-miR-183-5p | 0.53 | 1.74E-03 | 9.46E-02 |
| 239217 | Kctd12 | mmu-miR-183-5p | 0.67 | 2.69E-05 | 5.73E-03 |
| 105859 | Csdc2 | mmu-miR-183-5p | 0.75 | 7.09E-07 | 4.20E-04 |
| 72585 | Lypd1 | mmu-miR-183-5p | -0.53 | 1.77E-03 | 9.46E-02 |
| 29861 | Dpf1 | mmu-miR-183-5p | -0.55 | 1.02E-03 | 6.65E-02 |
| 56699 | Cdc42ep4 | mmu-miR-183-5p | 0.57 | 6.41E-04 | 5.12E-02 |
| 12362 | Casp1 | mmu-miR-183-5p | 0.70 | 9.33E-06 | 2.67E-03 |
| 12111 | Bgn | mmu-miR-183-5p | 0.68 | 2.06E-05 | 4.89E-03 |
| 20454 | St3gal5 | mmu-miR-183-5p | -0.55 | 1.21E-03 | 7.42E-02 |
| 14160 | Lgr5 | mmu-miR-183-5p | 0.53 | 1.66E-03 | 9.35E-02 |
| 15360 | Hmgcs2 | mmu-miR-183-5p | 0.58 | 4.79E-04 | 4.14E-02 |
| 11749 | Anxa6 | mmu-miR-183-5p | -0.56 | 8.34E-04 | 6.02E-02 |
| 11933 | Atp1b3 | mmu-miR-183-5p | 0.60 | 2.74E-04 | 2.77E-02 |
| 68801 | Elovl5 | mmu-miR-183-5p | 0.64 | 8.94E-05 | 1.37E-02 |
| 26401 | Map3k1 | mmu-miR-183-5p | 0.72 | 3.20E-06 | 1.29E-03 |
| 12308 | Calb2 | mmu-miR-183-5p | 0.84 | 1.35E-09 | 3.78E-06 |
| 14115 | Fbln2 | mmu-miR-183-5p | 0.59 | 3.86E-04 | 3.60E-02 |
| 20932 | Surf4 | mmu-miR-183-5p | 0.55 | 1.14E-03 | 7.18E-02 |
| 16206 | Lrig1 | mmu-miR-183-5p | 0.58 | 4.52E-04 | 3.95E-02 |
| 232441 | Rerg | mmu-miR-183-5p | 0.71 | 6.46E-06 | 2.06E-03 |
| 233552 | Gdpd5 | mmu-miR-183-5p | 0.56 | 9.69E-04 | 6.57E-02 |
| 68738 | Acss1 | mmu-miR-183-5p | 0.55 | 1.15E-03 | 7.18E-02 |
| 17268 | Meis1 | mmu-miR-183-5p | 0.73 | 2.59E-06 | 1.19E-03 |
| 245386 | 6430550H21Rik | mmu-miR-183-5p | 0.69 | 1.49E-05 | 3.86E-03 |
| 19699 | Reln | mmu-miR-183-5p | 0.78 | 1.51E-07 | 1.57E-04 |
| 70762 | Dclk2 | mmu-miR-183-5p | 0.57 | 7.13E-04 | 5.44E-02 |
| 13179 | Dcn | mmu-miR-183-5p | 0.53 | 1.62E-03 | 9.22E-02 |
| 74020 | Cpne4 | mmu-miR-183-5p | 0.68 | 1.77E-05 | 4.46E-03 |
| 99738 | Kcnc4 | mmu-miR-183-5p | -0.56 | 9.73E-04 | 6.57E-02 |
| 17110 | Lyz | mmu-miR-183-5p | 0.60 | 2.71E-04 | 2.77E-02 |
| 50771 | Atp9b | mmu-miR-183-5p | -0.60 | 2.51E-04 | 2.71E-02 |
| 320333 | D830030K20Rik | mmu-miR-183-5p | -0.55 | 1.15E-03 | 7.18E-02 |
| 60425 | Doc2g | mmu-miR-183-5p | 0.82 | 1.30E-08 | 2.16E-05 |
| 227325 | Dner | mmu-miR-183-5p | 0.60 | 2.81E-04 | 2.81E-02 |
| 78593 | Nrip3 | mmu-miR-183-5p | 0.64 | 7.04E-05 | 1.15E-02 |
| 13390 | Dlx1 | mmu-miR-183-5p | 0.80 | 3.73E-08 | 4.43E-05 |
| 170574 | Sp7 | mmu-miR-183-5p | 0.72 | 3.27E-06 | 1.29E-03 |
| 14252 | Flot2 | mmu-miR-183-5p | -0.54 | 1.59E-03 | 9.15E-02 |
| 170677 | Pcdh21 | mmu-miR-183-5p | 0.84 | 1.37E-09 | 3.78E-06 |
| 100047353 | LOC100047353 | mmu-miR-183-5p | 0.70 | 8.98E-06 | 2.67E-03 |
| 72102 | Dusp11 | mmu-miR-183-5p | 0.57 | 7.28E-04 | 5.44E-02 |
| 67839 | Gpsm1 | mmu-miR-183-5p | 0.68 | 2.13E-05 | 4.91E-03 |
| 18829 | Ccl21a | mmu-miR-183-5p | -0.61 | 2.25E-04 | 2.49E-02 |
| 93695 | Gpnmb | mmu-miR-183-5p | 0.56 | 8.85E-04 | 6.23E-02 |
| 60510 | Syt9 | mmu-miR-183-5p | 0.60 | 3.12E-04 | 3.01E-02 |
| 140723 | Cacng5 | mmu-miR-183-5p | 0.74 | 1.01E-06 | 5.40E-04 |
| 12226 | Btg1 | mmu-miR-183-5p | 0.84 | 2.47E-09 | 5.13E-06 |
| 15081 | H3f3b | mmu-miR-183-5p | 0.65 | 5.30E-05 | 9.37E-03 |
| 22773 | Zic3 | mmu-miR-183-5p | 0.55 | 1.01E-03 | 6.65E-02 |
| 209086 | Samd9l | mmu-miR-183-5p | 0.66 | 4.55E-05 | 8.78E-03 |
| 50793 | Orc3l | mmu-miR-183-5p | 0.71 | 4.53E-06 | 1.57E-03 |
| 100047583 | LOC100047583 | mmu-miR-183-5p | 0.78 | 1.85E-07 | 1.70E-04 |
| 319180 | Hist1h2bf | mmu-miR-183-5p | 0.66 | 3.32E-05 | 6.72E-03 |
| 214189 | Scgn | mmu-miR-183-5p | 0.77 | 3.30E-07 | 2.29E-04 |
| 18516 | Pbx3 | mmu-miR-183-5p | 0.57 | 6.82E-04 | 5.34E-02 |
| 231086 | Hadhb | mmu-miR-183-5p | 0.63 | 1.19E-04 | 1.65E-02 |
| 70536 | Qpct | mmu-miR-183-5p | 0.63 | 1.07E-04 | 1.54E-02 |
| 22044 | Trh | mmu-miR-183-5p | 0.88 | 2.25E-11 | 1.87E-07 |
| 114255 | Dok4 | mmu-miR-183-5p | 0.56 | 9.75E-04 | 6.57E-02 |
| 320145 | Sp8 | mmu-miR-183-5p | 0.74 | 1.04E-06 | 5.40E-04 |
| 13446 | Doc2a | mmu-miR-183-5p | -0.62 | 1.32E-04 | 1.76E-02 |
| 235599 | 6430571L13Rik | mmu-miR-183-5p | 0.53 | 1.68E-03 | 9.45E-02 |
| 14617 | Gjd2 | mmu-miR-183-5p | 0.57 | 6.99E-04 | 5.42E-02 |
| 96979 | Ptges2 | mmu-miR-183-5p | -0.59 | 3.64E-04 | 3.47E-02 |
| 22421 | Wnt7a | mmu-miR-183-5p | 0.62 | 1.50E-04 | 1.89E-02 |
| 12122 | Bid | mmu-miR-183-5p | 0.62 | 1.65E-04 | 2.02E-02 |
| 66659 | Acp6 | mmu-miR-183-5p | -0.53 | 1.74E-03 | 9.46E-02 |
| 12988 | Csk | mmu-miR-183-5p | 0.58 | 5.11E-04 | 4.33E-02 |
| 59093 | Pcbp3 | mmu-miR-183-5p | 0.61 | 2.21E-04 | 2.48E-02 |
| 213391 | Rassf4 | mmu-miR-183-5p | 0.58 | 5.75E-04 | 4.68E-02 |
| 17536 | Meis2 | mmu-miR-183-5p | 0.54 | 1.47E-03 | 8.61E-02 |
| 243328 | Slc29a4 | mmu-miR-183-5p | 0.53 | 1.73E-03 | 9.46E-02 |
| 12836 | Col7a1 | mmu-miR-183-5p | 0.55 | 1.14E-03 | 7.18E-02 |
| 21753 | Tes | mmu-miR-183-5p | 0.55 | 1.03E-03 | 6.71E-02 |
| 545279 | E530011F12Rik | mmu-miR-183-5p | 0.81 | 2.17E-08 | 3.00E-05 |
| 64075 | Smoc1 | mmu-miR-183-5p | 0.65 | 6.41E-05 | 1.09E-02 |
| 20317 | Serpinf1 | mmu-miR-183-5p | 0.55 | 1.21E-03 | 7.42E-02 |
| 76281 | Tax1bp3 | mmu-miR-183-5p | 0.60 | 2.68E-04 | 2.77E-02 |
| 20199 | S100a5 | mmu-miR-183-5p | 0.77 | 2.68E-07 | 2.02E-04 |
| 15312 | Hmgn1 | mmu-miR-183-5p | 0.65 | 5.58E-05 | 9.66E-03 |
| 20750 | Spp1 | mmu-miR-183-5p | 0.72 | 3.26E-06 | 1.29E-03 |
| 72075 | Ogfr | mmu-miR-183-5p | 0.59 | 4.05E-04 | 3.70E-02 |
| 238266 | Syt16 | mmu-miR-183-5p | -0.61 | 1.84E-04 | 2.21E-02 |
| 58875 | Hibadh | mmu-miR-183-5p | 0.68 | 1.89E-05 | 4.62E-03 |
| 13194 | Ddb1 | mmu-miR-183-5p | -0.55 | 1.22E-03 | 7.42E-02 |
| 574403 | EG574403 | mmu-miR-183-5p | 0.65 | 6.74E-05 | 1.12E-02 |
| 12404 | Cbln1 | mmu-miR-183-5p | 0.77 | 2.08E-07 | 1.73E-04 |
| 105844 | Card10 | mmu-miR-183-5p | 0.61 | 1.86E-04 | 2.21E-02 |
| 320736 | E130203B14Rik | mmu-miR-183-5p | 0.61 | 2.21E-04 | 2.48E-02 |
| 14261 | Fmo1 | mmu-miR-183-5p | 0.70 | 9.07E-06 | 2.67E-03 |
| 68836 | Mrpl52 | mmu-miR-183-5p | 0.54 | 1.30E-03 | 7.90E-02 |
| 110253 | Triobp | mmu-miR-183-5p | 0.57 | 7.34E-04 | 5.44E-02 |
| 103712 | 6330403K07Rik | mmu-miR-183-5p | 0.60 | 2.88E-04 | 2.81E-02 |
| 74194 | Rnd3 | mmu-miR-183-5p | 0.56 | 8.49E-04 | 6.07E-02 |
| 66953 | Cdca7 | mmu-miR-183-5p | 0.58 | 5.17E-04 | 4.34E-02 |
| 13730 | Emp1 | mmu-miR-183-5p | 0.62 | 1.47E-04 | 1.88E-02 |
| 66704 | Rbm4b | mmu-miR-183-5p | 0.67 | 2.42E-05 | 5.43E-03 |
| 13392 | Dlx2 | mmu-miR-183-5p | 0.56 | 8.66E-04 | 6.14E-02 |
| 105827 | Amigo2 | mmu-miR-183-5p | 0.55 | 9.83E-04 | 6.57E-02 |
| 170625 | Snx18 | mmu-miR-183-5p | 0.56 | 9.54E-04 | 6.57E-02 |
| 15478 | Hs3st3a1 | mmu-miR-183-5p | 0.61 | 2.02E-04 | 2.36E-02 |
| 237759 | Col23a1 | mmu-miR-183-5p | 0.53 | 1.62E-03 | 9.22E-02 |
| 68087 | Dcakd | mmu-miR-183-5p | 0.58 | 5.47E-04 | 4.54E-02 |
| 101437 | Dhx32 | mmu-miR-183-5p | 0.69 | 1.34E-05 | 3.58E-03 |
| 211949 | Spsb4 | mmu-miR-183-5p | 0.64 | 7.71E-05 | 1.23E-02 |
| 319613 | 5730410E15Rik | mmu-miR-183-5p | 0.53 | 1.76E-03 | 9.46E-02 |
| 21823 | Th | mmu-miR-183-5p | 0.74 | 1.65E-06 | 8.04E-04 |
| 14609 | Gja1 | mmu-miR-183-5p | 0.60 | 2.68E-04 | 2.77E-02 |
| 15214 | Hey2 | mmu-miR-183-5p | 0.62 | 1.38E-04 | 1.79E-02 |
| 70274 | Ly6g6e | mmu-miR-183-5p | 0.72 | 3.94E-06 | 1.42E-03 |
| 56615 | Mgst1 | mmu-miR-183-5p | 0.59 | 4.00E-04 | 3.69E-02 |
| 68039 | Nmb | mmu-miR-183-5p | 0.62 | 1.54E-04 | 1.90E-02 |
| 22771 | Zic1 | mmu-miR-183-5p | 0.65 | 4.75E-05 | 8.81E-03 |
| 329831 | 4833436C18Rik | mmu-miR-183-5p | -0.55 | 1.10E-03 | 7.09E-02 |
| 319183 | Hist1h2bj | mmu-miR-183-5p | 0.59 | 4.30E-04 | 3.84E-02 |
| 70394 | Kptn | mmu-miR-183-5p | -0.53 | 1.76E-03 | 9.46E-02 |
| 11826 | Aqp1 | mmu-miR-183-5p | 0.71 | 5.96E-06 | 1.98E-03 |
| 11836 | Araf | mmu-miR-183-5p | 0.54 | 1.34E-03 | 8.06E-02 |
| 17171 | Mas1 | mmu-miR-183-5p | -0.61 | 2.31E-04 | 2.52E-02 |
| 69993 | Chn2 | mmu-miR-183-5p | 0.56 | 7.69E-04 | 5.65E-02 |
| 18514 | Pbx1 | mmu-miR-183-5p | 0.69 | 1.07E-05 | 2.95E-03 |
| 13618 | Ednrb | mmu-miR-182-5p | 0.64 | 7.84E-05 | 1.31E-02 |
| 13193 | Dcx | mmu-miR-182-5p | 0.59 | 4.33E-04 | 3.86E-02 |
| 14199 | Fhl1 | mmu-miR-182-5p | 0.63 | 1.24E-04 | 1.72E-02 |
| 83436 | Plekha2 | mmu-miR-182-5p | 0.56 | 8.64E-04 | 5.79E-02 |
| 12891 | Cpne6 | mmu-miR-182-5p | 0.62 | 1.67E-04 | 2.08E-02 |
| 58802 | Kcnmb4 | mmu-miR-182-5p | -0.61 | 2.39E-04 | 2.72E-02 |
| 245857 | Ssh3 | mmu-miR-182-5p | -0.56 | 9.49E-04 | 6.15E-02 |
| 20014 | Rpn2 | mmu-miR-182-5p | 0.64 | 9.04E-05 | 1.34E-02 |
| 70351 | Ppp4r1 | mmu-miR-182-5p | 0.57 | 6.00E-04 | 4.57E-02 |
| 14415 | Gad1 | mmu-miR-182-5p | 0.77 | 2.44E-07 | 2.02E-04 |
| 72265 | Tram1 | mmu-miR-182-5p | 0.55 | 1.12E-03 | 6.97E-02 |
| 76974 | 1190003J15Rik | mmu-miR-182-5p | -0.56 | 8.60E-04 | 5.79E-02 |
| 100019 | Mdn1 | mmu-miR-182-5p | 0.60 | 3.23E-04 | 3.27E-02 |
| 107448 | Unc5a | mmu-miR-182-5p | -0.58 | 5.75E-04 | 4.50E-02 |
| 68273 | Pomgnt1 | mmu-miR-182-5p | -0.62 | 1.78E-04 | 2.17E-02 |
| 11514 | Adcy8 | mmu-miR-182-5p | 0.64 | 8.31E-05 | 1.31E-02 |
| 210711 | 1110007A13Rik | mmu-miR-182-5p | 0.57 | 6.86E-04 | 5.04E-02 |
| 217125 | Samd14 | mmu-miR-182-5p | 0.65 | 5.35E-05 | 1.03E-02 |
| 225849 | Ppp2r5b | mmu-miR-182-5p | -0.66 | 4.53E-05 | 9.40E-03 |
| 22225 | Usp5 | mmu-miR-182-5p | -0.57 | 5.99E-04 | 4.57E-02 |
| 14537 | Gcnt1 | mmu-miR-182-5p | 0.59 | 4.11E-04 | 3.79E-02 |
| 14664 | Slc6a9 | mmu-miR-182-5p | 0.63 | 9.61E-05 | 1.40E-02 |
| 59035 | Carm1 | mmu-miR-182-5p | -0.54 | 1.34E-03 | 7.76E-02 |
| 67971 | Tppp3 | mmu-miR-182-5p | -0.59 | 3.79E-04 | 3.60E-02 |
| 216345 | Zfc3h1 | mmu-miR-182-5p | 0.64 | 8.38E-05 | 1.31E-02 |
| 234776 | Atmin | mmu-miR-182-5p | -0.56 | 8.03E-04 | 5.65E-02 |
| 12632 | Cfl2 | mmu-miR-182-5p | -0.55 | 1.13E-03 | 6.97E-02 |
| 140919 | Slc17a6 | mmu-miR-182-5p | 0.54 | 1.32E-03 | 7.75E-02 |
| 22348 | Slc32a1 | mmu-miR-182-5p | 0.63 | 9.97E-05 | 1.40E-02 |
| 20295 | Ccl17 | mmu-miR-182-5p | -0.56 | 9.43E-04 | 6.15E-02 |
| 14706 | Gng4 | mmu-miR-182-5p | 0.72 | 3.15E-06 | 1.16E-03 |
| 53599 | Cd164 | mmu-miR-182-5p | 0.59 | 4.27E-04 | 3.85E-02 |
| 20254 | Scg2 | mmu-miR-182-5p | 0.58 | 5.38E-04 | 4.35E-02 |
| 75104 | Mmd2 | mmu-miR-182-5p | 0.54 | 1.57E-03 | 8.87E-02 |
| 239217 | Kctd12 | mmu-miR-182-5p | 0.69 | 1.35E-05 | 3.67E-03 |
| 105859 | Csdc2 | mmu-miR-182-5p | 0.74 | 1.52E-06 | 7.69E-04 |
| 29861 | Dpf1 | mmu-miR-182-5p | -0.55 | 1.11E-03 | 6.94E-02 |
| 56699 | Cdc42ep4 | mmu-miR-182-5p | 0.59 | 3.69E-04 | 3.60E-02 |
| 12362 | Casp1 | mmu-miR-182-5p | 0.71 | 6.28E-06 | 2.09E-03 |
| 12111 | Bgn | mmu-miR-182-5p | 0.66 | 3.53E-05 | 7.72E-03 |
| 20454 | St3gal5 | mmu-miR-182-5p | -0.53 | 1.64E-03 | 9.06E-02 |
| 15360 | Hmgcs2 | mmu-miR-182-5p | 0.60 | 3.03E-04 | 3.11E-02 |
| 11749 | Anxa6 | mmu-miR-182-5p | -0.56 | 9.72E-04 | 6.26E-02 |
| 11933 | Atp1b3 | mmu-miR-182-5p | 0.59 | 3.75E-04 | 3.60E-02 |
| 68801 | Elovl5 | mmu-miR-182-5p | 0.64 | 7.71E-05 | 1.31E-02 |
| 26401 | Map3k1 | mmu-miR-182-5p | 0.74 | 1.60E-06 | 7.69E-04 |
| 12308 | Calb2 | mmu-miR-182-5p | 0.84 | 2.76E-09 | 8.80E-06 |
| 14115 | Fbln2 | mmu-miR-182-5p | 0.60 | 2.87E-04 | 2.98E-02 |
| 16206 | Lrig1 | mmu-miR-182-5p | 0.59 | 4.37E-04 | 3.86E-02 |
| 232441 | Rerg | mmu-miR-182-5p | 0.69 | 1.17E-05 | 3.36E-03 |
| 233552 | Gdpd5 | mmu-miR-182-5p | 0.56 | 9.44E-04 | 6.15E-02 |
| 68738 | Acss1 | mmu-miR-182-5p | 0.56 | 7.91E-04 | 5.62E-02 |
| 17268 | Meis1 | mmu-miR-182-5p | 0.73 | 2.31E-06 | 9.59E-04 |
| 245386 | 6430550H21Rik | mmu-miR-182-5p | 0.69 | 1.37E-05 | 3.67E-03 |
| 19699 | Reln | mmu-miR-182-5p | 0.78 | 1.20E-07 | 1.24E-04 |
| 70762 | Dclk2 | mmu-miR-182-5p | 0.57 | 6.58E-04 | 4.93E-02 |
| 13179 | Dcn | mmu-miR-182-5p | 0.55 | 1.04E-03 | 6.62E-02 |
| 74020 | Cpne4 | mmu-miR-182-5p | 0.70 | 9.74E-06 | 2.89E-03 |
| 17110 | Lyz | mmu-miR-182-5p | 0.59 | 3.41E-04 | 3.37E-02 |
| 50771 | Atp9b | mmu-miR-182-5p | -0.58 | 5.32E-04 | 4.35E-02 |
| 320333 | D830030K20Rik | mmu-miR-182-5p | -0.59 | 3.86E-04 | 3.60E-02 |
| 78908 | Igsf3 | mmu-miR-182-5p | 0.55 | 1.16E-03 | 7.05E-02 |
| 60425 | Doc2g | mmu-miR-182-5p | 0.82 | 9.98E-09 | 1.66E-05 |
| 14619 | Gjb2 | mmu-miR-182-5p | 0.53 | 1.74E-03 | 9.37E-02 |
| 227325 | Dner | mmu-miR-182-5p | 0.59 | 3.86E-04 | 3.60E-02 |
| 78593 | Nrip3 | mmu-miR-182-5p | 0.64 | 7.88E-05 | 1.31E-02 |
| 13390 | Dlx1 | mmu-miR-182-5p | 0.80 | 4.38E-08 | 5.20E-05 |
| 170574 | Sp7 | mmu-miR-182-5p | 0.71 | 6.04E-06 | 2.09E-03 |
| 14252 | Flot2 | mmu-miR-182-5p | -0.55 | 1.20E-03 | 7.17E-02 |
| 64297 | Gprc5b | mmu-miR-182-5p | 0.53 | 1.65E-03 | 9.06E-02 |
| 170677 | Pcdh21 | mmu-miR-182-5p | 0.83 | 4.07E-09 | 8.80E-06 |
| 100047353 | LOC100047353 | mmu-miR-182-5p | 0.68 | 1.82E-05 | 4.45E-03 |
| 72102 | Dusp11 | mmu-miR-182-5p | 0.57 | 7.35E-04 | 5.26E-02 |
| 67839 | Gpsm1 | mmu-miR-182-5p | 0.68 | 2.01E-05 | 4.77E-03 |
| 18829 | Ccl21a | mmu-miR-182-5p | -0.62 | 1.68E-04 | 2.08E-02 |
| 93695 | Gpnmb | mmu-miR-182-5p | 0.56 | 8.54E-04 | 5.79E-02 |
| 60510 | Syt9 | mmu-miR-182-5p | 0.61 | 2.44E-04 | 2.74E-02 |
| 140723 | Cacng5 | mmu-miR-182-5p | 0.75 | 6.70E-07 | 3.98E-04 |
| 12226 | Btg1 | mmu-miR-182-5p | 0.83 | 4.24E-09 | 8.80E-06 |
| 15081 | H3f3b | mmu-miR-182-5p | 0.66 | 4.67E-05 | 9.45E-03 |
| 22773 | Zic3 | mmu-miR-182-5p | 0.58 | 5.35E-04 | 4.35E-02 |
| 209086 | Samd9l | mmu-miR-182-5p | 0.65 | 5.32E-05 | 1.03E-02 |
| 50793 | Orc3l | mmu-miR-182-5p | 0.70 | 6.77E-06 | 2.16E-03 |
| 100047583 | LOC100047583 | mmu-miR-182-5p | 0.76 | 3.54E-07 | 2.26E-04 |
| 319180 | Hist1h2bf | mmu-miR-182-5p | 0.66 | 4.09E-05 | 8.71E-03 |
| 214189 | Scgn | mmu-miR-182-5p | 0.76 | 3.53E-07 | 2.26E-04 |
| 18516 | Pbx3 | mmu-miR-182-5p | 0.57 | 6.93E-04 | 5.05E-02 |
| 231086 | Hadhb | mmu-miR-182-5p | 0.62 | 1.37E-04 | 1.83E-02 |
| 70536 | Qpct | mmu-miR-182-5p | 0.63 | 9.92E-05 | 1.40E-02 |
| 22044 | Trh | mmu-miR-182-5p | 0.89 | 1.47E-11 | 1.22E-07 |
| 320145 | Sp8 | mmu-miR-182-5p | 0.74 | 1.33E-06 | 7.38E-04 |
| 13446 | Doc2a | mmu-miR-182-5p | -0.60 | 2.80E-04 | 2.95E-02 |
| 12793 | Cnih | mmu-miR-182-5p | -0.54 | 1.35E-03 | 7.78E-02 |
| 235599 | 6430571L13Rik | mmu-miR-182-5p | 0.53 | 1.64E-03 | 9.06E-02 |
| 14617 | Gjd2 | mmu-miR-182-5p | 0.59 | 3.36E-04 | 3.37E-02 |
| 96979 | Ptges2 | mmu-miR-182-5p | -0.60 | 2.62E-04 | 2.83E-02 |
| 22421 | Wnt7a | mmu-miR-182-5p | 0.65 | 6.07E-05 | 1.12E-02 |
| 12122 | Bid | mmu-miR-182-5p | 0.63 | 1.31E-04 | 1.78E-02 |
| 66659 | Acp6 | mmu-miR-182-5p | -0.56 | 8.17E-04 | 5.70E-02 |
| 12988 | Csk | mmu-miR-182-5p | 0.58 | 5.27E-04 | 4.35E-02 |
| 320487 | Heatr5a | mmu-miR-182-5p | 0.54 | 1.26E-03 | 7.41E-02 |
| 59093 | Pcbp3 | mmu-miR-182-5p | 0.64 | 8.51E-05 | 1.31E-02 |
| 213391 | Rassf4 | mmu-miR-182-5p | 0.58 | 5.44E-04 | 4.35E-02 |
| 17536 | Meis2 | mmu-miR-182-5p | 0.53 | 1.63E-03 | 9.06E-02 |
| 12836 | Col7a1 | mmu-miR-182-5p | 0.55 | 1.18E-03 | 7.10E-02 |
| 21753 | Tes | mmu-miR-182-5p | 0.57 | 7.04E-04 | 5.08E-02 |
| 545279 | E530011F12Rik | mmu-miR-182-5p | 0.81 | 2.19E-08 | 3.03E-05 |
| 64075 | Smoc1 | mmu-miR-182-5p | 0.64 | 9.03E-05 | 1.34E-02 |
| 20317 | Serpinf1 | mmu-miR-182-5p | 0.55 | 1.08E-03 | 6.82E-02 |
| 21859 | Timp3 | mmu-miR-182-5p | 0.54 | 1.49E-03 | 8.53E-02 |
| 76281 | Tax1bp3 | mmu-miR-182-5p | 0.58 | 5.05E-04 | 4.33E-02 |
| 20199 | S100a5 | mmu-miR-182-5p | 0.78 | 1.67E-07 | 1.54E-04 |
| 15312 | Hmgn1 | mmu-miR-182-5p | 0.62 | 1.63E-04 | 2.08E-02 |
| 23956 | Neu2 | mmu-miR-182-5p | -0.54 | 1.57E-03 | 8.87E-02 |
| 20750 | Spp1 | mmu-miR-182-5p | 0.72 | 3.22E-06 | 1.16E-03 |
| 72075 | Ogfr | mmu-miR-182-5p | 0.55 | 1.21E-03 | 7.19E-02 |
| 238266 | Syt16 | mmu-miR-182-5p | -0.62 | 1.60E-04 | 2.08E-02 |
| 58875 | Hibadh | mmu-miR-182-5p | 0.68 | 1.55E-05 | 3.90E-03 |
| 13194 | Ddb1 | mmu-miR-182-5p | -0.57 | 5.80E-04 | 4.50E-02 |
| 574403 | EG574403 | mmu-miR-182-5p | 0.65 | 5.95E-05 | 1.12E-02 |
| 29876 | Clic4 | mmu-miR-182-5p | 0.55 | 1.06E-03 | 6.71E-02 |
| 12404 | Cbln1 | mmu-miR-182-5p | 0.77 | 3.29E-07 | 2.26E-04 |
| 105844 | Card10 | mmu-miR-182-5p | 0.61 | 1.82E-04 | 2.19E-02 |
| 320736 | E130203B14Rik | mmu-miR-182-5p | 0.61 | 1.95E-04 | 2.27E-02 |
| 14261 | Fmo1 | mmu-miR-182-5p | 0.70 | 7.16E-06 | 2.20E-03 |
| 68836 | Mrpl52 | mmu-miR-182-5p | 0.56 | 8.64E-04 | 5.79E-02 |
| 110253 | Triobp | mmu-miR-182-5p | 0.56 | 8.34E-04 | 5.77E-02 |
| 103712 | 6330403K07Rik | mmu-miR-182-5p | 0.58 | 4.52E-04 | 3.95E-02 |
| 74194 | Rnd3 | mmu-miR-182-5p | 0.56 | 9.14E-04 | 6.07E-02 |
| 66953 | Cdca7 | mmu-miR-182-5p | 0.57 | 6.65E-04 | 4.93E-02 |
| 13730 | Emp1 | mmu-miR-182-5p | 0.61 | 1.88E-04 | 2.23E-02 |
| 66704 | Rbm4b | mmu-miR-182-5p | 0.64 | 8.12E-05 | 1.31E-02 |
| 13392 | Dlx2 | mmu-miR-182-5p | 0.58 | 4.63E-04 | 4.00E-02 |
| 105827 | Amigo2 | mmu-miR-182-5p | 0.57 | 6.29E-04 | 4.75E-02 |
| 170625 | Snx18 | mmu-miR-182-5p | 0.55 | 1.15E-03 | 7.03E-02 |
| 15478 | Hs3st3a1 | mmu-miR-182-5p | 0.64 | 8.55E-05 | 1.31E-02 |
| 237759 | Col23a1 | mmu-miR-182-5p | 0.53 | 1.71E-03 | 9.29E-02 |
| 68087 | Dcakd | mmu-miR-182-5p | 0.58 | 5.75E-04 | 4.50E-02 |
| 101437 | Dhx32 | mmu-miR-182-5p | 0.69 | 1.48E-05 | 3.83E-03 |
| 211949 | Spsb4 | mmu-miR-182-5p | 0.64 | 8.23E-05 | 1.31E-02 |
| 21823 | Th | mmu-miR-182-5p | 0.73 | 1.67E-06 | 7.69E-04 |
| 14609 | Gja1 | mmu-miR-182-5p | 0.60 | 2.73E-04 | 2.91E-02 |
| 15214 | Hey2 | mmu-miR-182-5p | 0.60 | 2.55E-04 | 2.82E-02 |
| 70274 | Ly6g6e | mmu-miR-182-5p | 0.73 | 2.21E-06 | 9.59E-04 |
| 56615 | Mgst1 | mmu-miR-182-5p | 0.60 | 2.62E-04 | 2.83E-02 |
| 68039 | Nmb | mmu-miR-182-5p | 0.61 | 2.26E-04 | 2.61E-02 |
| 22771 | Zic1 | mmu-miR-182-5p | 0.67 | 2.71E-05 | 6.25E-03 |
| 329831 | 4833436C18Rik | mmu-miR-182-5p | -0.58 | 5.42E-04 | 4.35E-02 |
| 319183 | Hist1h2bj | mmu-miR-182-5p | 0.59 | 4.17E-04 | 3.81E-02 |
| 11826 | Aqp1 | mmu-miR-182-5p | 0.72 | 3.05E-06 | 1.16E-03 |
| 17171 | Mas1 | mmu-miR-182-5p | -0.62 | 1.45E-04 | 1.91E-02 |
| 69993 | Chn2 | mmu-miR-182-5p | 0.58 | 5.37E-04 | 4.35E-02 |
| 18514 | Pbx1 | mmu-miR-182-5p | 0.67 | 2.97E-05 | 6.66E-03 |
| 57738 | Slc15a2 | mmu-miR-182-5p | 0.53 | 1.66E-03 | 9.06E-02 |
| 76267 | Fads1 | mmu-miR-429-3p | 0.55 | 1.01E-03 | 7.06E-02 |
| 13618 | Ednrb | mmu-miR-429-3p | 0.65 | 6.66E-05 | 1.21E-02 |
| 69195 | Tmem121 | mmu-miR-429-3p | -0.56 | 7.71E-04 | 6.06E-02 |
| 13193 | Dcx | mmu-miR-429-3p | 0.59 | 4.28E-04 | 4.08E-02 |
| 14199 | Fhl1 | mmu-miR-429-3p | 0.63 | 1.13E-04 | 1.74E-02 |
| 12891 | Cpne6 | mmu-miR-429-3p | 0.63 | 1.07E-04 | 1.72E-02 |
| 58802 | Kcnmb4 | mmu-miR-429-3p | -0.60 | 2.67E-04 | 2.99E-02 |
| 140577 | Ankrd6 | mmu-miR-429-3p | 0.55 | 1.08E-03 | 7.24E-02 |
| 245857 | Ssh3 | mmu-miR-429-3p | -0.55 | 1.19E-03 | 7.55E-02 |
| 20014 | Rpn2 | mmu-miR-429-3p | 0.64 | 7.75E-05 | 1.36E-02 |
| 14415 | Gad1 | mmu-miR-429-3p | 0.75 | 6.44E-07 | 5.43E-04 |
| 72265 | Tram1 | mmu-miR-429-3p | 0.61 | 1.81E-04 | 2.30E-02 |
| 76974 | 1190003J15Rik | mmu-miR-429-3p | -0.57 | 7.41E-04 | 5.98E-02 |
| 100019 | Mdn1 | mmu-miR-429-3p | 0.58 | 4.53E-04 | 4.22E-02 |
| 107448 | Unc5a | mmu-miR-429-3p | -0.58 | 4.79E-04 | 4.30E-02 |
| 68273 | Pomgnt1 | mmu-miR-429-3p | -0.64 | 8.13E-05 | 1.38E-02 |
| 11514 | Adcy8 | mmu-miR-429-3p | 0.62 | 1.49E-04 | 2.05E-02 |
| 217125 | Samd14 | mmu-miR-429-3p | 0.65 | 5.64E-05 | 1.11E-02 |
| 225849 | Ppp2r5b | mmu-miR-429-3p | -0.63 | 9.80E-05 | 1.59E-02 |
| 22225 | Usp5 | mmu-miR-429-3p | -0.56 | 8.84E-04 | 6.53E-02 |
| 14537 | Gcnt1 | mmu-miR-429-3p | 0.60 | 3.14E-04 | 3.39E-02 |
| 14664 | Slc6a9 | mmu-miR-429-3p | 0.60 | 2.94E-04 | 3.21E-02 |
| 59035 | Carm1 | mmu-miR-429-3p | -0.54 | 1.32E-03 | 8.18E-02 |
| 67971 | Tppp3 | mmu-miR-429-3p | -0.60 | 2.81E-04 | 3.11E-02 |
| 216345 | Zfc3h1 | mmu-miR-429-3p | 0.66 | 3.53E-05 | 7.72E-03 |
| 12632 | Cfl2 | mmu-miR-429-3p | -0.54 | 1.60E-03 | 9.37E-02 |
| 140919 | Slc17a6 | mmu-miR-429-3p | 0.56 | 9.35E-04 | 6.69E-02 |
| 18111 | Nnat | mmu-miR-429-3p | 0.54 | 1.29E-03 | 8.05E-02 |
| 22348 | Slc32a1 | mmu-miR-429-3p | 0.68 | 2.10E-05 | 5.28E-03 |
| 20295 | Ccl17 | mmu-miR-429-3p | -0.55 | 1.08E-03 | 7.24E-02 |
| 14706 | Gng4 | mmu-miR-429-3p | 0.70 | 8.59E-06 | 3.10E-03 |
| 53599 | Cd164 | mmu-miR-429-3p | 0.56 | 8.82E-04 | 6.53E-02 |
| 20254 | Scg2 | mmu-miR-429-3p | 0.59 | 4.10E-04 | 4.05E-02 |
| 75104 | Mmd2 | mmu-miR-429-3p | 0.61 | 2.02E-04 | 2.46E-02 |
| 239217 | Kctd12 | mmu-miR-429-3p | 0.71 | 4.31E-06 | 2.11E-03 |
| 105859 | Csdc2 | mmu-miR-429-3p | 0.71 | 5.40E-06 | 2.14E-03 |
| 56699 | Cdc42ep4 | mmu-miR-429-3p | 0.57 | 5.90E-04 | 4.95E-02 |
| 12362 | Casp1 | mmu-miR-429-3p | 0.66 | 3.49E-05 | 7.72E-03 |
| 12111 | Bgn | mmu-miR-429-3p | 0.69 | 1.20E-05 | 3.97E-03 |
| 15360 | Hmgcs2 | mmu-miR-429-3p | 0.62 | 1.46E-04 | 2.05E-02 |
| 11749 | Anxa6 | mmu-miR-429-3p | -0.56 | 8.76E-04 | 6.53E-02 |
| 11933 | Atp1b3 | mmu-miR-429-3p | 0.58 | 4.83E-04 | 4.30E-02 |
| 68801 | Elovl5 | mmu-miR-429-3p | 0.61 | 1.83E-04 | 2.30E-02 |
| 26401 | Map3k1 | mmu-miR-429-3p | 0.72 | 2.69E-06 | 1.44E-03 |
| 12308 | Calb2 | mmu-miR-429-3p | 0.84 | 1.97E-09 | 8.17E-06 |
| 14115 | Fbln2 | mmu-miR-429-3p | 0.56 | 7.62E-04 | 6.06E-02 |
| 16206 | Lrig1 | mmu-miR-429-3p | 0.62 | 1.53E-04 | 2.05E-02 |
| 232441 | Rerg | mmu-miR-429-3p | 0.69 | 1.45E-05 | 4.30E-03 |
| 68738 | Acss1 | mmu-miR-429-3p | 0.59 | 3.71E-04 | 3.90E-02 |
| 17268 | Meis1 | mmu-miR-429-3p | 0.71 | 4.97E-06 | 2.12E-03 |
| 245386 | 6430550H21Rik | mmu-miR-429-3p | 0.65 | 5.45E-05 | 1.10E-02 |
| 19699 | Reln | mmu-miR-429-3p | 0.79 | 8.56E-08 | 8.88E-05 |
| 74020 | Cpne4 | mmu-miR-429-3p | 0.68 | 1.76E-05 | 4.70E-03 |
| 17110 | Lyz | mmu-miR-429-3p | 0.60 | 2.48E-04 | 2.86E-02 |
| 50771 | Atp9b | mmu-miR-429-3p | -0.56 | 8.12E-04 | 6.24E-02 |
| 320333 | D830030K20Rik | mmu-miR-429-3p | -0.56 | 9.14E-04 | 6.60E-02 |
| 78908 | Igsf3 | mmu-miR-429-3p | 0.54 | 1.48E-03 | 8.70E-02 |
| 60425 | Doc2g | mmu-miR-429-3p | 0.82 | 8.34E-09 | 1.38E-05 |
| 14619 | Gjb2 | mmu-miR-429-3p | 0.55 | 1.19E-03 | 7.55E-02 |
| 227325 | Dner | mmu-miR-429-3p | 0.55 | 1.02E-03 | 7.07E-02 |
| 78593 | Nrip3 | mmu-miR-429-3p | 0.64 | 8.58E-05 | 1.42E-02 |
| 13390 | Dlx1 | mmu-miR-429-3p | 0.79 | 6.75E-08 | 8.88E-05 |
| 170574 | Sp7 | mmu-miR-429-3p | 0.68 | 1.53E-05 | 4.39E-03 |
| 14252 | Flot2 | mmu-miR-429-3p | -0.54 | 1.38E-03 | 8.32E-02 |
| 170677 | Pcdh21 | mmu-miR-429-3p | 0.83 | 3.14E-09 | 8.69E-06 |
| 100047353 | LOC100047353 | mmu-miR-429-3p | 0.70 | 7.14E-06 | 2.69E-03 |
| 72102 | Dusp11 | mmu-miR-429-3p | 0.62 | 1.70E-04 | 2.24E-02 |
| 67839 | Gpsm1 | mmu-miR-429-3p | 0.65 | 5.12E-05 | 1.06E-02 |
| 18829 | Ccl21a | mmu-miR-429-3p | -0.63 | 1.28E-04 | 1.87E-02 |
| 60510 | Syt9 | mmu-miR-429-3p | 0.59 | 4.19E-04 | 4.08E-02 |
| 140723 | Cacng5 | mmu-miR-429-3p | 0.73 | 2.11E-06 | 1.25E-03 |
| 12226 | Btg1 | mmu-miR-429-3p | 0.82 | 6.71E-09 | 1.38E-05 |
| 15081 | H3f3b | mmu-miR-429-3p | 0.66 | 3.38E-05 | 7.72E-03 |
| 22773 | Zic3 | mmu-miR-429-3p | 0.54 | 1.28E-03 | 8.05E-02 |
| 209086 | Samd9l | mmu-miR-429-3p | 0.64 | 7.84E-05 | 1.36E-02 |
| 50793 | Orc3l | mmu-miR-429-3p | 0.65 | 6.54E-05 | 1.21E-02 |
| 100047583 | LOC100047583 | mmu-miR-429-3p | 0.75 | 6.53E-07 | 5.43E-04 |
| 319180 | Hist1h2bf | mmu-miR-429-3p | 0.69 | 1.25E-05 | 4.00E-03 |
| 214189 | Scgn | mmu-miR-429-3p | 0.74 | 1.46E-06 | 9.33E-04 |
| 18516 | Pbx3 | mmu-miR-429-3p | 0.58 | 4.77E-04 | 4.30E-02 |
| 231086 | Hadhb | mmu-miR-429-3p | 0.62 | 1.53E-04 | 2.05E-02 |
| 70536 | Qpct | mmu-miR-429-3p | 0.57 | 5.88E-04 | 4.95E-02 |
| 22044 | Trh | mmu-miR-429-3p | 0.87 | 1.62E-10 | 1.35E-06 |
| 114255 | Dok4 | mmu-miR-429-3p | 0.56 | 9.63E-04 | 6.77E-02 |
| 320145 | Sp8 | mmu-miR-429-3p | 0.71 | 5.06E-06 | 2.12E-03 |
| 13446 | Doc2a | mmu-miR-429-3p | -0.56 | 7.87E-04 | 6.11E-02 |
| 235599 | 6430571L13Rik | mmu-miR-429-3p | 0.54 | 1.34E-03 | 8.19E-02 |
| 14617 | Gjd2 | mmu-miR-429-3p | 0.56 | 9.08E-04 | 6.60E-02 |
| 96979 | Ptges2 | mmu-miR-429-3p | -0.59 | 4.05E-04 | 4.05E-02 |
| 22421 | Wnt7a | mmu-miR-429-3p | 0.59 | 3.95E-04 | 4.05E-02 |
| 12122 | Bid | mmu-miR-429-3p | 0.59 | 4.02E-04 | 4.05E-02 |
| 66659 | Acp6 | mmu-miR-429-3p | -0.59 | 3.78E-04 | 3.92E-02 |
| 320487 | Heatr5a | mmu-miR-429-3p | 0.56 | 9.43E-04 | 6.69E-02 |
| 59093 | Pcbp3 | mmu-miR-429-3p | 0.63 | 1.13E-04 | 1.74E-02 |
| 17536 | Meis2 | mmu-miR-429-3p | 0.55 | 1.09E-03 | 7.24E-02 |
| 243328 | Slc29a4 | mmu-miR-429-3p | 0.55 | 1.10E-03 | 7.24E-02 |
| 12836 | Col7a1 | mmu-miR-429-3p | 0.57 | 6.78E-04 | 5.57E-02 |
| 545279 | E530011F12Rik | mmu-miR-429-3p | 0.79 | 8.41E-08 | 8.88E-05 |
| 64075 | Smoc1 | mmu-miR-429-3p | 0.61 | 2.13E-04 | 2.53E-02 |
| 20317 | Serpinf1 | mmu-miR-429-3p | 0.56 | 8.89E-04 | 6.53E-02 |
| 21859 | Timp3 | mmu-miR-429-3p | 0.53 | 1.69E-03 | 9.79E-02 |
| 76281 | Tax1bp3 | mmu-miR-429-3p | 0.59 | 4.36E-04 | 4.11E-02 |
| 20199 | S100a5 | mmu-miR-429-3p | 0.69 | 1.02E-05 | 3.54E-03 |
| 15312 | Hmgn1 | mmu-miR-429-3p | 0.61 | 2.05E-04 | 2.46E-02 |
| 20750 | Spp1 | mmu-miR-429-3p | 0.72 | 2.78E-06 | 1.44E-03 |
| 72075 | Ogfr | mmu-miR-429-3p | 0.56 | 7.74E-04 | 6.06E-02 |
| 238266 | Syt16 | mmu-miR-429-3p | -0.59 | 4.23E-04 | 4.08E-02 |
| 58875 | Hibadh | mmu-miR-429-3p | 0.68 | 2.02E-05 | 5.25E-03 |
| 13194 | Ddb1 | mmu-miR-429-3p | -0.58 | 5.72E-04 | 4.89E-02 |
| 574403 | EG574403 | mmu-miR-429-3p | 0.62 | 1.52E-04 | 2.05E-02 |
| 12404 | Cbln1 | mmu-miR-429-3p | 0.74 | 1.14E-06 | 7.91E-04 |
| 105844 | Card10 | mmu-miR-429-3p | 0.65 | 6.72E-05 | 1.21E-02 |
| 381113 | Cdkl4 | mmu-miR-429-3p | -0.55 | 1.19E-03 | 7.55E-02 |
| 320736 | E130203B14Rik | mmu-miR-429-3p | 0.57 | 7.13E-04 | 5.81E-02 |
| 14261 | Fmo1 | mmu-miR-429-3p | 0.74 | 1.05E-06 | 7.91E-04 |
| 68836 | Mrpl52 | mmu-miR-429-3p | 0.54 | 1.43E-03 | 8.53E-02 |
| 103712 | 6330403K07Rik | mmu-miR-429-3p | 0.58 | 4.72E-04 | 4.30E-02 |
| 66953 | Cdca7 | mmu-miR-429-3p | 0.55 | 1.10E-03 | 7.24E-02 |
| 13730 | Emp1 | mmu-miR-429-3p | 0.63 | 1.21E-04 | 1.82E-02 |
| 66704 | Rbm4b | mmu-miR-429-3p | 0.60 | 3.18E-04 | 3.39E-02 |
| 13392 | Dlx2 | mmu-miR-429-3p | 0.55 | 1.11E-03 | 7.24E-02 |
| 105827 | Amigo2 | mmu-miR-429-3p | 0.56 | 8.58E-04 | 6.53E-02 |
| 15478 | Hs3st3a1 | mmu-miR-429-3p | 0.63 | 1.25E-04 | 1.86E-02 |
| 101437 | Dhx32 | mmu-miR-429-3p | 0.66 | 4.27E-05 | 9.10E-03 |
| 211949 | Spsb4 | mmu-miR-429-3p | 0.61 | 1.95E-04 | 2.41E-02 |
| 21823 | Th | mmu-miR-429-3p | 0.69 | 1.36E-05 | 4.18E-03 |
| 14609 | Gja1 | mmu-miR-429-3p | 0.58 | 4.87E-04 | 4.30E-02 |
| 15214 | Hey2 | mmu-miR-429-3p | 0.57 | 6.33E-04 | 5.26E-02 |
| 70274 | Ly6g6e | mmu-miR-429-3p | 0.68 | 1.70E-05 | 4.70E-03 |
| 18295 | Ogn | mmu-miR-429-3p | 0.54 | 1.34E-03 | 8.19E-02 |
| 56615 | Mgst1 | mmu-miR-429-3p | 0.60 | 2.63E-04 | 2.99E-02 |
| 68039 | Nmb | mmu-miR-429-3p | 0.58 | 4.97E-04 | 4.34E-02 |
| 106957 | Slc39a6 | mmu-miR-429-3p | 0.58 | 5.29E-04 | 4.58E-02 |
| 22771 | Zic1 | mmu-miR-429-3p | 0.67 | 3.19E-05 | 7.56E-03 |
| 329831 | 4833436C18Rik | mmu-miR-429-3p | -0.62 | 1.75E-04 | 2.27E-02 |
| 15483 | Hsd11b1 | mmu-miR-429-3p | -0.53 | 1.71E-03 | 9.84E-02 |
| 319183 | Hist1h2bj | mmu-miR-429-3p | 0.65 | 6.48E-05 | 1.21E-02 |
| 70394 | Kptn | mmu-miR-429-3p | -0.54 | 1.37E-03 | 8.32E-02 |
| 66141 | Ifitm3 | mmu-miR-429-3p | 0.55 | 1.09E-03 | 7.24E-02 |
| 11826 | Aqp1 | mmu-miR-429-3p | 0.71 | 5.11E-06 | 2.12E-03 |
| 17171 | Mas1 | mmu-miR-429-3p | -0.61 | 2.41E-04 | 2.82E-02 |
| 69993 | Chn2 | mmu-miR-429-3p | 0.54 | 1.45E-03 | 8.58E-02 |
| 66797 | Cntnap2 | mmu-miR-429-3p | 0.55 | 1.12E-03 | 7.26E-02 |
| 18514 | Pbx1 | mmu-miR-429-3p | 0.67 | 2.54E-05 | 6.20E-03 |
| 20910 | Stxbp1 | mmu-miR-330-5p | 0.69 | 1.34E-05 | 6.66E-02 |
| 14661 | Lgsn | mmu-miR-330-5p | 0.68 | 2.11E-05 | 6.66E-02 |
| 70025 | Acot7 | mmu-miR-330-5p | 0.64 | 8.45E-05 | 8.77E-02 |
| 11993 | Aup1 | mmu-miR-330-5p | 0.65 | 6.37E-05 | 7.82E-02 |
| 11769 | Ap1s1 | mmu-miR-330-5p | 0.67 | 2.41E-05 | 6.66E-02 |
| 231148 | Ablim2 | mmu-miR-330-5p | 0.66 | 4.55E-05 | 7.56E-02 |
| 27643 | Ubl4 | mmu-miR-330-5p | 0.65 | 6.59E-05 | 7.82E-02 |
| 17760 | Mtap6 | mmu-miR-330-5p | 0.66 | 3.77E-05 | 7.56E-02 |
| 13618 | Ednrb | mmu-miR-96-5p | 0.65 | 4.87E-05 | 1.03E-02 |
| 69195 | Tmem121 | mmu-miR-96-5p | -0.54 | 1.48E-03 | 9.00E-02 |
| 13193 | Dcx | mmu-miR-96-5p | 0.56 | 7.66E-04 | 5.83E-02 |
| 14199 | Fhl1 | mmu-miR-96-5p | 0.65 | 4.97E-05 | 1.03E-02 |
| 83436 | Plekha2 | mmu-miR-96-5p | 0.53 | 1.64E-03 | 9.26E-02 |
| 12891 | Cpne6 | mmu-miR-96-5p | 0.62 | 1.57E-04 | 2.00E-02 |
| 58802 | Kcnmb4 | mmu-miR-96-5p | -0.62 | 1.33E-04 | 1.84E-02 |
| 245857 | Ssh3 | mmu-miR-96-5p | -0.57 | 6.88E-04 | 5.44E-02 |
| 20014 | Rpn2 | mmu-miR-96-5p | 0.64 | 8.12E-05 | 1.40E-02 |
| 70351 | Ppp4r1 | mmu-miR-96-5p | 0.57 | 5.96E-04 | 4.90E-02 |
| 14415 | Gad1 | mmu-miR-96-5p | 0.76 | 5.66E-07 | 5.22E-04 |
| 72265 | Tram1 | mmu-miR-96-5p | 0.53 | 1.69E-03 | 9.46E-02 |
| 76974 | 1190003J15Rik | mmu-miR-96-5p | -0.56 | 9.33E-04 | 6.85E-02 |
| 100019 | Mdn1 | mmu-miR-96-5p | 0.62 | 1.71E-04 | 2.02E-02 |
| 170718 | Idh3b | mmu-miR-96-5p | -0.53 | 1.63E-03 | 9.26E-02 |
| 107448 | Unc5a | mmu-miR-96-5p | -0.59 | 3.89E-04 | 3.71E-02 |
| 68273 | Pomgnt1 | mmu-miR-96-5p | -0.63 | 1.02E-04 | 1.61E-02 |
| 11514 | Adcy8 | mmu-miR-96-5p | 0.62 | 1.46E-04 | 1.95E-02 |
| 213019 | Pdlim2 | mmu-miR-96-5p | -0.54 | 1.50E-03 | 9.04E-02 |
| 210711 | 1110007A13Rik | mmu-miR-96-5p | 0.59 | 3.40E-04 | 3.45E-02 |
| 217125 | Samd14 | mmu-miR-96-5p | 0.64 | 7.17E-05 | 1.35E-02 |
| 225849 | Ppp2r5b | mmu-miR-96-5p | -0.65 | 5.57E-05 | 1.11E-02 |
| 22225 | Usp5 | mmu-miR-96-5p | -0.56 | 9.75E-04 | 6.92E-02 |
| 14537 | Gcnt1 | mmu-miR-96-5p | 0.57 | 6.48E-04 | 5.22E-02 |
| 14664 | Slc6a9 | mmu-miR-96-5p | 0.60 | 3.10E-04 | 3.30E-02 |
| 59035 | Carm1 | mmu-miR-96-5p | -0.56 | 9.74E-04 | 6.92E-02 |
| 67971 | Tppp3 | mmu-miR-96-5p | -0.60 | 3.08E-04 | 3.30E-02 |
| 216345 | Zfc3h1 | mmu-miR-96-5p | 0.68 | 2.04E-05 | 4.84E-03 |
| 234776 | Atmin | mmu-miR-96-5p | -0.53 | 1.62E-03 | 9.26E-02 |
| 12632 | Cfl2 | mmu-miR-96-5p | -0.55 | 1.14E-03 | 7.48E-02 |
| 140919 | Slc17a6 | mmu-miR-96-5p | 0.55 | 1.19E-03 | 7.70E-02 |
| 22348 | Slc32a1 | mmu-miR-96-5p | 0.63 | 1.02E-04 | 1.61E-02 |
| 20295 | Ccl17 | mmu-miR-96-5p | -0.59 | 3.32E-04 | 3.41E-02 |
| 14706 | Gng4 | mmu-miR-96-5p | 0.70 | 9.29E-06 | 3.09E-03 |
| 53599 | Cd164 | mmu-miR-96-5p | 0.58 | 4.88E-04 | 4.32E-02 |
| 20254 | Scg2 | mmu-miR-96-5p | 0.57 | 5.84E-04 | 4.90E-02 |
| 75104 | Mmd2 | mmu-miR-96-5p | 0.56 | 8.58E-04 | 6.42E-02 |
| 239217 | Kctd12 | mmu-miR-96-5p | 0.69 | 1.12E-05 | 3.43E-03 |
| 105859 | Csdc2 | mmu-miR-96-5p | 0.73 | 2.19E-06 | 1.21E-03 |
| 29861 | Dpf1 | mmu-miR-96-5p | -0.53 | 1.62E-03 | 9.26E-02 |
| 56699 | Cdc42ep4 | mmu-miR-96-5p | 0.61 | 2.04E-04 | 2.32E-02 |
| 12362 | Casp1 | mmu-miR-96-5p | 0.71 | 4.55E-06 | 1.99E-03 |
| 12111 | Bgn | mmu-miR-96-5p | 0.68 | 1.75E-05 | 4.31E-03 |
| 15360 | Hmgcs2 | mmu-miR-96-5p | 0.62 | 1.70E-04 | 2.02E-02 |
| 11749 | Anxa6 | mmu-miR-96-5p | -0.55 | 1.03E-03 | 7.13E-02 |
| 11933 | Atp1b3 | mmu-miR-96-5p | 0.55 | 1.07E-03 | 7.31E-02 |
| 68801 | Elovl5 | mmu-miR-96-5p | 0.63 | 1.03E-04 | 1.61E-02 |
| 26401 | Map3k1 | mmu-miR-96-5p | 0.74 | 1.11E-06 | 7.11E-04 |
| 12308 | Calb2 | mmu-miR-96-5p | 0.84 | 2.32E-09 | 9.65E-06 |
| 14115 | Fbln2 | mmu-miR-96-5p | 0.60 | 3.15E-04 | 3.31E-02 |
| 16206 | Lrig1 | mmu-miR-96-5p | 0.58 | 4.94E-04 | 4.32E-02 |
| 232441 | Rerg | mmu-miR-96-5p | 0.69 | 1.21E-05 | 3.45E-03 |
| 233552 | Gdpd5 | mmu-miR-96-5p | 0.54 | 1.57E-03 | 9.26E-02 |
| 68738 | Acss1 | mmu-miR-96-5p | 0.55 | 1.12E-03 | 7.48E-02 |
| 17268 | Meis1 | mmu-miR-96-5p | 0.70 | 6.99E-06 | 2.44E-03 |
| 245386 | 6430550H21Rik | mmu-miR-96-5p | 0.65 | 6.02E-05 | 1.16E-02 |
| 19699 | Reln | mmu-miR-96-5p | 0.79 | 1.02E-07 | 1.11E-04 |
| 70762 | Dclk2 | mmu-miR-96-5p | 0.54 | 1.37E-03 | 8.52E-02 |
| 13179 | Dcn | mmu-miR-96-5p | 0.54 | 1.40E-03 | 8.60E-02 |
| 74020 | Cpne4 | mmu-miR-96-5p | 0.69 | 1.49E-05 | 3.88E-03 |
| 17110 | Lyz | mmu-miR-96-5p | 0.58 | 4.90E-04 | 4.32E-02 |
| 50771 | Atp9b | mmu-miR-96-5p | -0.55 | 1.04E-03 | 7.13E-02 |
| 320333 | D830030K20Rik | mmu-miR-96-5p | -0.57 | 6.72E-04 | 5.36E-02 |
| 78908 | Igsf3 | mmu-miR-96-5p | 0.54 | 1.47E-03 | 8.97E-02 |
| 60425 | Doc2g | mmu-miR-96-5p | 0.80 | 3.08E-08 | 5.12E-05 |
| 227325 | Dner | mmu-miR-96-5p | 0.56 | 9.76E-04 | 6.92E-02 |
| 78593 | Nrip3 | mmu-miR-96-5p | 0.61 | 1.88E-04 | 2.20E-02 |
| 13390 | Dlx1 | mmu-miR-96-5p | 0.78 | 1.07E-07 | 1.11E-04 |
| 170574 | Sp7 | mmu-miR-96-5p | 0.69 | 1.18E-05 | 3.45E-03 |
| 107976 | Bre | mmu-miR-96-5p | -0.53 | 1.82E-03 | 9.92E-02 |
| 170677 | Pcdh21 | mmu-miR-96-5p | 0.83 | 4.07E-09 | 1.13E-05 |
| 100047353 | LOC100047353 | mmu-miR-96-5p | 0.68 | 1.76E-05 | 4.31E-03 |
| 72102 | Dusp11 | mmu-miR-96-5p | 0.60 | 3.07E-04 | 3.30E-02 |
| 67839 | Gpsm1 | mmu-miR-96-5p | 0.64 | 7.98E-05 | 1.40E-02 |
| 18829 | Ccl21a | mmu-miR-96-5p | -0.63 | 1.26E-04 | 1.77E-02 |
| 93695 | Gpnmb | mmu-miR-96-5p | 0.53 | 1.74E-03 | 9.63E-02 |
| 60510 | Syt9 | mmu-miR-96-5p | 0.60 | 3.07E-04 | 3.30E-02 |
| 140723 | Cacng5 | mmu-miR-96-5p | 0.73 | 2.32E-06 | 1.21E-03 |
| 12226 | Btg1 | mmu-miR-96-5p | 0.82 | 1.14E-08 | 2.37E-05 |
| 15081 | H3f3b | mmu-miR-96-5p | 0.65 | 5.62E-05 | 1.11E-02 |
| 22773 | Zic3 | mmu-miR-96-5p | 0.59 | 4.16E-04 | 3.84E-02 |
| 209086 | Samd9l | mmu-miR-96-5p | 0.64 | 8.11E-05 | 1.40E-02 |
| 50793 | Orc3l | mmu-miR-96-5p | 0.70 | 9.72E-06 | 3.10E-03 |
| 100047583 | LOC100047583 | mmu-miR-96-5p | 0.75 | 6.34E-07 | 5.27E-04 |
| 319180 | Hist1h2bf | mmu-miR-96-5p | 0.66 | 4.15E-05 | 9.31E-03 |
| 214189 | Scgn | mmu-miR-96-5p | 0.74 | 1.04E-06 | 7.11E-04 |
| 18516 | Pbx3 | mmu-miR-96-5p | 0.53 | 1.64E-03 | 9.26E-02 |
| 231086 | Hadhb | mmu-miR-96-5p | 0.63 | 1.13E-04 | 1.73E-02 |
| 70536 | Qpct | mmu-miR-96-5p | 0.62 | 1.62E-04 | 2.01E-02 |
| 22044 | Trh | mmu-miR-96-5p | 0.87 | 1.07E-10 | 8.87E-07 |
| 320145 | Sp8 | mmu-miR-96-5p | 0.72 | 3.66E-06 | 1.79E-03 |
| 13446 | Doc2a | mmu-miR-96-5p | -0.58 | 4.41E-04 | 4.02E-02 |
| 12793 | Cnih | mmu-miR-96-5p | -0.54 | 1.28E-03 | 8.03E-02 |
| 14617 | Gjd2 | mmu-miR-96-5p | 0.58 | 5.57E-04 | 4.72E-02 |
| 96979 | Ptges2 | mmu-miR-96-5p | -0.63 | 1.19E-04 | 1.73E-02 |
| 22421 | Wnt7a | mmu-miR-96-5p | 0.63 | 1.16E-04 | 1.73E-02 |
| 12122 | Bid | mmu-miR-96-5p | 0.61 | 1.93E-04 | 2.23E-02 |
| 66659 | Acp6 | mmu-miR-96-5p | -0.55 | 1.13E-03 | 7.48E-02 |
| 12988 | Csk | mmu-miR-96-5p | 0.59 | 3.59E-04 | 3.51E-02 |
| 320487 | Heatr5a | mmu-miR-96-5p | 0.58 | 4.69E-04 | 4.23E-02 |
| 59093 | Pcbp3 | mmu-miR-96-5p | 0.60 | 3.20E-04 | 3.32E-02 |
| 213391 | Rassf4 | mmu-miR-96-5p | 0.58 | 5.28E-04 | 4.57E-02 |
| 12836 | Col7a1 | mmu-miR-96-5p | 0.55 | 1.26E-03 | 7.99E-02 |
| 21753 | Tes | mmu-miR-96-5p | 0.59 | 4.12E-04 | 3.84E-02 |
| 545279 | E530011F12Rik | mmu-miR-96-5p | 0.79 | 8.51E-08 | 1.11E-04 |
| 64075 | Smoc1 | mmu-miR-96-5p | 0.62 | 1.58E-04 | 2.00E-02 |
| 20317 | Serpinf1 | mmu-miR-96-5p | 0.57 | 7.18E-04 | 5.63E-02 |
| 21859 | Timp3 | mmu-miR-96-5p | 0.55 | 1.22E-03 | 7.88E-02 |
| 76281 | Tax1bp3 | mmu-miR-96-5p | 0.56 | 9.58E-04 | 6.92E-02 |
| 20199 | S100a5 | mmu-miR-96-5p | 0.75 | 8.80E-07 | 6.64E-04 |
| 11785 | Apbb1 | mmu-miR-96-5p | -0.56 | 8.80E-04 | 6.53E-02 |
| 15312 | Hmgn1 | mmu-miR-96-5p | 0.59 | 3.67E-04 | 3.55E-02 |
| 23956 | Neu2 | mmu-miR-96-5p | -0.54 | 1.37E-03 | 8.52E-02 |
| 20750 | Spp1 | mmu-miR-96-5p | 0.71 | 6.31E-06 | 2.44E-03 |
| 72075 | Ogfr | mmu-miR-96-5p | 0.54 | 1.58E-03 | 9.26E-02 |
| 238266 | Syt16 | mmu-miR-96-5p | -0.63 | 1.24E-04 | 1.77E-02 |
| 58875 | Hibadh | mmu-miR-96-5p | 0.69 | 1.27E-05 | 3.50E-03 |
| 13194 | Ddb1 | mmu-miR-96-5p | -0.59 | 3.45E-04 | 3.45E-02 |
| 574403 | EG574403 | mmu-miR-96-5p | 0.62 | 1.36E-04 | 1.85E-02 |
| 29876 | Clic4 | mmu-miR-96-5p | 0.53 | 1.78E-03 | 9.79E-02 |
| 12404 | Cbln1 | mmu-miR-96-5p | 0.74 | 1.28E-06 | 7.60E-04 |
| 105844 | Card10 | mmu-miR-96-5p | 0.63 | 1.17E-04 | 1.73E-02 |
| 320736 | E130203B14Rik | mmu-miR-96-5p | 0.62 | 1.59E-04 | 2.00E-02 |
| 14261 | Fmo1 | mmu-miR-96-5p | 0.70 | 7.06E-06 | 2.44E-03 |
| 110253 | Triobp | mmu-miR-96-5p | 0.54 | 1.26E-03 | 7.99E-02 |
| 103712 | 6330403K07Rik | mmu-miR-96-5p | 0.55 | 1.17E-03 | 7.66E-02 |
| 74194 | Rnd3 | mmu-miR-96-5p | 0.57 | 7.46E-04 | 5.73E-02 |
| 66953 | Cdca7 | mmu-miR-96-5p | 0.53 | 1.70E-03 | 9.46E-02 |
| 13730 | Emp1 | mmu-miR-96-5p | 0.62 | 1.48E-04 | 1.95E-02 |
| 66704 | Rbm4b | mmu-miR-96-5p | 0.64 | 8.82E-05 | 1.50E-02 |
| 13392 | Dlx2 | mmu-miR-96-5p | 0.55 | 1.02E-03 | 7.12E-02 |
| 105827 | Amigo2 | mmu-miR-96-5p | 0.55 | 9.99E-04 | 7.03E-02 |
| 15478 | Hs3st3a1 | mmu-miR-96-5p | 0.62 | 1.71E-04 | 2.02E-02 |
| 237759 | Col23a1 | mmu-miR-96-5p | 0.56 | 8.39E-04 | 6.33E-02 |
| 68087 | Dcakd | mmu-miR-96-5p | 0.57 | 5.91E-04 | 4.90E-02 |
| 101437 | Dhx32 | mmu-miR-96-5p | 0.69 | 1.41E-05 | 3.78E-03 |
| 211949 | Spsb4 | mmu-miR-96-5p | 0.63 | 9.87E-05 | 1.61E-02 |
| 21823 | Th | mmu-miR-96-5p | 0.71 | 5.02E-06 | 2.08E-03 |
| 14609 | Gja1 | mmu-miR-96-5p | 0.59 | 3.94E-04 | 3.71E-02 |
| 15214 | Hey2 | mmu-miR-96-5p | 0.60 | 2.83E-04 | 3.17E-02 |
| 70274 | Ly6g6e | mmu-miR-96-5p | 0.71 | 6.57E-06 | 2.44E-03 |
| 56615 | Mgst1 | mmu-miR-96-5p | 0.59 | 3.49E-04 | 3.45E-02 |
| 68039 | Nmb | mmu-miR-96-5p | 0.58 | 5.35E-04 | 4.58E-02 |
| 22771 | Zic1 | mmu-miR-96-5p | 0.66 | 4.45E-05 | 9.73E-03 |
| 329831 | 4833436C18Rik | mmu-miR-96-5p | -0.57 | 7.33E-04 | 5.69E-02 |
| 319183 | Hist1h2bj | mmu-miR-96-5p | 0.57 | 6.21E-04 | 5.05E-02 |
| 70394 | Kptn | mmu-miR-96-5p | -0.53 | 1.64E-03 | 9.26E-02 |
| 11826 | Aqp1 | mmu-miR-96-5p | 0.71 | 4.31E-06 | 1.99E-03 |
| 11836 | Araf | mmu-miR-96-5p | 0.54 | 1.58E-03 | 9.26E-02 |
| 17171 | Mas1 | mmu-miR-96-5p | -0.64 | 7.50E-05 | 1.38E-02 |
| 69993 | Chn2 | mmu-miR-96-5p | 0.55 | 1.13E-03 | 7.48E-02 |
| 18514 | Pbx1 | mmu-miR-96-5p | 0.68 | 2.22E-05 | 5.12E-03 |
| 16524 | Kcnj9 | mmu-miR-34a-5p | 0.65 | 5.25E-05 | 7.48E-02 |
| 216874 | Camta2 | mmu-miR-34a-5p | 0.64 | 7.56E-05 | 8.67E-02 |
| 56807 | Scamp5 | mmu-miR-34a-5p | 0.66 | 4.03E-05 | 7.48E-02 |
| 118452 | Baalc | mmu-miR-34a-5p | 0.66 | 3.83E-05 | 7.48E-02 |
| 56085 | Ubqln1 | mmu-miR-34a-5p | 0.64 | 8.81E-05 | 8.67E-02 |
| 230757 | 5730409E04Rik | mmu-miR-34a-5p | -0.64 | 9.39E-05 | 8.67E-02 |
| 17760 | Mtap6 | mmu-miR-34a-5p | 0.69 | 1.36E-05 | 5.64E-02 |
| 27407 | Abcf2 | mmu-miR-34a-5p | 0.69 | 1.34E-05 | 5.64E-02 |
| 207278 | Fchsd2 | mmu-miR-34a-5p | -0.65 | 5.40E-05 | 7.48E-02 |
| 52708 | Zfp410 | mmu-miR-34a-5p | 0.63 | 1.14E-04 | 9.51E-02 |
